# A six-hour time series of the acute effects of ingested cannabis intoxication on a battery of cognitive-motor tasks

**DOI:** 10.1371/journal.pone.0353389

**Published:** 2026-08-13

**Authors:** Paige V. Copeland, Quinn Malone, Brian H. Dalton, Chris J. McNeil

**Affiliations:** School of Health and Exercise Sciences, The University of British Columbia, Kelowna, British Columbia, Canada; NYU Grossman School of Medicine: New York University School of Medicine, UNITED STATES OF AMERICA

## Abstract

The psychoactive component of cannabis, Δ9-tetrahydrocannabinol (THC), is known to alter cognitive-motor function; however, despite the high prevalence of cannabis use globally, the time course of the effects of ingesting THC is unclear. To allow future studies to accurately time the assessment of the impacts of cannabis on behaviour, this study aimed to establish a detailed time series of subjective intoxication and cognitive-motor performance following THC ingestion. Fifteen infrequent cannabis users (8 females; 29.4 ± 8.1 y) completed a testing battery before (baseline) and at 11 timepoints (0-6 h) after ingesting 10 mg of THC. A subset of seven participants (3 females; 32.0 ± 8.8 y) also performed an identical control session, with no drug administered. Subjective drug effects and cardiovascular responses were recorded, and cognitive-motor function was measured via performance on simple reaction time, go/no-go reaction time, and Corsi block tapping tasks. Linear mixed-effects models were used to determine if subjective and objective measures changed during the time series. Compared to baseline, participants reported significant subjective drug effects 1-6 h post-ingestion. Heart rate was elevated from 3-6 h, whereas blood pressure was elevated at timepoints between 1-4 h. Individuals demonstrated a robust increase of simple reaction time from 1-6 h, reduced go/no-go accuracy between 1.5-6 h, and impaired Corsi blocking tapping performance between 1-5 h. Overall, the ideal window to test the cognitive-motor effects of cannabis intoxication appears at 3-4 h post-THC ingestion, with a marked perception of feeling high between 2-5 h.

## Introduction

Although cannabis is believed to have been used as a medicine for nearly 5000 years [1], criminalization of the drug in the 20^th^ century greatly curtailed scientific evaluation, leaving much unknown about the acute and chronic effects of cannabis on human function. As recreational cannabis use continues to be legalized across the world, it has become increasingly important to advance our knowledge about the effects of intoxication arising from the consumption of the main psychoactive component of cannabis, Δ9-tetrahydrocannabinol (THC) [2,3]. Although inhalation remains the most common form of usage in Canada [4], ingested cannabis (colloquially referred to as ‘edibles’) is increasingly popular, as 55% of cannabis users report consuming edibles [4], and numerous options (e.g., drinks, gummies, capsules, etc.) are commercially available. As inhalation and ingestion result in different pharmacokinetic and pharmacodynamic outcomes for the psychoactive components of cannabis [5–7], there is a need to understand the acute effects of ingested THC. However, there is limited information about the effects of this mode of consumption on cognitive-motor function [6,8,9].

Although firm conclusions concerning cognitive-motor performance during acute cannabis intoxication are difficult to draw due to the heterogeneity of previous study designs, some results appear robust. For example, according to a recent meta-analysis, when driving, individuals who have consumed cannabis have impaired lateral control and drive more slowly than sober individuals [10]. When considering less complex behaviours, though null effects have been reported with ingested THC [6,8], simple reaction time (SRT) is impaired after inhalation [11,12]. Similarly, inhaling cannabis increases the number of response errors during “no-go” trials of a go/no-go (GNG) task [13,14]. Short-term memory is also impaired acutely by cannabis inhalation [11,15,16]. Other cognitive tasks which have been repeatedly examined following ingestion of THC-containing products include the digit symbol substitution task, divided attention task, and paced auditory serial addition task. Results from these tasks have been mixed, with both null and negative effects following THC ingestion being reported for all associated outcome measures [9,17,18].

Though inhalation offers potentially higher THC bioavailability [19], edibles are an appealing method of cannabis consumption to use in research because the bioavailability of THC from edibles occurs within a narrower range (~4–20% for edibles compared to ~2–56% for smoked) [19], THC and other cannabinoid dosages can be highly controlled, there are fewer perceived health risks [4], and there is a prolonged period of intoxication [6,8,9]. To aid future research into the acute effects of commercially-available edible THC products, a time course (i.e., onset and offset) of indices of intoxication should be established. Thus, the aim of this study was to evaluate numerous subjective and objective measures for six hours following ingestion of 10 mg of THC. We hypothesised that effects of intoxication would be evident after ~1 h and last until ~4 h after ingestion.

## Materials and methods

### Participants

The study was approved by the Clinical Research Ethics Board at the University of British Columbia (H23-00249) and conducted in accordance with Canada’s Tri-Council Policy Statement: Ethical Conduct for Research involving Humans and the Declaration of Helsinki. Additionally, this research was approved by Health Canada and licensed under the Canadian Cannabis Act and Cannabis Regulations (LIC-4FN4K9EH76–2024). Fifteen healthy individuals (29.4 ± 8.1 y, 174.3 ± 10.6 cm, 72.1 ± 18.0 kg; 8 females; see Table 1 for full demographic data) participated in the intervention session (INT). Seven of those participants (32.0 ± 8.8 y, 171.1 ± 9.9 cm, 65.4 ± 10.6 kg; 3 females) also completed an additional control session (CON), during which no intervention was administered. Accordingly, participants were not blinded to the nature of the intervention during either session. To limit the influence of chronic cannabis use on the effects of acute intoxication [20], participants were recruited from a population characterized as infrequent users, which was defined as not having used cannabis approximately more than once per week during the previous six months [8,21]. To reduce the risk of adverse events caused by acute cannabis use (e.g., anxiety, paranoia, nausea, hallucinations) [5], individuals who had never consumed THC, and were thus unaware if they may be predisposed to potential adverse effects of cannabis consumption, were excluded from participation. Additional inclusion/exclusion criteria included that participants: were between 21–50 years of age; could read, write, and speak English; did not have a cardiopulmonary disease with hypertension, syncope, or tachycardia; did not have a liver or kidney disease; did not have a personal history of substance use disorders; did not have a personal or family history of schizophrenia; did not have any dietary restrictions that prevented them from consuming pig-derived gelatin or the colourants used in the cannabis product; were not participating in any other experimental trial involving cannabis; and were not trying to become pregnant, were not pregnant, and were not breastfeeding. To confirm that no participant was administered a cannabis product while unknowingly pregnant, any individual who identified as having child-bearing potential was given a take-home pregnancy test and reported the result before their INT session.

**Table 1:** Participant characteristics.

	Measure	Group (INT n = 15, CON n = 7)		Females (INT n = 8, CON n = 3)		Males (INT n = 7, CON n = 4)	
M ± SD	Range	M ± SD	Range	M ± SD	Range
**INT Group**	Age (y)	29.4 ± 8.1	21-47	26.1 ± 4.1	21-32	33.1 ± 8.8	24-47
	Height (cm)	174.3 ± 10.6	157-199	165.6 ± 7.0	157-174	184.1 ± 9.8†	171-199
	Body mass (kg)	72.1 ± 18.0	50-113	68.1 ± 19.6	57-113	76.6 ± 9.9	61-89
	Caffeine consumption (beverages/day)	1.6 ± 1.9	0-7	1.3 ± 1.0	0-2.5	1.9 ± 2.6	0-7
**CON Subgroup**	Age (y)	32.0 ± 8.8	22-47	27.3 ± 5.0	22-32	35.5 ± 11.2	24-47
	Height (cm)	171.1 ± 9.9	157-183	162.3 ± 9.2	157-173	177.8 ± 6.2	171-183
	Body mass (kg)	65.4 ± 10.6	50-82	57 ± 7	50-64	71.8 ± 10.3	61-82
	Caffeine consumption (beverages/day)	2.2 ± 2.3	0-7	1.5 ± 0.9	1-2.5	2.8 ± 3.1	0-7
**DFAQ-CU Variables**	Frequency of cannabis use (d/y)	4.8 ± 5.2	0-15	8.1 ± 5.5	0-15	1.1 ± 0.9†	0-3
	Age of cannabis use onset (y)	18.6 ± 3.4	13-25	18.5 ± 4.5	13-25	18.7 ± 2.2	17-22

All values are presented as the mean ± one standard deviation. Abbreviations: INT, intervention session; CON, control session; M, Mean; SD, standard deviation; DFAQ-CU, Daily Sessions, Frequency, Age of Onset, and Quantity of Cannabis Use Inventory. † denotes significant difference from females in the same group (p < 0.001).

Several participants had used psychoactive substances other than cannabis, though none had used one of these substances at least 5 months prior to participation. Psilocybin-containing mushrooms were the most common substance (INT: N = 8, CON: N = 4), followed by 3,4-Methylenedioxymethamphetamine (MDMA/ecstasy/molly; INT: N = 2, CON: N = 1). Lysergic acid diethylamide (LSD, INT: N = 1, CON = 0) and cocaine (INT: N = 1, CON: N = 1) were also reported.

### Familiarization, experimental, and control sessions

**Fig 1:**
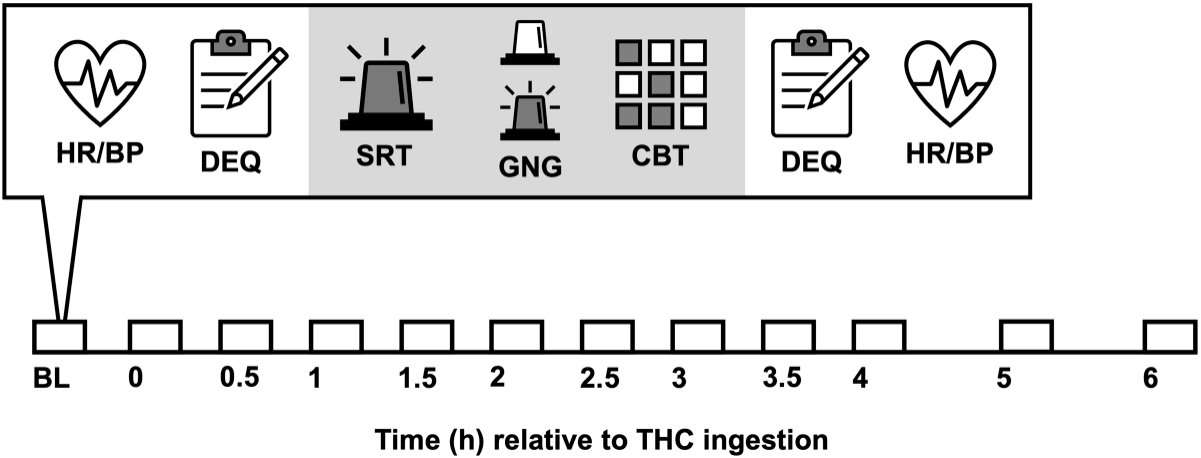
Study protocol. The bottom of the schematic depicts the timing of testing blocks for subjective and objective measures of intoxication, which are shown in the breakout box (top). The order of heart rate (HR) and blood pressure (BP) measurements, as well as administration of the Drug Effects Questionnaire (DEQ) was consistent among all participants. The cognitive-motor tasks (shaded area), comprised of simple reaction time (SRT), Go/No-Go (GNG) reaction time, and Corsi block tapping (CBT), were performed in a pseudo-randomized order and counterbalanced across participants. The baseline testing block (BL) concluded ~10 min before the ingestion of the intervention (10 mg THC) at 0 h.

No more than seven days prior to the first testing session (i.e., INT or CON), participants attended a familiarization session of ~60 min. After providing written and oral consent, participants completed the Daily Sessions, Frequency, Age of Onset, and Quantity of Cannabis Use Inventory (DFAQ-CU) to contextualize their personal history with the drug [22]. Next, individuals performed one full block of the testing battery (see below). Of the seven participants who took part in both sessions, four completed the CON session first. Sessions were separated by at least one week to allow for intervention washout [8,23]. Participants were asked to abstain from consuming cannabis or any other psychoactive substances for at least a week before a testing session (and between sessions for those who completed both), and to abstain from alcohol consumption for at least 12 h before each testing session. No toxicology screens were performed to ensure compliance; participant self-report was considered sufficient. Participants were also not permitted to smoke during either session, and none expressed a desire to do so. The INT and CON sessions were structured similarly (Fig 1), except that no intervention was administered during the CON session.

### Cannabis product

For the INT session, participants ingested two softgel capsules containing 5 mg of THC each (Redecan 5:0 Gems^TM^, Tilray Brands Inc., New York City, USA), for a total of 10 mg THC. We chose 10 mg of THC because it is considered a low-to-moderate dosage [24,25], and it appears to be the highest tested standardized dose that is unlikely to lead to adverse events, such as acute anxiety, paranoia, or emesis [5]. Further, in Canada, 10 mg is the maximal amount of THC that can be sold in a single package of a concentrated cannabis product (e.g., softgel capsules, gummies, etc.). The intervention was a plant extract, and other ingredients include medium chain triglycerides oil, gelatine, water, glycerine, Tartrazine Lake (a dye), and Brilliant Blue FCF (a dye). Though the product is intended to have no CBD, the manufacturer states that there may be between 0–1 mg CBD in each capsule. As the product is a plant extract and the manufacturer cannot rule out the presence of CBD, it is likely that traces of other naturally-occurring cannabinoids were also present in the intervention. Through pilot testing, the 10 mg dose of THC was established to be sufficient to induce common symptoms of acute cannabis intoxication (i.e., altered mood and perception).

### Study design

Testing sessions were started in the morning (9:00–11:30AM) and ended in the afternoon (4:00–6:30PM). During the INT and CON sessions, 12 testing blocks were conducted (Fig 1), each consisting of a set experimental battery. Ten minutes after completion of the first testing block (baseline; BL), at 0 h, participants consumed the THC capsules (INT) or nothing (CON). Immediately afterward, the 0-h testing block began, with subsequent blocks starting 0.5, 1, 1.5, 2 2.5, 3, 3.5, 4, 5, and 6 h later.

Each testing block took 15–20 min to complete, and began with a measurement of heart rate (HR) and blood pressure (BP), followed by a subjective rating of drug effects via the Drug Effects Questionnaire (DEQ) [26]. Next, participants completed a battery of three cognitive-motor tasks, with the order pseudo-randomized and counterbalanced across participants, but fixed for each participant for all testing blocks during both INT and CON sessions. To conclude the testing block, participants gave another subjective rating of drug effects and again had HR and BP measured. During the 10–15 min breaks between testing blocks, participants were instructed to refrain from activities that could lead to increased levels of mental or emotional arousal (e.g., working on a personal computer, watching emotionally-stimulating media). Participants mainly opted to engage in sedentary activities (e.g., conversing with the investigators, watching emotionally-neutral documentaries), though some partook in supervised walks in and around the building containing the testing room. To limit interindividual differences of gastrointestinal absorption of THC due to food consumption [27], participants were prohibited from eating or drinking anything but water from the start of BL until the conclusion of the testing block at 2 h. Concerning food consumption prior to each testing session, participants were asked to eat their normal breakfast. During testing breaks between 2 and 6 h, participants were allowed to eat and drink ad libitum. All but one participant opted to eat during the breaks between 2 and 3 h.

### Subjective responses

To quantify subjective intoxication, participants completed a five-item version of the DEQ [26], which included the following prompts: “Do you feel a drug effect right now?”; “Are you high right now?”; “Do you dislike any of the effects you are feeling right now?”; “Do you like any of the effects you are feeling right now?”; and “Would you like more of the drug you took, right now?”. For each prompt, participants were instructed to draw a single vertical line along a 100-mm visual analog scale that ranged from 0 (Not at all) to 100 (Extremely). For each DEQ item, statistical analysis was conducted using the mean value of the two ratings obtained within each testing block.

### Cardiovascular responses

An automated upper-arm BP monitor (BP7450CAN, OMRON Healthcare Co., Ltd., Kyoto, Japan) was used to record HR as well as systolic and diastolic BP. As with the DEQ items, statistical analyses of HR and BP data were conducted using the mean of the values recorded at the start and end of each testing block. Due to technical limitation of the monitor (which was unable to measure HR or BP for individuals with a HR below 40 beats-per-minute; bpm), data from one individual were unable to be sampled during their control session; therefore, the control cardiovascular dataset has a sample of six participants.

### Cognitive-motor tasks

During each testing block, participants sat at a desk to complete the following three cognitive-motor tasks: SRT, GNG, and Corsi block tapping (CBT). Using Presentation software (v23.1, Neurobehavioral Systems Inc., Berkeley, USA), the first three tasks were completed on a laptop computer positioned ~60 cm in front of the participant’s face. A wireless mouse held in the right hand was used for each task.

The SRT task measured the speed required to perceive and trigger a response to a stimulus [28], which was a red square appearing in the centre of the screen. The task consisted of 45 visual stimuli presented with interstimulus intervals of 2000–3000 ms. Participants were instructed to respond as quickly as possible to the stimulus by clicking the left mouse button, after which the stimulus disappeared. If participants did not respond within 500 ms, the stimulus would disappear and the next interstimulus interval would begin. Responses made >1000 ms after stimulus onset was not considered a valid reaction time (RT), nor was a response made within 100 ms of stimulus onset, which was considered to be anticipatory. Invalid RTs were excluded from analysis. Only a small proportion of trials were rejected for these reasons (0.30% and 0.43% of INT and CON datasets, respectively). From the SRT task, a participant’s mean RT and intraindividual RT variability were calculated each testing block.

The GNG task examined inhibitory control [28]. It consisted of 96 “go” and 48 “no-go” stimuli that were presented with an interstimulus interval of 700 ms. The “go” stimuli were blue, green, or red squares (32 of each), to which the participants were instructed to respond as quickly as possible by clicking the left button on the mouse, after which the stimulus disappeared. The “no-go” stimulus consisted of a black square, to which the participants were instructed to not respond and to wait until it disappeared from the screen. All stimuli to which participants did not respond were displayed for 1000 ms before disappearing. For each testing block, mean RT and intraindividual RT variability were derived for the “go” stimuli. Accuracy for “no-go” stimuli was expressed as the percent of “no-go” stimuli correctly avoided.

The CBT test examined short-term memory [29]. It involved the presentation of nine squares on screen, which lit up in a pseudorandom sequence during each trial. After the sequence finished, participants were instructed to replicate the order by clicking each square using the left mouse button. The sequence length began at three squares, with participants presented three unique trials. If participants were successful at reproducing two of the three sequences, the sequence length would increase by one, and three new trials would be presented. The test ended when a participant failed to replicate two sequences at a given level. The highest level achieved (i.e., maximum remembered span) was the outcome measure.

### Statistical analysis

To test for differences of subjective intoxication, cardiovascular responses, and cognitive-motor task performance over time, linear mixed-effects models were used. The models included the outcome measure as the dependent variable, as well as testing block (BL, 0, 0.5, 1, 1.5, 2, 2.5, 3, 3.5, 4, 5, 6 h) and sex (female or male; F or M, respectively) as fixed-effect factors. BL was set as the baseline comparator for the testing block factor, and participant was set as a cluster variable (i.e., a random-effect factor). Two different models were used; one for the subjective responses (DEQ outcomes) and another for the objective responses (cognitive-motor and cardiovascular outcomes). The two models were identical, except that the one used for the subjective responses did not include the CON session dataset as a covariate because participants were not asked to consume anything, which meant all answers to the DEQ prompts were zero (Not at all). The inclusion of the CON session dataset as a covariate for the objective measures allowed us to control for: 1) natural variability of cognitive-motor performance and cardiovascular responses over the course of the 7-h testing session, and 2) any learning which may have occurred as a result of repeatedly performing the tasks. Statistical comparisons were not made between the CON and INT datasets nor across time within the CON session.

All statistical tests were completed using jamovi (v. 2.6.25.0) [30]. The linear mixed-effects models were completed using the General Analyses for Linear Models version 3 plugin (GAMLj3 v3.4.2, https://gamlj.github.io/index.html#general-analyses-for-the-linear-model-in-jamovi). All descriptive statistics are presented as mean ± standard deviation, and statistical test results are reported as the 95% confidence interval (CI) of the differences between testing blocks and BL for that factor, and between males and females for that factor. Although a linear-mixed effects model does not generate effect sizes for the model itself, for reference, we have added effect sizes (ηp²) from a corrected one-way, repeated measures analysis of variance (ANOVA) associated with each linear mixed-effects model.

## Results

### Subjective responses

**Fig 2:**
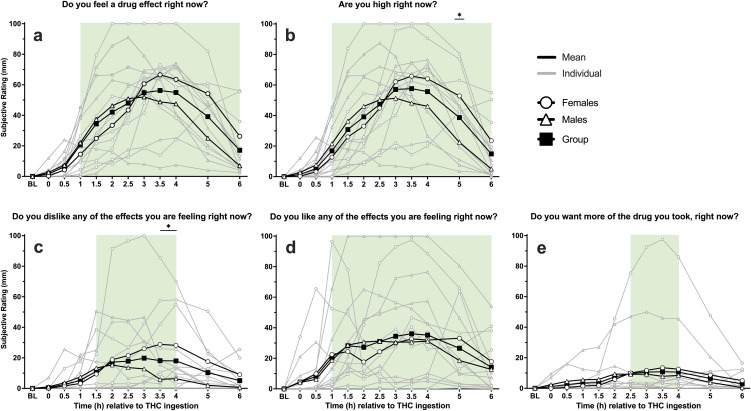
Subjective intoxication to 10 mg of THC. Self-reported responses to the five prompts of the Drug Effect Questionnaire (DEQ) were quantified using 100-mm visual analog scales, anchored by 0 (“Not at all”) and 100 (“Extremely”). Grey symbols depict data from individual participants, whereas black symbols indicate group means. Circles represent females (n = 8), triangles represent males (n = 7), and black squares represent the means for all participants (n = 15). Green regions indicate testing blocks greater than baseline (BL), whereas asterisks (*) indicate values which were greater for females than males.

After ingestion of THC, responses to all DEQ prompts were elevated compared to BL at multiple timepoints (Fig 2). Using the 0-100 mm visual analog scale, participants reported significant subjective drug effects (Fig 2a) beginning at 1 h post-ingestion (95% CI [6.1, 30.5 mm]) which persisted at 6 h (95% CI [4.4, 28.8 mm]; ηp² = 0.657). Similarly, subjective high (ηp² = 0.655; Fig 2b) was increased starting 1 h after ingestion (95% CI [4.9, 29.5 mm]) and remained elevated at 6 h (95% CI [2.0, 26.5 mm]). A significant dislike of subjective drug effects (ηp² = 0.239; Fig 2c) was observed 1.5-4 h post-ingestion (95% CI [1.2, 21.5 mm] at 1.5 h and [7.1, 27.5 mm] at 4 h). Participants reported liking feelings associated with the drug effect starting at 1 h (95% CI [6.7, 34.5 mm]; Fig 2d), and this effect was still present at 6 h (95% CI [1.4, 29.2 mm]; ηp² = 0.274). The desire for more of the drug (Fig 2e) was only elevated between 2.5-4 h (95% CI [1.4, 17.8 mm] at 2.5 h and [2.3, 16.7 mm] at 4 h; ηp² = 0.121). None of the responses to the questions had a main effect of sex. However, there were instances of a sex × time interaction, with females feeling higher than males at 5 h (95% CI [5.9, 55.0 mm]; Fig 2b), and disliking the drug effect more (Fig 2c) at 3.5 and 4 h (95% CI [2.5, 43.2 mm] and [1.5, 42.2 mm], respectively). Complete results of the linear mixed-effects model for DEQ data can be found in the S1 Table.

### Cardiovascular responses

**Fig 3:**
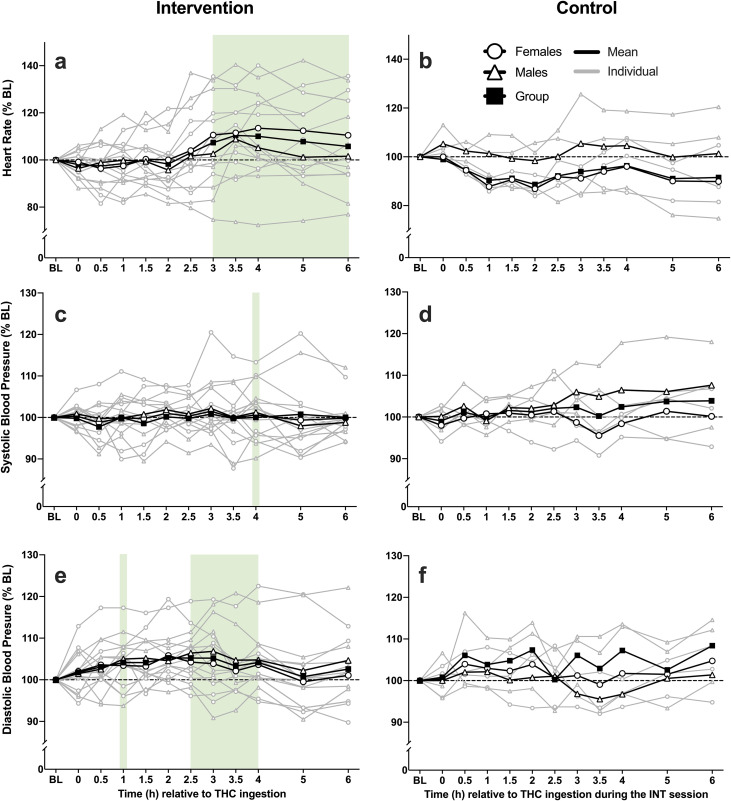
Cardiovascular responses during the Intervention (INT; left panels) and Control (CON; right panels) sessions. Grey symbols depict data from individual participants, whereas black symbols indicate group means. Circles represent females (INT n = 8, CON n = 3), triangles represent males (INT n = 7, CON n = 3), and black squares represent the means for all participants (INT n = 15, CON n = 6). Green regions indicate testing blocks greater than baseline (BL) for the INT session. NB: CON data were included as a covariate in the linear mixed-effects model for the INT data, but a statistical analysis was not performed for the CON data.

During the INT session, none of HR, systolic BP or diastolic BP had a sex × time interaction or main effect of sex. However, there was a main effect of time for all cardiovascular measures (HR ηp^2^ = 0.358; systolic BP ηp^2^ = 0.364; diastolic BP ηp^2^ = 0.484). Compared to BL, HR was elevated at 3 h (95% CI [2.5, 20.0 bpm]) and remained elevated by 6 h (95% CI [0.1, 18.8 bpm]; Fig 3a). Systolic BP was elevated at 4 h compared to BL (95% CI [1.2, 10.4 mmHg]; Fig 3c), whereas diastolic BP was elevated at 1 h (95% CI [0.0, 5.4 mmHg]; Fig 3e) and 2.5-4 h (95% CI [1.0, 6.3 mmHg] at 2.5 h and [1.5, 6.8 mmHg] at 4 h). Complete results of the linear mixed-effects model for cardiovascular data can be found in the S2 Table.

### Cognitive-motor tasks

**Fig 4:**
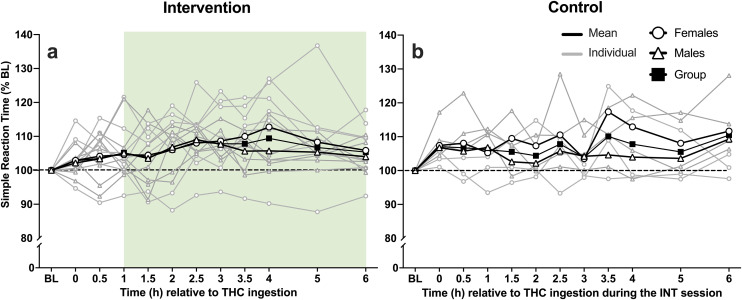
Simple reaction time (SRT) during the Intervention (INT; a) and Control (CON; b) sessions. Grey symbols depict data from individual participants, whereas black symbols indicate group means. Circles represent females (INT n = 8, CON n = 3), triangles represent males (INT n = 7, CON n = 4), and black squares represent the means for all participants (INT n = 15, CON n = 7). Green regions indicate testing blocks greater than baseline (BL) for the INT session. NB: CON data were included as a covariate in the linear mixed-effects model for the INT data, but a statistical analysis was not performed for the CON data.

Full results of the linear mixed-effects model for cognitive-motor task outcomes can be found in the S3 Table. After THC ingestion, SRT did not have a sex × time interaction or main effect of sex; however, there was a main effect of time (ηp² = 0.306; Fig 4a), with RT slower than BL from 1-6 h (95% CI [6.1, 41.0 ms] at 1 h and [8.1, 46.1 ms] at 6 h). There were no statistically significant outcomes for intraindividual variability of SRT.

**Fig 5:**
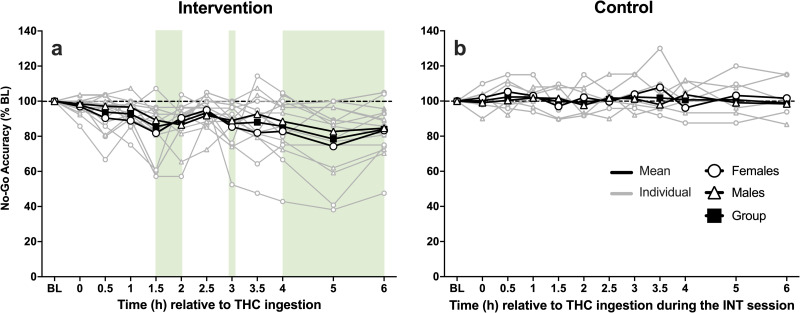
Accuracy of “no-go” trials during the Intervention (INT; a) and Control (CON; b) sessions. Grey symbols depict data from individual participants, whereas black symbols indicate group means. Circles represent females (INT n = 8, CON n = 3), triangles represent males (INT n = 7, CON n = 4), and black squares represent the means for all participants (INT n = 15, CON n = 7). Green regions indicate testing blocks lower than baseline (BL) for the INT session. NB: CON data were included as a covariate in the linear mixed-effects model for the INT data, but a statistical analysis was not performed for the CON data.

In contrast to the SRT data, RT of the “go” trials of the GNG task was influenced by THC only at the last time point (95% CI [−55.0, −1.8 ms]). The intraindividual variability had a main effect of time, with values greater from 3-4 h compared to BL (95% CI [2.9, 23.4 ms] at 3 h and [2.4, 22.9 ms] at 4 h). The accuracy of the “no-go” trials had main effects of time (ηp² = 0.366) and sex (ηp² = 0.485; Fig 5a). After THC ingestion, accuracy was lower than BL at 1.5, 2, 3, 4, 5, and 6 h (95% CI [−26%, −5%] at 1.5 h and [−25%, −3%] at 6 h; Fig 5a). Additionally, females demonstrated lower accuracy than males (95% CI [−24%, −2%]; Fig 5a).

**Fig 6:**
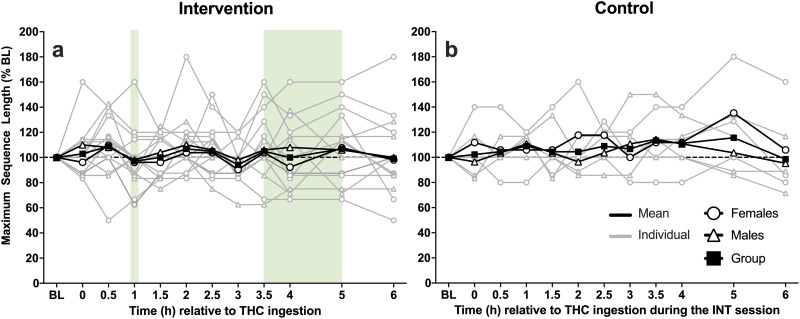
Corsi block tapping test maximum sequence length during the Intervention (INT; a) and Control (CON; b) sessions. Grey symbols depict data from individual participants, whereas black symbols indicate group means. Circles represent females (INT n = 8, CON n = 3), triangles represent males (INT n = 7, CON n = 4), and black squares represent the means for all participants (INT n = 15, CON n = 7). Green regions indicate testing blocks lower than baseline (BL) for the INT session. NB: CON data were included as a covariate in the linear mixed-effects model for the INT data, but a statistical analysis was not performed for the CON data.

For the CBT task, which measured short-term memory, THC ingestion led to a main effect of time (ηp² = 0.147), whereby the maximum sequence length was reduced compared to BL at 1 h (95% CI [−2.4, −0.0]; Fig 6a) and from 3.5-5 h (95% CI [−2.4, −0.0] at 3.5 h and [−2.6, −0.3] at 5 h). No other differences were found.

### Correlation between subjective and objective results

Considering the strong effect of THC on SRT, we opted to run a post-hoc linear mixed-effects model, with SRT as the dependent variable, timepoint and sex as fixed-effect factors, and the responses to the first DEQ prompt (“Do you feel a drug effect right now?”) as a covariate to determine if there was a significant association between the objective SRT results and subjective intoxication. The results of this test demonstrate that SRT and DEQ responses were significantly correlated, with a single unit increase for the DEQ response associated with an increase of 0.2 ms for SRT. Additionally, when DEQ responses were included as a covariate, SRT was significantly slower than BL only at 2.5 h and 4 h. See S4 Table for details.

### Adverse events

Although no major adverse events occurred, two participants reported experiencing anxiety at some point during the INT session. For one participant, the anxiety tracked their subjective intoxication, which aligned with their previous experiences using cannabis. The other participant reported feeling anxious ~1 h following the onset of subjective intoxication. Their anxiety was mitigated when they were able to eat following the 2-h testing block, which was also consistent with this participant’s previous experiences using cannabis.

## Discussion

The goal of the present study was to characterize the progression of subjective and objective intoxication arising from the ingestion of 10 mg of THC via commercially-available THC gel capsules. Both sets of data broadly agree with each other as many indices of intoxication manifested after 1-2 h and remained elevated compared to BL at 5-6 h after ingestion, including robust impairments in SRT and GNG accuracy, which correlated with reports of subjective drug effects.

### Subjective responses

The results of the DEQ align broadly with previous work to consider the acute effects of edible THC [6,8,9]. For example, similar levels and timing of subjective drug effects were reported by Vandrey and colleagues following 10 mg edible THC administration [6]. In that study, blood THC had a peak of ~1 ng/mL (range 0-3 ng/mL), alongside 1.3 ng/mL (0-2 ng/mL) of 11-OH-THC, the primary psychoactive metabolite of THC, with both blood markers elevated throughout the entirety of the testing window of the present study; i.e., 1-6 h [6]. However, caution should be taken when directly comparing the results of these studies, as interindividual differences may have influenced measurements. To our knowledge, the present study is the first to use a commercially-available THC product or a statistical approach designed to identify onset and offset times of subjective and objective effects, which allows for a better understanding of the time course of intoxication. As revealed by Fig 2, ingestion of 10 mg of THC provides a broad window of time to explore its acute effects, with subjective ratings of intoxication beginning at ~1 h and lasting until at least 6 h after ingestion (when data collection ceased). Even though all testing blocks between 1-6 h are statistically greater than BL, the ideal testing window appears to be 3-4 h after ingestion, with a considerable perception of feeling high between 2-5 h. These findings support and extend previous work which has examined the time course of intoxication from edible cannabis use [8,31,32], and confirm a later and longer-lasting window of intoxication compared to inhaled cannabis [5,33]. Additionally, the finding that SRT is correlated with responses to the question, “Do you feel a drug effect right now?” is, to the best of our knowledge, the first time that an objective measurement of human behaviour has been shown to correlate with a subjective assessment of cannabis intoxication. Thus, SRT may represent an objective surrogate for subjective perceptions of intoxication arising from edible cannabis use in infrequent users. This result also demonstrates that the subjective perception of intoxication may have specific neural correlates related to voluntary movement initiation, which warrants further study. Due to the nature of our experiment, we cannot identify direct neurophysiological mechanisms responsible for these results. However, it is currently understood that the primary mechanism behind the intoxicating effects of cannabis is driven by THC persistently activating CB1 receptors present within the nervous system [3]. Although this mechanism may account for subjective intoxication, as described in the next section, it may not best explain the motor behavioural outcomes we observed.

### Objective responses

Similar to previous reports [11,12], THC intoxication led to slower SRT. To generate a response to a stimulus, three stages of executive processing are thought to occur: 1) stimulus identification, 2) response selection, and 3) motor programming [28]. As the singular response of an SRT task allows for pre-programming of the nature of the response and the associated motor outputs, performance is thought to reflect the latency of the stimulus identification stage. Thus, the observed slowing of SRT after THC ingestion is likely to reflect an impaired ability to properly identify a stimulus. This explanation may also provide insight into why there was poorer accuracy for “no-go” trials of the GNG task when participants became intoxicated; i.e., as their ability to properly distinguish between a “go” vs. “no-go” stimulus worsened, more errors were made. Considering that the GNG task was constituted predominantly of “go” trials, the tendency to erroneously respond more often on the “no-go” trials while intoxicated may reflect a strategy to maximize the number of correct responses in the context of noisy sensory feedback, which would limit the ability to distinguish between stimuli. This perspective is bolstered by the work of [34], which indicated that certain dopaminergic systems in the central nervous system, including those which facilitate the generation of movements in response to specific stimuli (the D1 system), are overstimulated in conjunction with cannabinoid receptor activation. This mechanism would likely create noise within the system and ultimately lead to worse performance on tasks that require stimulus-action coupling [35]. Similarly, overstimulation of the D1 system during acute cannabis intoxication may also explain the failure to improve CBT performance after THC ingestion; i.e., one needs to link the visuospatial memory of the order in which stimuli were presented and produce an action that replicates the sequence. Indeed, the D1 system has been shown to be important for the functioning of working memory [36]. Further, [37] identified that acute and chronic cannabis use altered function from the retina to the visual cortex, which suggests that the processing, integration, and action coupling of visual information may be impaired by THC consumption.

For our cardiovascular responses, HR was elevated starting at 3 h and remained significantly higher than BL when testing finished 6 h after ingesting the THC. The effects of THC on BP were less pronounced as systolic BP was only greater than BL at 4 h, whereas diastolic was elevated at 1 h and from 2.5-4 h. The HR data are consistent with previous findings in response to inhaled THC [5]; however, the observed increases for BP conflict with the findings of Zamarripa and colleagues [9], who reported no effect of 20 mg of ingested THC. The primary mechanism through which THC is suspected to impact cardiac activity is via increased cardiac adrenergic receptor stimulation and vagal withdrawal [38], shifting the cardiac autonomic balance to that of greater sympathetic influence and, thus, elevating HR. The influence of THC on BP is less clear and, while altered sympathetic nervous system activity is often reported, current literature is conflicted on the direction of this change [39–41].

### Ecological validity of dosage and setting

To appropriately address the primary goal of establishing a time course of edible THC intoxication, ecological validity was main a focus of the current study design. Although hindered by the laboratory setting, the other elements of the experiment were chosen to align with this focus. These elements included using an edible THC product that is readily available for purchase, allowing participants to engage in a wide variety of tasks in between testing blocks, and, after an initial wait of 2 h, allowing them to consume food and beverages ad libitum. A standardized THC dosage of 10 mg was chosen for two reasons: first, this dose produces significant intoxication without frequently eliciting adverse events that occur at higher doses [5] and, second, 10 mg of THC is the highest dose legally available for purchase in a single package of edible cannabis in Canada [38].

### Importance of results for researchers, clinicians, policy-makers, and the general public

In addition to documenting the acute effects of THC on various subjective and objective indices of THC intoxication, this study was designed to establish an appropriate testing window for future research with edibles. However, researchers are not the only group to benefit from these data, as this information is usable by anyone who has a stake in knowing the time course of intoxication from edible THC consumption. Such parties include: 1) clinicians who prescribe cannabis for medicinal purposes, and the patients who will use it, 2) employers who wish to take a data-driven approach to a cannabis policy for their organizations, 3) members of the general public who use cannabis recreationally, and would like to know what symptoms they can expect after consuming a common dosage of edible THC, and 4) policy makers, e.g., those who decide on recreational cannabis product packaging requirements such as the 10 mg THC limit on edible products.

### Considerations

Participants were not blinded to the nature of their intervention in the present study. There are some indications that the expectancy of THC consumption may impact outcomes, and so the results reported here may have been influenced by knowledge of the nature of the intervention [42]. Concerning sex-based differences, although some were identified, we did not specifically ensure that this study was powered for such comparisons, potentially inflating the risk of type II error for these outcomes. Nor did we standardize testing relative to the phase of the menstrual cycle, which could have increased the variability within the female dataset. Of note, the females of the present sample reported greater cannabis use than the males (Table 1). Thus, although still classified by our operational definition as infrequent users, females may have been more habituated to the effects of intoxication, and thus demonstrated lower magnitudes of impairment than the males.

Additionally, rather than administer a standardized 10 mg of THC, an alternative approach could have been to scale the dosage to anthropometric data (e.g., body mass). However, given an inability to control for interindividual variability in cannabis metabolism and tolerance independent of body size, we decided that providing all participants with a common dosage of a commercially-available cannabis product aided our pursuit of ecological validity. Further, post-hoc linear mixed-effects models were conducted to examine the relationship of body mass with both DEQ1 (“Do you feel a drug effect right now?”) responses and SRT performance. Neither test produced significant results, suggesting, for this sample, there was no correlation between body size and subjective or objective markers of intoxication (S5 Table). The lack of a standardized pre-testing session fasting period and individualized food consumption 2 h after THC administration may have also increased the variability in THC absorption rate [27]. We opted to have participants consume their standard breakfast and to not control food intake after the mandatory 2-h fasting period following THC administration to increase the ecological validity of the study, as we deemed it unlikely that the average person would fast for a prolonged period or significantly alter their diet prior to or after recreational or medicinal cannabis consumption. In our pursuit of ecological validity, allowing participants to eat ad libitum after the 2-h time point likely induced some variability in the cardiovascular data, as post-prandial changes in HR and BP are well documented [43]. However, as increased appetite is a common symptom of acute cannabis intoxication [44], we wanted to maintain participant comfort by allowing them to eat as desired. Lastly, the control group included only approximately half the sample of the intervention group, which reduced the power of statistical tests for those outcomes that incorporated the control dataset into the models (i.e., the cognitive-motor and cardiovascular outcomes). With a complete control dataset, more differences may have been detected.

### Future Directions

As body mass did not correlate with subjective ratings of intoxication, the shapes of the curves in Figs 2a-c, as well as some sex × time interactions, lead us to conclude that sex-based differences may exist for the subjective experience of THC intoxication. Specifically, males appear to experience peak intoxication earlier than females, whereas females appear to have higher peak values. Notably, although the perception of feeling high was greater for females than males [25], only one of the cognitive-motor outcomes demonstrated a sex-based difference; GNG accuracy. Others have suggested that sex-based differences may exist for subjective drug effects and cognitive-motor task performance arising from chronic cannabis use [45,46]. Moreover, preclinical work indicates there may be a sex-based difference for cannabinoid receptor expression, where females demonstrate lower CB1 receptor density in most brain regions, but higher receptor activity [47]. However, evidence that biological sex affects cognitive-motor performance during acute cannabis intoxication in humans remains limited [48], and future work should explore possible sex-based differences for acute subjective and objective effects of cannabis intoxication.

Though THC is the primary psychoactive component of cannabis, the overwhelming majority (~93%) of cannabis consumed in Canada [4], and likely around the world, contains other cannabinoids, such as cannabidiol (CBD). Many of these cannabinoids are postulated to interact directly with the human motor system. Specifically, CBD is thought to alter THC binding and metabolism when they are co-consumed [19,49]. Thus, CBD should be investigated for the effect it has on motor behaviour by itself and when co-consumed with THC.

## Conclusion

To conclude, after ingesting softgel capsules containing 10 mg of THC, participants experienced significant subjective and objective effects of intoxication, even when controlling for learning effects and natural daily variation of cognitive-motor task performance. In most instances, the effects of cannabis intoxication appeared ~1 h after ingestion, peaked at ~3-4 h, and were still present during the last testing block (6 h). It is our recommendation that future studies intending to assess the acute influence of cannabis edibles on human behaviour should conduct testing 2-5 h post-intervention to align with the greatest levels of intoxication.

## Supporting information

S1 Table Linear mixed-effect model results for responses to the Drug Effects Questionnaire (DEQ). (DOCX)

S2 Table Linear mixed-effect model results for cardiovascular measures. (DOCX)

S3 Table Linear mixed-effect model results for cognitive-motor measures. (DOCX)

S4 Table Linear mixed-effect model results for simple reaction time using subjective drug effects as a covariate. (DOCX)

S5 Table Linear mixed-effect model results for body mass correlations with simple reaction time and Drug Effect Questionnaire prompt 1 (DEQ 1). (DOCX)
