# Supplementary material for: A six-hour time series of the acute effects of ingested cannabis intoxication on a battery of cognitive-motor tasks
Source: PLoS One. 2026 Aug 13;21(8):e0353389. doi: 10.1371/journal.pone.0353389 (PMC13472365; doi:10.1371/journal.pone.0353389)
Supplement: Supplementary file 1 [file pone.0353389.s006.pdf]

PLOS ONE

A six-hour time series of the acute effects of ingested cannabis intoxication on a battery of cognitive-motor tasks

--Manuscript Draft--

**Manuscript Number:**	PONE-D-25-56417
**Article Type:**	Research Article
**Full Title:**	A six-hour time series of the acute effects of ingested cannabis intoxication on a battery of cognitive-motor tasks
**Short Title:**	Time series of cannabis intoxication
**Corresponding Author:**	Chris McNeil<br>The University of British Columbia Okanagan<br>Kelowna, BC CANADA
**Keywords:**	cannabinoids; tetrahydrocannabinol; reaction time; Heart rate; perceived drug effects
**Abstract:**	The psychoactive component of cannabis, Δ9-tetrahydrocannabinol (THC), is known to alter cognitive-motor function; however, despite the high prevalence of cannabis use globally, the time course of the effects of ingesting THC is unclear. To allow future studies to accurately time their assessment of the impacts of cannabis on behaviour, this study aimed to establish a detailed time series of subjective intoxication and cognitive-motor performance following THC ingestion. Fifteen infrequent cannabis users (8 females; 29.4 ± 8.1 years) completed a testing battery before (baseline) and at 11 timepoints (0-6h) after ingesting two 5-mg THC capsules. Subjective drug effects and cardiovascular measures were recorded, and cognitive-motor function was measured via performance on simple reaction time, go/no-go reaction time, and Corsi block tapping tasks. Linear mixed-effects models were used to determine if subjective and objective measures changed during the time series. Compared to baseline, participants reported significant subjective drug effects 1-6h post-ingestion. Heart rate was elevated from 3-6h, whereas blood pressure was elevated at timepoints between 1-4h. Individuals demonstrated a robust increase of simple reaction time from 1-6h, reduced go/no-go accuracy between 1.5-6h, and impaired Corsi blocking tapping performance between 1-5h. Overall, the ideal window to test the cognitive-motor effects of cannabis intoxication appears at 3-4h post-THC ingestion, with a marked perception of feeling high between 2-5h.
**Order of Authors:**	Paige Victoria Copeland
	Quinn Malone
	Brian Harry Dalton
	Chris Jeremy McNeil
**Additional Information:**	
**Question**	**Response**
**Financial Disclosure**<br><br>Enter a financial disclosure statement that describes the sources of funding for the work included in this submission. Review the submission guidelines for detailed requirements. View published research articles from *PLOS ONE* for specific examples.<br><br><br>This statement is required for submission	Yes

Powered by Editorial Manager® and ProduXion Manager® from Aries Systems Corporation

and **will appear in the published article** if the submission is accepted. Please make sure it is accurate.<br><br>**Funded studies**<br>Enter a statement with the following details:<br>• Initials of the authors who received each award<br>• Grant numbers awarded to each author<br>• The full name of each funder<br>• URL of each funder website<br>• Did the sponsors or funders play any role in the study design, data collection and analysis, decision to publish, or preparation of the manuscript?<br><br>Did you receive funding for this work?	
Please add funding details.<br>as follow-up to "**Financial Disclosure**<br><br>Enter a financial disclosure statement that describes the sources of funding for the work included in this submission. Review the submission guidelines for detailed requirements. View published research articles from *PLOS ONE* for specific examples.<br><br>This statement is required for submission and **will appear in the published article** if the submission is accepted. Please make sure it is accurate.<br><br>**Funded studies**<br>Enter a statement with the following details:<br>• Initials of the authors who received each award<br>• Grant numbers awarded to each author<br>• The full name of each funder<br>• URL of each funder website<br>• Did the sponsors or funders play any role in the study design, data collection and analysis, decision to publish, or preparation of the manuscript?<br><br>Did you receive funding for this work?"	This work was supported by the Natural Sciences and Engineering Research Council of Canada under DG grant 5018423-2020 (C.J.M) and the Stober Foundation Health Fund (C.J.M. & B.H.D.). The funding bodies had no decision-making role in the study design, data collection or analysis, decision to publish, or preparation of the manuscript.

Please select the country of your main research funder (please select carefully as in some cases this is used in fee calculation).<br>as follow-up to "**Financial Disclosure**<br><br>Enter a financial disclosure statement that describes the sources of funding for the work included in this submission. Review the submission guidelines for detailed requirements. View published research articles from *PLOS ONE* for specific examples.<br><br>This statement is required for submission and **will appear in the published article** if the submission is accepted. Please make sure it is accurate.<br><br>**Funded studies**<br>Enter a statement with the following details:<br>• Initials of the authors who received each award<br>• Grant numbers awarded to each author<br>• The full name of each funder<br>• URL of each funder website<br>• Did the sponsors or funders play any role in the study design, data collection and analysis, decision to publish, or preparation of the manuscript?<br><br>Did you receive funding for this work?"	CANADA - CA
**Competing Interests**<br><br>Use the instructions below to enter a competing interest statement for this submission. On behalf of all authors, disclose any competing interests that could be perceived to bias this work—acknowledging all financial support and any other relevant financial or non-financial competing interests.<br><br>This statement is required for submission and **will appear in the published article** if the submission is accepted. Please make sure it is accurate and that any funding	The authors declare there are no competing interests, financial or otherwise.

Powered by Editorial Manager® and ProduXion Manager® from Aries Systems Corporation

sources listed in your Funding Information later in the submission form are also declared in your Financial Disclosure statement.<br><br>View published research articles from *PLOS ONE* for specific examples.<br><br>**NO authors have competing interests**<br><br>Enter: *The authors have declared that no competing interests exist.*<br><br>**Authors with competing interests**<br><br>Enter competing interest details beginning with this statement:<br><br>*I have read the journal's policy and the authors of this manuscript have the following competing interests: [insert competing interests here]*<br><br>* typeset	
**Ethics Statement**<br><br>Enter an ethics statement for this submission. This statement is required if the study involved:<br><br>• Human participants<br>• Human specimens or tissue<br>• Vertebrate animals or cephalopods<br>• Vertebrate embryos or tissues<br>• Field research<br><br>Write "N/A" if the submission does not require an ethics statement.<br><br>General guidance is provided below. Consult the submission guidelines for detailed instructions. **Make sure that all information entered here is included in the Methods section of the manuscript.**	The study was approved by the Clinical Research Ethics Board at the University of British Columbia (H23-00249) and conducted in accordance with Canada's Tri-Council Policy Statement: Ethical Conduct for Research involving Humans and the Declaration of Helsinki. Additionally, this research was approved by Health Canada and licensed under the Canadian Cannabis Act and Cannabis Regulations (LIC-4FN4K9EH76-2024). Written and oral informed consent was obtained from the participants prior to the onset of the study.

**Format for specific study types**<br><br>**Human Subject Research (involving human participants and/or tissue)**<br>• Give the name of the institutional review board or ethics committee that approved the study<br>• Include the approval number and/or a statement indicating approval of this research<br>• Indicate the form of consent obtained (written/oral) or the reason that consent was not obtained (e.g. the data were analyzed anonymously)<br><br>**Animal Research (involving vertebrate animals, embryos or tissues)**<br>• Provide the name of the Institutional Animal Care and Use Committee (IACUC) or other relevant ethics board that reviewed the study protocol, and indicate whether they approved this research or granted a formal waiver of ethical approval<br>• Include an approval number if one was obtained<br>• If the study involved *non-human primates*, add *additional details* about animal welfare and steps taken to ameliorate suffering<br>• If anesthesia, euthanasia, or any kind of animal sacrifice is part of the study, include briefly which substances and/or methods were applied<br><br>**Field Research**<br><br>Include the following details if this study involves the collection of plant, animal, or other materials from a natural setting:<br>• Field permit number<br>• Name of the institution or relevant body that granted permission	
**Data Availability**<br><br>Authors are required to make all data underlying the findings described fully available, without restriction, and from the time of publication. PLOS allows rare exceptions to address legal and ethical concerns. See the PLOS Data Policy and FAQ for detailed information.	Yes - all data are fully available without restriction

A Data Availability Statement describing where the data can be found is required at submission. Your answers to this question constitute the Data Availability Statement and **will be published in the article**, if accepted.<br><br>**Important:** Stating 'data available on request from the author' is not sufficient. If your data are only available upon request, select 'No' for the first question and explain your exceptional situation in the text box.<br><br>Do the authors confirm that all data underlying the findings described in their manuscript are fully available without restriction?	
**Describe where the data may be found in full sentences. If you are copying our sample text, replace any instances of XXX with the appropriate details.**<br><br>• If the data are **held or will be held in a public repository**, include URLs, accession numbers or DOIs. If this information will only be available after acceptance, indicate this by ticking the box below. For example: *All XXX files are available from the XXX database (accession number(s) XXX, XXX.).*<br>• If the data are all contained **within the manuscript and/or Supporting Information files**, enter the following: *All relevant data are within the manuscript and its Supporting Information files.*<br>• If neither of these applies but you are able to provide **details of access elsewhere**, with or without limitations, please do so. For example:<br><br>*Data cannot be shared publicly because of [XXX]. Data are available from the XXX Institutional Data Access / Ethics Committee (contact via XXX) for researchers who meet the criteria for access to confidential data.*<br><br>*The data underlying the results presented in the study are available from (include the name of the third party*	All relevant data are within the manuscript and its Supporting Information files.

and contact information or URL).<br>• This text is appropriate if the data are owned by a third party and authors do not have permission to share the data.<br><br>* typeset	
Additional data availability information:	Tick here if the URLs/accession numbers/DOIs will be available only after acceptance of the manuscript for publication so that we can ensure their inclusion before publication.

Powered by Editorial Manager® and ProduXion Manager® from Aries Systems Corporation

Manuscript Click here to access/download;Manuscript;Copeland et al 2025.docx

A six-hour time series of the acute effects of ingested cannabis intoxication on a battery of cognitive-motor tasks

Paige V. Copeland, Quinn Malone, Brian H. Dalton, Chris J. McNeil

School of Health and Exercise Sciences, The University of British Columbia Okanagan

Short title: Time series of cannabis intoxication

Corresponding author:
Chris J McNeil, PhD
Faculty of Health and Social Development | School of Health and Exercise Sciences
The University of British Columbia | Okanagan Campus
1147 Research Road | Kelowna BC | V1V 1V7 Canada
Phone 250-807-9664
chris.mcneil@ubc.ca

Abstract

The psychoactive component of cannabis, Δ9-tetrahydrocannabinol (THC), is known to alter cognitive-motor function; however, despite the high prevalence of cannabis use globally, the time course of the effects of ingesting THC is unclear. To allow future studies to accurately time their assessment of the impacts of cannabis on behaviour, this study aimed to establish a detailed time series of subjective intoxication and cognitive-motor performance following THC ingestion. Fifteen infrequent cannabis users (8 females; 29.4 ± 8.1 years) completed a testing battery before (baseline) and at 11 timepoints (0-6h) after ingesting two 5-mg THC capsules. Subjective drug effects and cardiovascular measures were recorded, and cognitive-motor function was measured via performance on simple reaction time, go/no-go reaction time, and Corsi block tapping tasks. Linear mixed-effects models were used to determine if subjective and objective measures changed during the time series. Compared to baseline, participants reported significant subjective drug effects 1-6h post-ingestion. Heart rate was elevated from 3-6h, whereas blood pressure was elevated at timepoints between 1-4h. Individuals demonstrated a robust increase of simple reaction time from 1-6h, reduced go/no-go accuracy between 1.5-6h, and impaired Corsi blocking tapping performance between 1-5h. Overall, the ideal window to test the cognitive-motor effects of cannabis intoxication appears at 3-4h post-THC ingestion, with a marked perception of feeling high between 2-5h.

Introduction

Although cannabis is believed to have been used as a medicine for nearly 5000 years [1], criminalization of the drug in the 20th century greatly curtailed scientific evaluation, leaving much unknown about the acute and chronic effects of cannabis on human function. As recreational cannabis use continues to be legalized across the world, it has become increasingly important to advance our knowledge about the effects of intoxication arising from the consumption of the psychoactive component of cannabis, Δ9-tetrahydrocannabinol (THC) [2,3]. Of the few studies to systematically investigate the acute effects of cannabis, most had participants smoke the drug. Although inhalation remains the most common form of usage in Canada [4], ingested cannabis (colloquially referred to as 'edibles') is increasingly popular, as 55% of cannabis users report consuming edibles [4], and numerous options (e.g., drinks, gummies, capsules, etc.) are commercially available. As inhalation and ingestion result in different pharmacokinetic and pharmacodynamic outcomes for the psychoactive components of cannabis [5–7], there is a need to understand the acute effects of ingested THC. However, there is limited information about the effects of this mode of consumption on cognitive-motor function [6,8,9].

Although firm conclusions concerning cognitive-motor performance during acute cannabis intoxication are difficult to draw due to the heterogeneity of previous study designs, some results appear robust. For example, though null effects have been reported with ingested THC [6,8], simple reaction time (SRT) is impaired after inhalation [10,11]. Similarly, inhaling cannabis increases the number of response errors during "no-go" trials of a go/no-go (GNG) task [12,13]. Short-term memory is also impaired acutely by cannabis inhalation [10,14,15]. However, to the best of our knowledge, no study has evaluated how cognitive-motor

performance is affected by ingestion of a commercially-available edible THC product, taken measurements more than once per hour, nor considered possible effects of biological sex.

Compared to inhalation, edibles are an appealing method of cannabis consumption to use in research because; THC and other cannabinoid dosages can be highly controlled, there are fewer perceived health risks [4], and there is a prolonged period of intoxication [6,8,9]. To aid future research into the acute effects of commercially-available edible THC products, a time course (i.e., onset and offset) of indices of intoxication should be established. Thus, the aim of this study was to evaluate numerous subjective and objective measures for six hours following ingestion of 10mg of THC. We hypothesised that effects of intoxication would be evident after ~1h and last until ~4h after ingestion.

Methods

Participants

The study was approved by the Clinical Research Ethics Board at the University of British Columbia (H23-00249) and conducted between March 7 and July 19, 2024 in accordance with Canada's Tri-Council Policy Statement: Ethical Conduct for Research involving Humans and the Declaration of Helsinki. Additionally, this research was approved by Health Canada and licensed under the Canadian Cannabis Act and Cannabis Regulations (LIC-4FN4K9EH76-2024). Fifteen healthy individuals (29±8y, 174±11cm, 72±18kg; 8 females; see Table 1 for full demographic data) participated in the intervention session (INT). Seven of those participants (32±9y, 171±10cm, 65±11kg; 3 females) also completed an additional control session (CON), during which no intervention was administered. Accordingly, participants were not blinded to the nature of the intervention during either session. To limit the influence of chronic cannabis use on the

effects of acute intoxication [16], participants were recruited from a population characterized as infrequent users, which was defined as not having used cannabis approximately more than once per week during the previous six months [8,17]. To reduce to risk of adverse events caused by acute cannabis use (e.g., acute anxiety, paranoia, nausea, hallucinations) [5], individuals who had never consumed THC, and were thus unaware if they may be predisposed to potential adverse effects of cannabis consumption, were excluded from participation. Written and oral informed consent were granted during a familiarization session.

Table 1. Participant characteristics

	**Measure**	**Group (INT n=15, CON n=7)**		**Females (INT n=8, CON n=3)**		**Males (INT n=7, CON n=4)**	
		M ± SD	Range	M ± SD	Range	M ± SD	Range
**Intervention Group**	Age (y)	29.4 ± 8.1	21-47	26.1 ± 4.1	21-32	33.1 ± 8.8	24-47
	Height (cm)	174.3 ± 10.6	157-199	165.6 ± 7.0	157-174	184.1 ±9.8†	171-199
	Body mass (kg)	72.1 ± 18.0	50-113	68.1 ±19.6	57-113	76.6 ± 9.9	61-89
**Control Subgroup**	Age (y)	32 ± 8.8	22-47	27.3 ± 5.0	22-32	35.5 ± 11.2	24-47
	Height (cm)	171.1 ± 9.9	157-183	162.3 ± 9.2	157-173	177.8 ± 6.2	171-183
	Body mass (kg)	65.4 ± 10.6	50-82	57 ± 7	50-64	71.8 ± 10.3	61-82
**DFAQ-CU Variables**	Frequency of cannabis use (d/y)	4.8 ± 5.2	0-15	8.1 ± 5.5	0.5-15	1.1 ± 0.9†	0-3
	Age of cannabis use onset (y)	18.6 ± 3.4	13-25	18.5 ± 4.5	13-25	18.7 ± 2.2	17-22

Values are means ± standard deviation. Abbreviations: INT, intervention session; CON, control session; DFAQ-CU, Daily Sessions, Frequency, Age of Onset, and Quantity of Cannabis Use Inventory. † denotes significant difference from females in the same group ($p<0.001$).

Familiarization, experimental, and control sessions

No more than seven days prior to the first testing session (i.e., INT or CON), participants attended a familiarization session of ~60min. After providing consent, participants completed the Daily Sessions, Frequency, Age of Onset, and Quantity of Cannabis Use Inventory (DFAQ-CU)

to contextualize their personal history with the drug [18]. Next, individuals performed one full block of the testing battery (see below). Of the seven participants who took part in both sessions, four completed the CON session first. Sessions were separated by at least one week to allow for intervention washout [8,19]. The INT and CON sessions were structured similarly (Fig 1), except that no intervention was administered during the CON session.

Fig 1. Study protocol. The bottom of the schematic depicts the timing of testing blocks for subjective and objective measures of intoxication, which are shown in the breakout box (top). The order of heart rate (HR) and blood pressure (BP) measurements, as well as administration of the Drug Effects Questionnaire (DEQ) was consistent among all participants. The cognitive-motor tasks (shaded area), comprised of simple reaction time (SRT), Go/No-Go (GNG) reaction time, and Corsi block tapping (CBT), were performed in a pseudo-randomized order and counterbalanced across participants. The baseline testing block (BL) concluded ~10min before the ingestion of the intervention (10mg THC) at 0h.

Cannabis product

For the INT session, participants ingested two softgel capsules containing 5mg of THC each (Redecan 5:0 Gems^{TM}, Tilray Brands Inc., New York City, USA). We chose 10mg of THC because it is considered a low-to-moderate dosage [20,21], and it appears to be the highest tested standardized dose that is unlikely to lead to adverse events, such as acute anxiety, paranoia, or emesis[5]. Further, in Canada, 10mg is the maximal amount of THC that can be sold in a single package of a concentrated cannabis product (e.g., softgel capsules, gummies, etc.). Through pilot testing, the 10mg dose of THC was established to be sufficient to induce common symptoms of acute cannabis intoxication (i.e., altered mood and perception).

Study design

Testing sessions were started in the morning (9:00-11:30AM) and ended in the afternoon (4:00-6:30PM). During the INT and CON sessions, 12 testing blocks were conducted (Fig 1), each

consisting of a set experimental battery. Ten minutes after completion of the first testing block (baseline; BL), at 0h, participants consumed the THC capsules (INT) or nothing (CON). Immediately afterward, the 0h testing block began, with subsequent blocks starting 0.5, 1, 1.5, 2 2.5, 3, 3.5, 4, 5, and 6h later.

Each testing block took 15-20min to complete, and began with a measurement of heart rate (HR) and blood pressure (BP), followed by a subjective rating of drug effects via the Drug Effects Questionnaire (DEQ) [22]. Next, participants completed a battery of three cognitive tasks, with the order pseudo-randomized and counterbalanced across participants, but fixed for each participant for all testing blocks during both INT and CON sessions. To conclude the testing block, participants gave another subjective rating of drug effects and again had HR and BP measured. During the 10-15min breaks between testing blocks, participants were instructed to refrain from activities that could lead to increased levels of mental or emotional arousal (e.g., working on a personal computer, watching emotionally-stimulating media). Participants mainly opted to engage in sedentary activities (e.g., conversing with the investigators, watching emotionally-neutral documentaries), though some partook in supervised walks in and around the building containing the testing room. To limit interindividual differences of gastrointestinal absorption of THC due to food consumption [23], participants were prohibited from eating or drinking anything but water from the start of BL until the conclusion of the testing block at 2h. During subsequent breaks, participants were allowed to eat and drink ad libitum. All but one participant opted to eat during the breaks between 2h and 3h.

Subjective measures

To quantify subjective intoxication, participants completed a five-item version of the DEQ [22], which included the following prompts: “Do you feel a drug effect right now?”; “Are you high

right now?"; "Do you dislike any of the effects you are feeling right now?"; "Do you like any of the effects you are feeling right now?"; and "Would you like more of the drug you took, right now?". For each prompt, participants were instructed to draw a single vertical line along a 100mm visual analog scale that ranged from 0 (Not at all) to 100 (Extremely). For each DEQ item, statistical analysis was conducted using the mean value of the two ratings obtained within each testing block.

Cardiovascular measurements

An automated upper-arm blood pressure monitor (BP7450CAN, OMRON Healthcare Co., Ltd., Kyoto, Japan) was used to record HR as well as systolic and diastolic BP. As with the DEQ items, statistical analyses of HR and BP data were conducted using the mean of the values recorded at the start and end of each testing block. Due to technical limitation of the automated blood pressure cuff (which was unable to measure HR or BP for individuals with a HR below 40bpm), data from one individual were unable to be sampled during their control session; therefore, the control cardiovascular dataset has a sample of six participants.

Cognitive tasks

During each testing block, participants sat at a desk to complete the following three cognitive tasks: SRT, GNG, and Corsi block tapping (CBT). Using Presentation software (v23.1, Neurobehavioral Systems Inc., Berkeley, USA), the first three tasks were completed on a laptop computer positioned ~60cm in front of the participant's face. A wireless mouse held in the right hand was used for each task.

The SRT task measured the speed required to perceive and trigger a response to a stimulus [24], which was a red square appearing in the centre of the screen. The task consisted of

45 visual stimuli presented with interstimulus intervals of 2000-3000ms. Participants were instructed to respond as quickly as possible to the stimulus by clicking the left mouse button, after which the stimulus disappeared. If participants did not respond within 500ms, the stimulus would disappear and the next interstimulus interval would begin. Responses made >1000ms after stimulus onset was not considered a valid reaction time (RT), nor was a response made within 100ms of stimulus onset, which was considered to be anticipatory. Invalid RTs were excluded from analysis. Only a small proportion of trials were rejected for these reasons (0.30% and 0.43% of INT and CON datasets, respectively). From the SRT task, a participant's mean RT and intraindividual RT variability were calculated each testing block.

The GNG task examined inhibitory control [24]. It consisted of 96 "go" and 48 "no-go" stimuli that were presented with an interstimulus interval of 700ms. The "go" stimuli were blue, green, or red squares (32 of each), to which the participants were instructed to respond as quickly as possible by clicking the left button on the mouse, after which the stimulus disappeared. The "no-go" stimulus consisted of a black square, to which the participants were instructed to not respond and to wait until it disappeared from the screen. All stimuli to which participants did not respond were displayed for 1000ms before disappearing. For each testing block, mean RT and intraindividual RT variability were derived for the "go" stimuli. Accuracy for "no-go" stimuli was expressed as the percent of "no-go" stimuli correctly avoided.

The CBT test examined short term memory [25]. It involved the presentation of nine squares on screen, which lit up in a pseudorandom sequence each trial. After the sequence finished, participants were instructed to replicate the order by clicking each square using the left mouse button. The sequence length began at three squares, with participants presented three unique trials. If participants were successful at reproducing two of the three sequences, the

sequence length would increase by one, and three new trials would be presented. The test ended when a participant failed to replicate two sequences at a given level. The highest level achieved (i.e., maximum remembered span) was the outcome measure.

Statistical analysis

To test for differences of subjective intoxication, cardiovascular measures, and cognitive task performance over time, linear mixed-effects models were used. The models included the outcome measure as the dependent variable, as well as testing block (BL, 0, 0.5, 1, 1.5, 2, 2.5, 3, 3.5, 4, 5, 6h) and sex (female or male; F or M, respectively) as fixed-effect factors. BL was set as the baseline comparator for the testing block factor, and participant was set as a cluster variable (i.e., a random-effect factor). Two different models were used; one for the subjective responses (DEQ outcomes) and another for the objective responses (cognitive and cardiovascular outcomes). The two models were identical, except that the one used for the subjective measures did not include the CON session dataset as a covariate because participants were not asked to consume anything, which meant all responses to the DEQ prompts were zero (Not at All). The inclusion of the CON session dataset as a covariate for the objective measures allowed for us to control for: 1) natural variability of cognitive performance and cardiovascular responses over the course of the 7-h testing session, and 2) any learning which may have occurred as a result of repeatedly performing the tasks. Statistical comparisons were not made between the CON and INT datasets nor across time within the CON session.

All statistical tests were completed using jamovi (v. 2.6.25.0, The jamovi project, 2025). The linear mixed-effects models were completed using the General Analyses for Linear Models version 3 plugin (GAMLj3 v3.4.2, https://gamlj.github.io/index.html#general-analyses-for-the-linear-model-in-jamovi). All descriptive statistics are presented as mean ± standard deviation,

and statistical test results are reported as 95% confidence interval (CI) of the differences between testing blocks and BL for that factor, and between males and females for that factor.

Results

Subjective measures

After ingestion of THC, responses to all DEQ measures were elevated compared to BL at multiple timepoints (Fig 2). Using the 0-100mm visual analog scale, participants reported significant subjective drug effects (Fig 2a) beginning at 1h post-ingestion (95% CI [6.1, 30.5mm]) which persisted at 6h (95% CI [4.4, 28.8mm]). Similarly, subjective high (Fig 2b) was increased starting 1h after ingestion (95% CI [4.9, 29.5mm]) and remained elevated at 6h (95% CI [2.0, 26.5mm]). A significant dislike of subjective drug effects (Fig 2c) was observed 1.5-4h post-ingestion (95% CI [1.2, 21.5mm] at 1.5h and [7.1, 27.5mm] at 4h). Participants reported liking feelings associated with the drug effect starting at 1h (95% CI [6.7, 34.5mm]; Fig 2d), and this effect was still present at 6h (95% CI [1.4, 29.2mm]). The desire for more of the drug (Fig 2e) was only elevated between 2.5-4h (95% CI [1.4, 17.8mm] at 2.5h and [2.3, 16.7mm] at 4h). None of the responses to the questions had a main effect of sex. However, there were instances of a sex × time interaction, with females feeling higher than males at 5h (95% CI [5.9, 55.0mm]; Fig 2b), and disliking the drug effect more (Fig 2c) at 3.5 and 4h (95% CI [2.5, 43.2mm] and [1.5, 42.2mm], respectively). Complete results of the linear mixed-effects model for DEQ data can be found in Supplemental Table 1.

Fig 2. Subjective intoxication to 10mg of THC. Self-reported responses to the five prompts of the Drug Effect Questionnaire (DEQ) were quantified using 100mm visual analog scales, anchored by 0 (“Not at All”) and 100 (“Extremely”). Grey symbols depict data from individual participants, whereas black symbols indicate group means. Circles represent females (n=8), triangles represent males (n=7), and black squares represent the means for all participants (n=15). Green regions

indicate testing blocks greater than baseline (BL), whereas asterisks (*) indicate values which were greater for females than males, with the horizontal black line below the asterisks indicating the testing blocks demonstrating this difference.

Cardiovascular measurements

During the INT session, none of HR, systolic BP or diastolic BP had a sex × time interaction or main effect of sex. However, there was a main effect of time for all cardiovascular measures. Compared to BL, HR was elevated at 3h (95% CI [2.5, 20.0bpm]) and remained elevated by 6h (95% CI [0.1, 18.8bpm]; Fig 3a). Systolic BP was elevated at 4h compared to BL (95% CI [1.2, 10.4mmHg]; Fig 3c), whereas diastolic BP was elevated at 1h (95% CI [0.0, 5.4mmHg]; Fig 3e) and 2.5-4h (95% CI [1.0, 6.3mmHg] at 2.5h and [1.5, 6.8mmHg] at 4h). Complete results of the linear mixed-effects model for cardiovascular data can be found in Supplemental Table 2.

Fig 3. Cardiovascular measurements during the Intervention (INT; left panels) and Control (CON; right panels) sessions. Grey symbols depict data from individual participants, whereas black symbols indicate group means. Circles represent females (INT n=8, CON n=3), triangles represent males (INT n=7, CON n=3), and black squares represent the means for all participants (INT n=15, CON n=6). Green regions indicate testing blocks greater than baseline (BL) for the INT session. NB: CON data were included as a covariate in the linear mixed-effects model for the INT data, but a statistical analysis was not performed for the CON data.

Cognitive tasks

Full linear mixed-effects model results for cognitive task outcomes can be found in Supplemental Table 3. After THC ingestion, SRT did not have a sex × time interaction or main effect of sex; however, there was a main effect of time (Fig 4a), with RT slower than BL from 1-6h (95% CI [6.1, 41.0ms] at 1h and [8.1, 46.1ms] at 6h). There were no statistically significant outcomes for intraindividual variability of SRT.

Fig 4. Simple reaction time (SRT) during the Intervention (INT; a) and Control (CON; b) sessions. Grey symbols depict data from individual participants, whereas black symbols indicate

group means. Circles represent females (n=8), triangles represent males (n=7), and black squares represent the means for all participants (n=15). Green regions indicate testing blocks greater than baseline (BL) for the INT session. NB: CON data were included as a covariate in the linear mixed-effects model for the INT data, but a statistical analysis was not performed for the CON data.

In contrast to the SRT data, RT of the "go" trials of the GNG task was influenced by THC at the last time point (95% CI [-55.0, -1.8ms]). The intraindividual variability had a main effect of time, with values greater from 3-4h compared to BL (95% CI [2.9, 23.4ms] at 3h and [2.4, 22.9ms] at 4h). The accuracy of the "no-go" trials had main effects of time and sex (Fig 5a). After THC ingestion, accuracy was lower than BL at 1.5, 2, 3, 4, 5, and 6h (95% CI [−26%, −5%] at 1.5h and [−25%, −3%] at 6h; Fig 5a). Additionally, females demonstrated lower accuracy than males (95% CI [−24%, −2%]; Fig 5a).

Fig 5. Accuracy of "no-go" trials during the Intervention (INT; a) and Control (CON; b) sessions. Grey symbols depict data from individual participants, whereas black symbols indicate group means. Circles represent females (n=8), triangles represent males (n=7), and black squares represent the means for all participants (n=15). Green regions indicate testing blocks lower than baseline (BL) for the INT session. NB: CON data were included as a covariate in the linear mixed-effects model for the INT data, but a statistical analysis was not performed for the CON data.

For the CBT task, which measured short term memory, THC ingestion led to a main effect of time, whereby the maximum sequence length was reduced compared to BL at 1h (95% CI [−2.4, −0.0]; Fig 6a) and from 3.5-5h (95% CI [−2.4, −0.0] at 3.5h and [−2.6, −0.3] at 5h). No other differences were found.

Fig 6. Corsi block tapping test maximum sequence length during the Intervention (INT; a) and Control (CON; b) sessions. Grey symbols depict data from individual participants, whereas black symbols indicate group means. Circles represent females (n=8), triangles represent males (n=7), and black squares represent the means for all participants (n=15). Green regions indicate testing blocks lower than baseline (BL) for the INT session. NB: CON data were included as a

covariate in the linear mixed-effects model for the INT data, but a statistical analysis was not performed for the CON data.

Correlation between subjective and objective results

Considering the strong effect of THC on SRT, we opted to run a post-hoc linear mixed-effects model, with SRT as the dependent variable, timepoint and sex as fixed-effect factors, and the responses to the first DEQ prompt ("Do you feel a drug effect right now?") as a covariate to determine if there was a significant association between the objective SRT results and subjective intoxication. The results of this test demonstrate that SRT and DEQ responses were significantly correlated, with a single unit increase for the DEQ response associated with an increase of 0.2ms for SRT. Additionally, when DEQ responses were included as a covariate, SRT was significantly slower than BL only at 2.5h and 4h. See Supplemental Table 4 for details.

Adverse events

Although no major adverse events occurred, two participants reported experiencing anxiety at some point during the INT session. For one participant, the anxiety tracked their subjective intoxication, which aligned with their previous experiences using cannabis. The other participant reported feeling anxious ~1h following the onset of subjective intoxication. Their anxiety was mitigated when they were able to eat following the 2h testing block, which was also in keeping with this participant's previous experiences using cannabis.

Discussion

The goal of the present study was to characterize the progression of subjective and objective intoxication arising from the ingestion of 10mg of THC via commercially-available THC gel capsules. Both sets of data broadly agree with each other as many indices of intoxication

manifested after 1-2h and remained elevated compared to BL at 5-6h after ingestion, including robust impairments in SRT and GNG accuracy, which correlated with reports of subjective drug effects.

Subjective measures

The results of the DEQ align broadly with previous work to consider the acute effects of edible THC [6,8,9]. However, to our knowledge, the present study is the first to use a commercially-available THC product or a statistical approach designed to: 1) identify onset and offset times of subjective and objective effects, which allows for a better understanding of the time course of intoxication, 2) evaluate possible sex-based differences. As revealed by Fig 2, ingestion of 10mg of THC provides a broad window of time to explore its acute effects, with subjective ratings of intoxication beginning at ~1h and lasting until at least 6h after ingestion (when data collection ceased). Even though all testing blocks between 1-6h are statistically greater than BL, the ideal testing window appears to be 3-4h after ingestion, with a considerable perception of feeling high between 2-5h. These findings support and extend previous work which has examined the time course of intoxication from edible cannabis use [8,27,28], and confirm a later and longer-lasting window of intoxication compared to inhaled cannabis [5,29]. Additionally, the finding that SRT is correlated with responses to the question, “Do you feel a drug effect right now?” is, to the best of our knowledge, the first time that an objective measurement of human behaviour has been shown to correlate with a subjective assessment of cannabis intoxication. Thus, SRT may represent an objective surrogate for subjective perceptions of intoxication arising from edible cannabis use in infrequent users. This result also demonstrates that the subjective perception of intoxication may have specific neural correlates related to voluntary movement initiation, which warrants further study.

Objective measures

Similar to previous reports [10,11], THC intoxication led to slower SRT. To generate a response to a stimulus, three stages of executive processing are thought to occur: 1) stimulus identification, 2) response selection, and 3) motor programming [24]. As the singular response of an SRT task allows for pre-programming of the nature of the response and the associated motor outputs, performance is thought to reflect the latency of the stimulus identification stage. Thus, the observed slowing of SRT after THC ingestion is likely to reflect an impaired ability to properly identify a stimulus. This explanation may also provide insight into why there was poorer accuracy for "no-go" trials of the GNG task when participants became intoxicated; i.e., as their ability to properly distinguish between a "go" vs. "no-go" stimulus worsened, more errors were made. Considering that the GNG task was constituted predominantly of "go" trials, the tendency to erroneously respond more often on the "no-go" trials while intoxicated may reflect a strategy to maximize the number of correct responses in the context of noisy sensory feedback, which would limit the ability to distinguish between stimuli. This perspective is bolstered by the work of [30], which indicated that certain dopaminergic systems in the central nervous system, including those which facilitate the generation of movements in response to specific stimuli (the D1 system), are overstimulated in conjunction with cannabinoid receptor stimulation. This mechanism would likely create noise within the system and ultimately lead to worse performance on tasks that require stimulus-action coupling [31]. Similarly, overstimulation of the D1 system during acute cannabis intoxication may also explain the failure to improve CBT performance after THC ingestion; i.e., one needs to link the visuospatial memory of the order in which stimuli were presented and produce an action that replicates the sequence. Indeed, the D1

system has been shown to be important for the functioning of working memory [32]. Further, [33] identified that acute and chronic cannabis use altered function from the retina to the visual cortex, which suggests that the processing, integration, and action coupling of visual information may be impaired by THC consumption.

For our cardiovascular measures, HR was elevated starting at 3h and remained significantly higher than BL when testing finished 6h after ingesting the THC. The effects of THC on BP were less pronounced as systolic BP was only greater than BL at 4h, whereas diastolic was elevated at 1h and from 2.5-4h. The HR data are consistent with previous findings in response to inhaled THC [5]; however, the observed increases for BP conflict with the findings of Zamarripa and colleagues [9], who reported no effect of 20mg of ingested THC. The primary mechanism through which THC is suspected to impact cardiac activity is via increased cardiac adrenergic receptor stimulation and vagal withdrawal [34], shifting the cardiac autonomic balance to that of greater sympathetic influence and, thus, elevating heart rate. The influence of THC on BP is less clear and, while altered sympathetic nervous system activity is often reported, current literature is conflicted on the direction of this change [35–37].

Ecological validity of dosage and setting

To appropriately address the primary goal of establishing a time course of edible THC intoxication, ecological validity was main a focus of the current study design. Although hindered by the laboratory setting, the other elements of the experiment were chosen to align with this focus. These elements included using an edible THC product that is readily available for purchase, allowing participants to engage in a wide variety of tasks in between testing blocks, and, after an initial wait of 2h, allowing them to consume food and beverages ad libitum. A standardized THC dosage of 10mg was chosen for two reasons: first, this dose produces

significant intoxication without frequently eliciting adverse events that occur at higher doses [5] and, second, 10mg of THC is the highest dose legally available for purchase in a single package of edible cannabis in Canada [38].

Importance of results for researchers, clinicians, policy-makers, general public

In addition to documenting the acute effects of THC on various subjective and objective indices of THC intoxication, this study was designed to establish an appropriate testing window for future research with edibles. However, researchers are not the only group to benefit from these data, as this information is usable by anyone who has a stake in knowing the time course of intoxication from edible THC consumption. Such parties include: 1) clinicians who prescribe cannabis for medicinal purposes, and the patients who will use it, 2) employers who wish to take a data-driven approach to a cannabis policy for their organizations, 3) members of the general public who use cannabis recreationally, and would like to know what symptoms they can expect after consuming a common dosage of edible THC, and 4) policy makers, e.g., those who decide on recreational cannabis product packaging requirements such as the 10mg THC limit on edible products.

Considerations

Although some sex-based differences were identified, we did not specifically ensure that this study was powered for such comparisons. Nor did we standardize testing relative to the phase of the menstrual cycle, which could have increased the variability within the female dataset. Additionally, rather than administer a standardized 10mg of THC, an alternative approach could have been to scale the dosage to anthropometric data (e.g., body mass). However, given an inability to control for interindividual variability in cannabis metabolism and tolerance independent of body size, we decided that providing all participants with a common dosage of a

commercially-available cannabis product aided our pursuit of ecological validity. Further, post-hoc linear mixed-effects models were conducted to examine the relationship of body mass with both DEQ1 ("Do you feel a drug effect right now?") responses and SRT performance. Neither test produced significant results, suggesting, for this sample, there was no correlation between body size and subjective or objective markers of intoxication (Supplemental Table 5). Lastly, the control group included only approximately half the sample of the intervention group, which reduced the power of statistical tests for those outcomes that incorporated the control dataset into the models (i.e., the cognitive and cardiovascular outcomes). With a complete control dataset, more differences may have been detected.

Future Directions

As body mass did not correlate with subjective ratings of intoxication, the shapes of the curves in Figs 2a-c, as well as some sex × time interactions, lead us to conclude that sex-based differences may exist for the subjective experience of THC intoxication. Specifically, males appear to experience peak intoxication earlier than females, whereas females appear to have higher peak values. Notably, although the perception of feeling high was greater for females than males [21], only one of the cognitive-motor outcomes demonstrated sex-based differences; GNG accuracy. Others have suggested that sex-based differences may exist for subjective drug effects and cognitive task performance arising from chronic cannabis use [38,39]. However, evidence that biological sex affects cognitive performance during acute cannabis intoxication remains limited [40], and future work should explore possible sex-based differences for acute subjective and objective effects of cannabis intoxication.

Though THC is the primary psychoactive component of cannabis, the overwhelming majority (~93%) of cannabis consumed in Canada [4], and likely around the world, contains

other cannabinoids, such as cannabidiol (CBD). Many of these cannabinoids are postulated to interact directly with the human motor system. Specifically, CBD is thought to alter THC binding and metabolism when they are co-consumed [41,42]. Thus, CBD should be investigated for the effect it has on motor behaviour by itself and when co-consumed with THC.

Conclusion

To conclude, after ingesting softgel capsules containing 10mg of THC, participants experienced significant subjective and objective effects of intoxication, even when controlling for learning effects and natural daily variation of cognitive-motor task performance. In most instances, the effects of cannabis intoxication appeared ~1h after ingestion, peaked at ~3-4h, and were still present during the last testing block (6h). It is our recommendation that future studies intending to assess the acute influence of cannabis edibles on human behaviour should conduct testing 2-5h post-intervention to align with the greatest levels of intoxication.

Acknowledgements

We thank all participants for contributing their time to this research.

Author Contributions

Paige Copeland: conceptualization, data curation, formal analysis, investigation, methodology, project administration, visualization, writing—original draft, review & editing. Quinn Malone: conceptualization, data curation, formal analysis, investigation, methodology, writing—original draft, review & editing. Brian Dalton: conceptualization, funding acquisition, methodology, supervision, writing—review & editing. Chris McNeil: conceptualization, funding acquisition, methodology, supervision, writing—review & editing.

Disclosures

The authors declare there are no conflicts of interest, financial or otherwise.

Funding

This work was supported by the Natural Sciences and Engineering Research Council of Canada under grant No. DG 5018423-2020 (C.J.M.) and the Stober Foundation Health Fund (C.J.M. & B.H.D.)

References

1. Pain S. A potted history. Nature. 2015 Sep;525(7570):S10–1.

2. Gaoni Y, Mechoulam R. Isolation, Structure, and Partial Synthesis of an Active Constituent of Hashish. J Am Chem Soc. 1964;86(8):1646–7.

3. Mechoulam R, Hanuš LO, Pertwee R, Howlett AC. Early phytocannabinoid chemistry to endocannabinoids and beyond. Nat Rev Neurosci. 2014 Nov;15(11):757–64.

4. Health Canada. Canadian Cannabis Survey 2024: Summary [Internet]. Canada.ca. 2024 [cited 2025 Jun 19]. Available from: https://www.canada.ca/en/health-canada/services/drugs-medication/cannabis/research-data/canadian-cannabis-survey-2024-summary.html#a2.2

5. Spindle TR, Cone EJ, Schlienz NJ, Mitchell JM, Bigelow GE, Flegel R. Acute Effects of Smoked and Vaporized Cannabis in Healthy Adults Who Infrequently Use Cannabis A Crossover Trial. JAMA Netw Open. 2018;1(7):1–14.

6. Vandrey R, Herrmann ES, Mitchell JM, Bigelow GE, Flegel R, LoDico C, et al. Pharmacokinetic profile of oral cannabis in humans: Blood and oral fluid disposition and relation to pharmacodynamic outcomes. J Anal Toxicol. 2017 Mar 1;41(2):83–99.

7. Watanabe K, Yamaori S, Funahashi T, Kimura T, Yamamoto I. Cytochrome P450 enzymes involved in the metabolism of tetrahydrocannabinols and cannabinol by human hepatic microsomes. Life Sci. 2007;80(15):1415–9.

8. Curran VH, Brignell C, Fletcher S, Middleton P, Henry J. Cognitive and subjective dose-response effects of acute oral Δ9-tetrahydrocannabinol (THC) in infrequent cannabis users. Psychopharmacology (Berl). 2002;164(1):61–70.

9. Zamarripa CA, Spindle TR, Surujunarain R, Weerts EM, Bansal S, Unadkat JD, et al. Assessment of Orally Administered Δ9-Tetrahydrocannabinol When Coadministered With Cannabidiol on Δ9-Tetrahydrocannabinol Pharmacokinetics and Pharmacodynamics in Healthy Adults: A Randomized Clinical Trial. JAMA Netw Open. 2023 Feb 13;6(2):e2254752.

10. Hunault CC, Mensinga TT, Böcker KBE, Schipper CMA, Kruidenier M, Leenders MEC, et al. Cognitive and psychomotor effects in males after smoking a combination of tobacco and cannabis containing up to 69 mg delta-9-tetrahydrocannabinol (THC). Psychopharmacology (Berl). 2009;204(1):85–94.

11. Wesnes K, Annas P, Edgar C, Deeprose C, Karlsten R, Philipp A, et al. Nabilone produces marked impairments to cognitive function and changes in subjective state in healthy volunteers. J Psychopharmacol (Oxf). 2010 Nov;24(11):1659–69.

12. Ramaekers J, Kauert G, Theunissen E, Toennes S, Moeller M. Neurocognitive performance during acute THC intoxication in heavy and occasional cannabis users. J Psychopharmacol (Oxf). 2009 May;23(3):266–77.

13. Theunissen EL, Heckman P, De Sousa Fernandes Perna EB, Kuypers KPC, Sambeth A, Blokland A, et al. Rivastigmine but not vardenafil reverses cannabis-induced impairment of verbal memory in healthy humans. Psychopharmacology (Berl). 2015 Jan;232(2):343–53.

14. D'Souza DC, Perry E, MacDougall L, Ammerman Y, Cooper T, Wu YT, et al. The psychotomimetic effects of intravenous delta-9-tetrahydrocannabinol in healthy individuals: Implications for psychosis. Neuropsychopharmacology. 2004;29(8):1558–72.

15. Miller LL, Mcfarland DJ, Cornett TL, Brightwell DR, Wikler A. Marijuana: Effects on free recall and subjective organization of pictures and words. Psychopharmacology (Berl). 1977 Jan 1;55(3):257–62.

16. Figueiredo PR, Tolomeo S, Steele JD, Baldacchino A. Neurocognitive consequences of chronic cannabis use: a systematic review and meta-analysis. Neurosci Biobehav Rev. 2020 Jan 1;108:358–69.

17. Pope HG, Gruber AJ, Yurgelun-Todd D. The residual neuropsychological effects of cannabis: the current status of research. Drug Alcohol Depend. 1995;38(1):25–34.

18. Cuttler C, Spradlin A. Measuring cannabis consumption: Psychometric properties of the Daily Sessions, Frequency, Age of Onset, and Quantity of Cannabis Use Inventory (DFAQ-CU). PLOS ONE. 2017 May 26;12(5):e0178194.

19. Ganzer F, Bröning S, Kraft S, Sack PM, Thomasius R. Weighing the Evidence: A Systematic Review on Long-Term Neurocognitive Effects of Cannabis Use in Abstinent Adolescents and Adults. Neuropsychol Rev. 2016;26(2):186–222.

20. Fogel JS, Kelly TH, Westgate PM, Lile JA. Sex differences in the subjective effects of oral Δ9-THC in cannabis users. Pharmacol Biochem Behav. 2017 Jan 1;152:44–51.

21. Sholler DJ, Strickland JC, Spindle TR, Weerts EM, Vandrey R. Sex differences in the acute effects of oral and vaporized cannabis among healthy adults. Addict Biol. 2021;26(4):e12968.

22. Morean ME, De Wit H, King AC, Sofuoglu M, Rueger SY, O'Malley SS. The drug effects questionnaire: Psychometric support across three drug types. Psychopharmacology (Berl). 2013 May;227(1):177–92.

23. Goyal RK, Guo Y, Mashimo H. Advances in the physiology of gastric emptying. Neurogastroenterol Motil. 2019 Apr;31(4):e13546.

24. Schmidt RA, Lee TD, Winstein CJ, Wulf G, Zelaznik HN. Human Information Processing. In: Motor Control and Learning: A Behavioural Emphasis. 2019. p. 57–94.

25. Corsi PM. Memory and the medial temporal region of the brain [Internet]. McGill University; 1972 [cited 2025 Jun 19]. Available from: https://escholarship.mcgill.ca/concern/theses/05741s554

26. jamovi [Internet]. The jamovi project; 2025. Available from: https://www.jamovi.org

27. Di Ciano P, Zhao S, Kaduri P, Patel S, Bhakta K, Wickens CM, et al. Effects of naturalistic doses of cannabis edibles on cognition and association with blood THC. Psychopharmacology (Berl) [Internet]. 2025 Aug 1 [cited 2025 Aug 18]; Available from: https://doi.org/10.1007/s00213-025-06863-2

28. Hollister LE, Gillespie HK, Ohlsson A, Lindgren JE, Wahlen A, Agurell S. Do Plasma Concentrations of Δ9-Tetrahydrocannabinol Reflect the Degree of Intoxication? J Clin Pharmacol. 1981;21(S1):171S-177S.

29. Matheson J, Sproule B, Di Ciano P, Fares A, Le Foll B, Mann RE, et al. Sex differences in the acute effects of smoked cannabis: evidence from a human laboratory study of young adults. Psychopharmacology (Berl). 2020 Feb 1;237(2):305–16.

30. Kebabian JW, Caine DB. Multiple receptors for dopamine. Nature. 1979 Jan 11;277:93–6.

31. Colzato LS, Hommel B. Cannabis, cocaine, and visuomotor integration: Evidence for a role of dopamine D1 receptors in binding perception and action. Neuropsychologia. 2008;46(5):1570–5.

32. Sawaguchi T, Goldman-Rakic PS. D1 Dopamine Receptors in Prefrontal Cortex: Involvement in Working Memory. Science. 1991 Feb 22;251(4996):947–50.

33. Schwitzer T, Schwan R, Angioi-Duprez K, Ingster-Moati I, Lalanne L, Giersch A, et al. The cannabinoid system and visual processing: A review on experimental findings and clinical presumptions. Eur Neuropsychopharmacol. 2015 Jan;25(1):100–12.

34. Benowitz NL, Rosenberg J, Rogers W, Bachman J, Jones RT. Cardiovascular effects of intravenous delta-9-tetrahydrocannabinol: Autonomic nervous mechanisms. Clin Pharmacol Ther. 1979;25(4):440–6.

35. Page RL, Allen LA, Kloner RA, Carriker CR, Martel C, Morris AA, et al. Medical Marijuana, Recreational Cannabis, and Cardiovascular Health: A Scientific Statement From the American Heart Association. Circulation. 2020 Sep 8;142(10):e131–52.

36. Cheung CP, Nardone M, Lee JB, Baker RE, Millar PJ, Burr JF. Cannabis Inhalation Acutely Reduces Muscle Sympathetic Nerve Activity in Humans. Circulation. 2022 Dec 20;146(25):1972–4.

37. D’Souza AW, Thiessen JS, Cheung CP, Nardone M, Lee JB, Baker RE, et al. Inhibition of muscle sympathetic action potential firing and recruitment patterns following cannabis inhalation in humans. J Appl Physiol. 2025 Jun;138(6):1425–34.

38. Cuttler C, Mischley LK, Sexton M. Sex Differences in Cannabis Use and Effects: A Cross-Sectional Survey of Cannabis Users. Cannabis Cannabinoid Res. 2016 Dec;1(1):166–75.

39. King GR, Ernst T, Deng W, Stenger A, Gonzales RMK, Nakama H, et al. Altered brain activation during visuomotor integration in chronic active cannabis users: Relationship to cortisol levels. J Neurosci. 2011;31(49):17923–31.

40. Arkell TR, Kevin RC, Vinckenbosch F, Lintzeris N, Theunissen E, Ramaekers JG, et al. Sex differences in acute cannabis effects revisited: Results from two randomized, controlled trials. Addict Biol. 2022 Mar;27(2):e13125.

41. Huestis MA. Human cannabinoid pharmacokinetics. Chem Biodivers. 2007;4(8):1770–804.

42. Laprairie RB, Bagher AM, Kelly MEM, Denovan-Wright EM. Cannabidiol is a negative allosteric modulator of the cannabinoid CB1 receptor. Br J Psychol. 2015;172:4790–805.

Figure 1

Click here to access/download;Figure;Figure 1.tiff

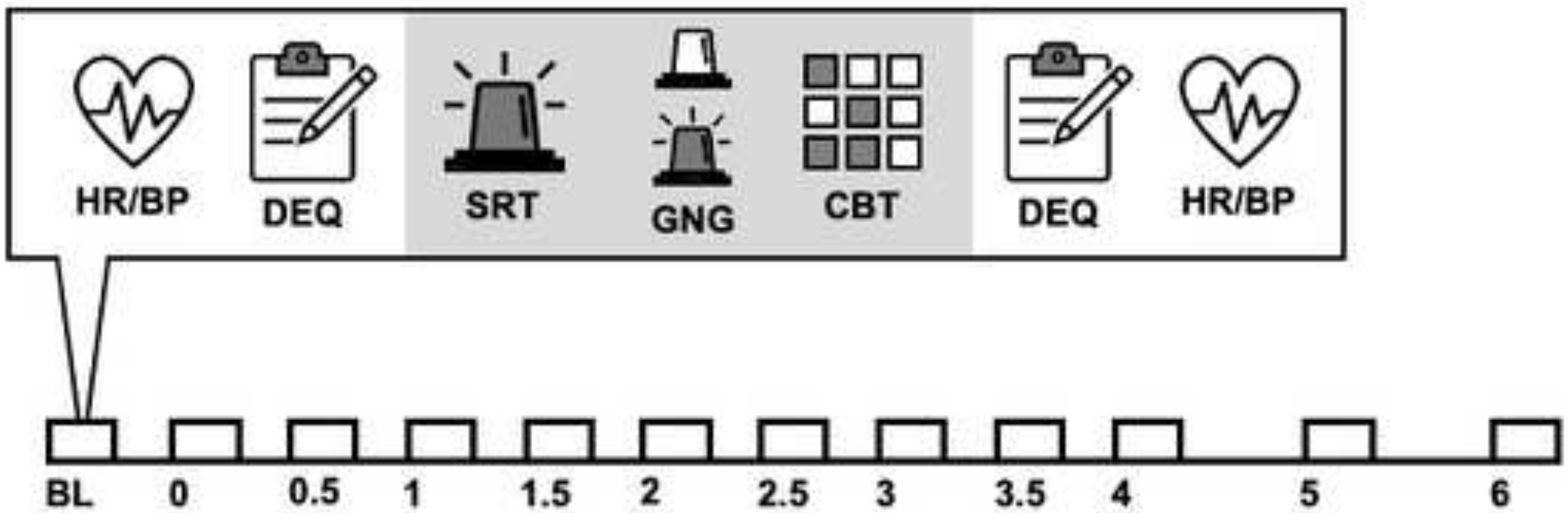

Figure 2

Click here to access/download;Figure;FIgure 2.tiff

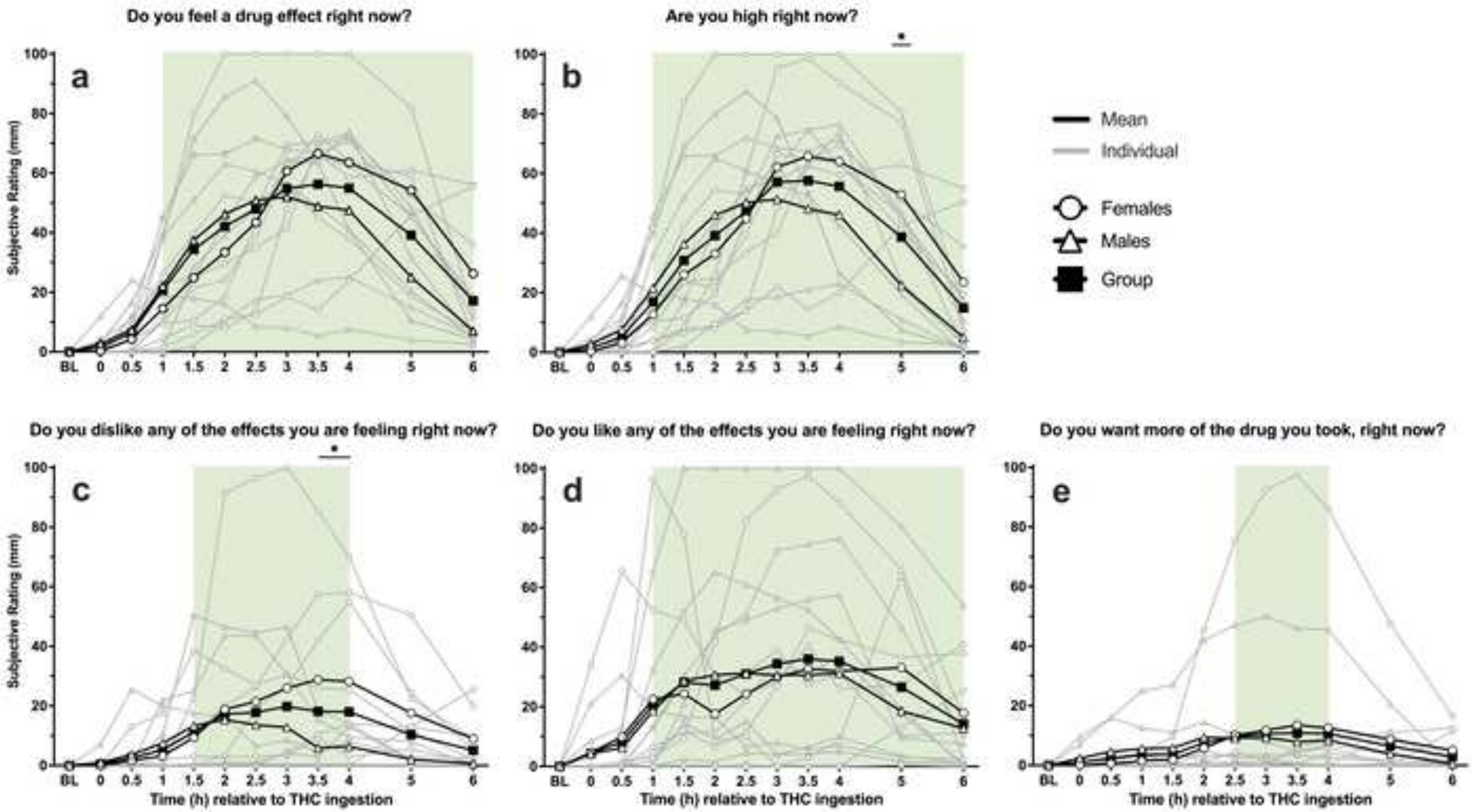

Figure 3

Click here to access/download;Figure;Figure 3.tiff

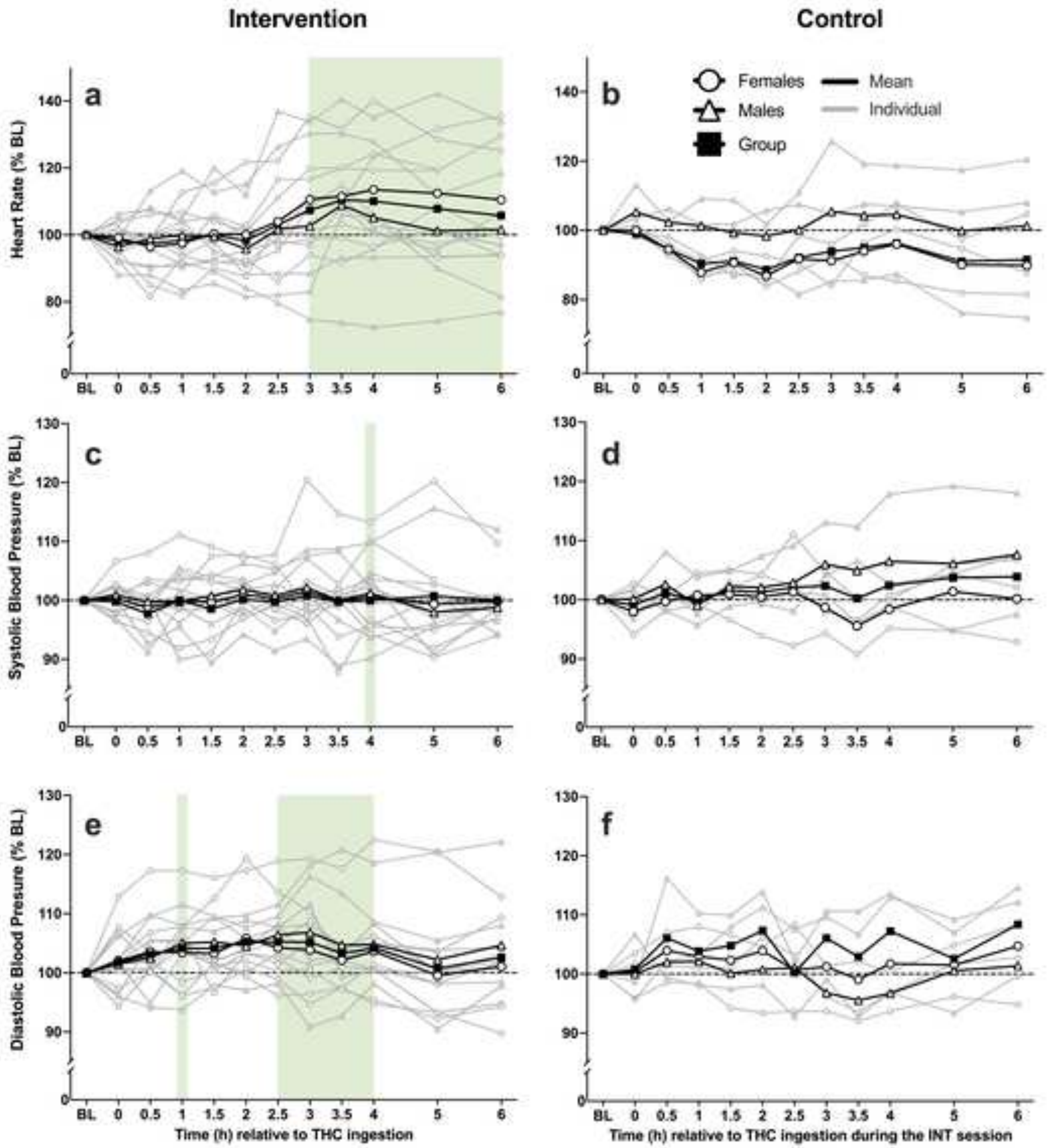

Figure 4

Click here to access/download;Figure;Figure 4.tiff

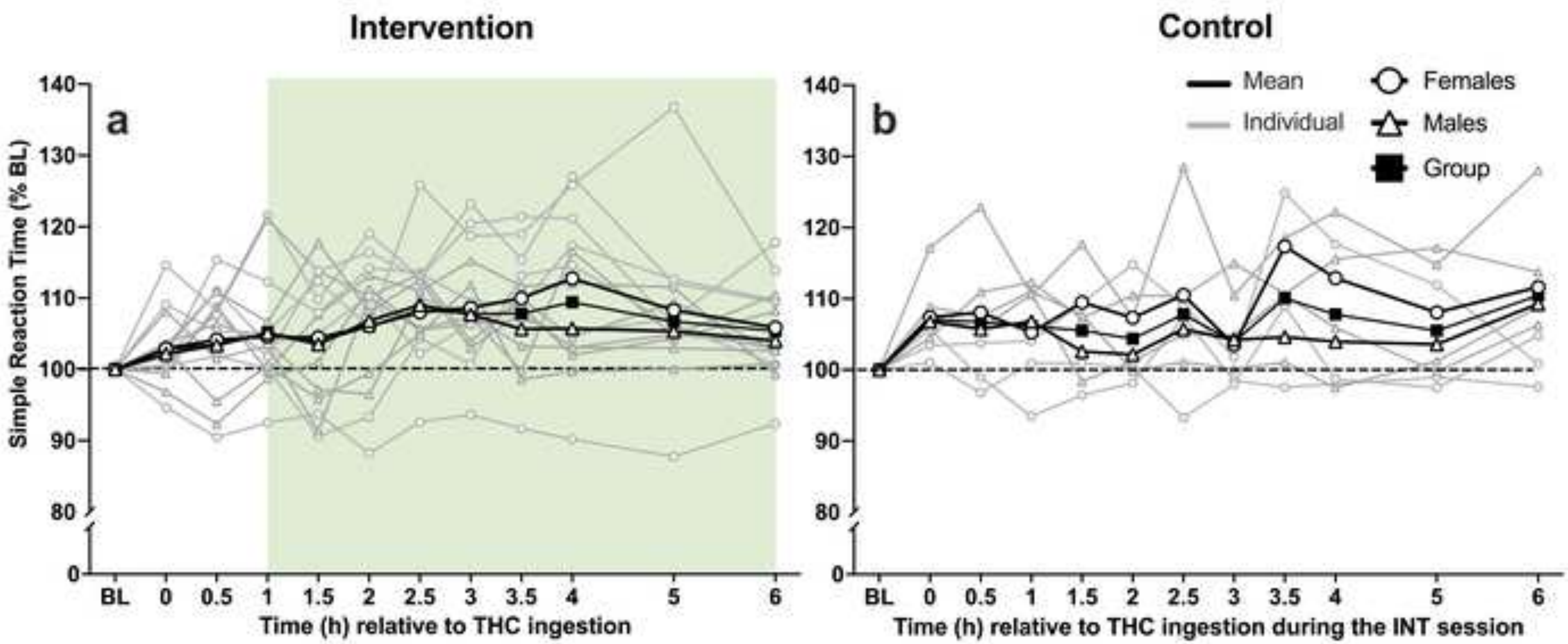

Figure 5

Click here to access/download;Figure;Figure 5.tiff

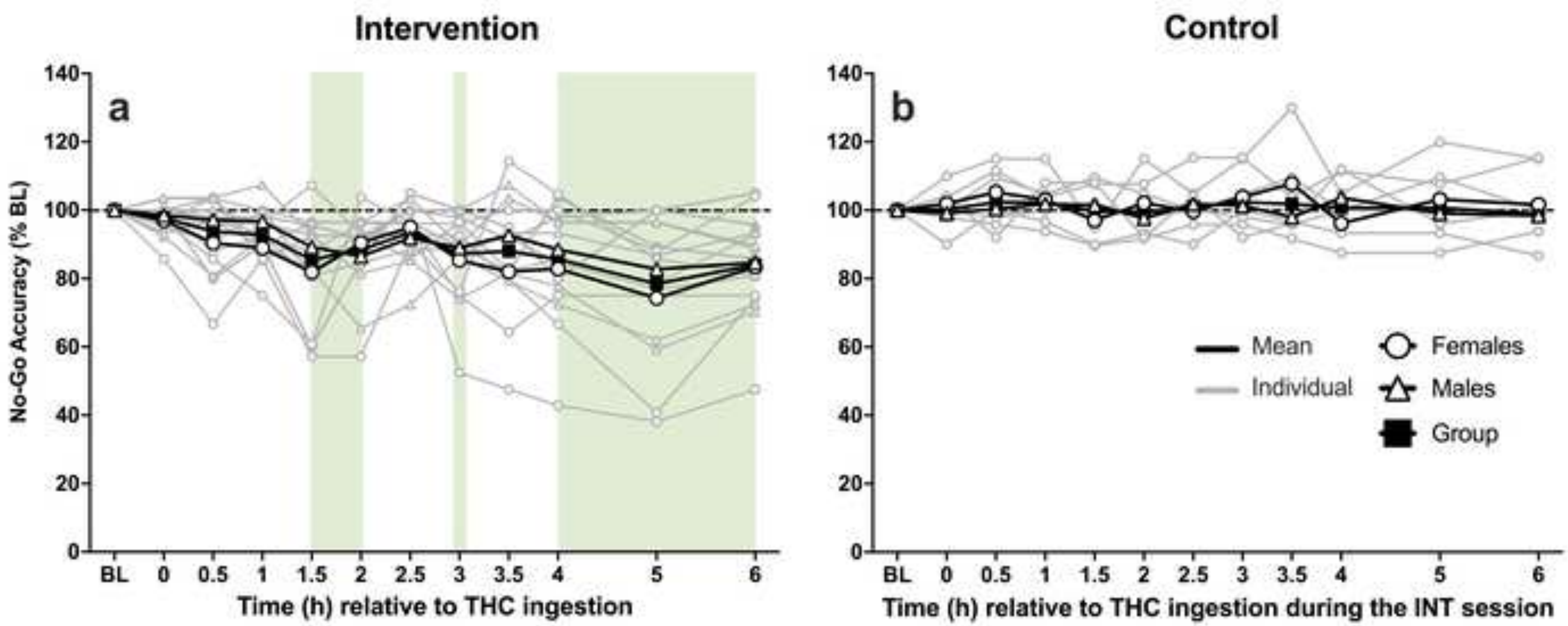

Supplemental Table 1

Click here to access/download
Supporting Information
Supplemental Table 1.docx

Supplemental Table 2

Click here to access/download
Supporting Information
Supplemental Table 2.docx

Supplemental Table 3

Click here to access/download
Supporting Information
Supplemental Table 3- cog.docx

Click here to access/download
Supporting Information
Supplemental Table 4- DEQ SRT correlate.docx

Click here to access/download
Supporting Information
Supplemental Table 5- body mass correlates.docx
