# Supplementary material for: A six-hour time series of the acute effects of ingested cannabis intoxication on a battery of cognitive-motor tasks
Source: PLoS One. 2026 Aug 13;21(8):e0353389. doi: 10.1371/journal.pone.0353389 (PMC13472365; doi:10.1371/journal.pone.0353389)
Supplement: Supplementary file 2 [file pone.0353389.s001.docx]

**Supplemental Table 1**

*Linear mixed effect model results for responses to the Drug Effects Questionnaire (DEQ)*

| **DEQ 1: Do you feel a drug effect right now? (/100)** | | | | |  |
| --- | --- | --- | --- | --- | --- |
|  |  | | **95% CI** | |  |
| **Effect** | **Estimate** | **SE** | **Lower** | **Upper** | **p** |
| (Intercept) | 30.8 | 3.45 | 23.99 | 37.6 | <0.00001 |
| T0 - BL | 1.51 | 6.17 | -10.68 | 13.7 | 0.80661 |
| T0.5 - BL | 5.8 | 6.17 | -6.39 | 18 | 0.34854 |
| T1 – BL | 18.32 | 6.17 | 6.13 | 30.5 | 0.00351 |
| T1.5 – BL | 31.15 | 6.17 | 18.96 | 43.3 | <0.00001 |
| T2 – BL | 39.86 | 6.17 | 27.67 | 52.1 | <0.00001 |
| T2.5 – BL | 47.15 | 6.17 | 34.96 | 59.3 | <0.00001 |
| T3 – BL | 56.31 | 6.17 | 44.12 | 68.5 | <0.00001 |
| T3.5 – BL | 57.71 | 6.17 | 45.52 | 69.9 | <0.00001 |
| T4 – BL | 55.57 | 6.17 | 43.38 | 67.8 | <0.00001 |
| T5 – BL | 39.63 | 6.17 | 27.44 | 51.8 | <0.00001 |
| T6 – BL | 16.62 | 6.17 | 4.44 | 28.8 | 0.00790 |
| F - M | 3.74 | 6.9 | -9.9 | 17.4 | 0.59735 |
| (T0 - BL) × (F - M) | -2.4 | 12.34 | -26.78 | 22 | 0.84597 |
| (T0.5 - BL) × (F - M) | -3.11 | 12.34 | -27.49 | 21.3 | 0.80158 |
| (T1 - BL) × (F - M) | -7.51 | 12.34 | -31.89 | 16.9 | 0.54386 |
| (T1.5 - BL) × (F - M) | -12.55 | 12.34 | -36.93 | 11.8 | 0.31077 |
| (T2 - BL) × (F - M) | -12.85 | 12.34 | -37.23 | 11.5 | 0.29959 |
| (T2.5 - BL) × (F - M) | -7.42 | 12.34 | -31.8 | 17 | 0.54865 |
| (T3 - BL) × (F - M) | 8.63 | 12.34 | -15.75 | 33 | 0.48576 |
| (T3.5 - BL) × (F - M) | 17.71 | 12.34 | -6.67 | 42.1 | 0.15357 |
| (T4 - BL) × (F - M) | 15.99 | 12.34 | -8.39 | 40.4 | 0.19715 |
| (T5 - BL) × (F - M) | 29.12 | 12.34 | 4.74 | 53.5 | 0.01966 |
| (T6 - BL) × (F - M) | 19.25 | 12.34 | -5.13 | 43.6 | 0.12101 |
| **DEQ 2: Do you feel high right now? (/100)** | | | | |  |
|  | | | **95% CI** | |  |
| **Effect** | **Estimate** | **SE** | **Lower** | **Upper** | **p** |
| (Intercept) | 30.26 | 3.48 | 23.37 | 37.1 | <.00001 |
| T0 - BL | 1.51 | 6.22 | -10.78 | 13.8 | 0.80819 |
| T0.5 - BL | 5.38 | 6.22 | -6.91 | 17.7 | 0.38837 |
| T1 – BL | 17.16 | 6.22 | 4.87 | 29.5 | 0.00658 |
| T1.5 – BL | 31.18 | 6.22 | 18.89 | 43.5 | <.00001 |
| T2 – BL | 39.57 | 6.22 | 27.27 | 51.9 | <.00001 |
| T2.5 – BL | 47.54 | 6.22 | 35.25 | 59.8 | <.00001 |
| T3 – BL | 56.74 | 6.22 | 44.45 | 69 | <.00001 |
| T3.5 – BL | 57.01 | 6.22 | 44.72 | 69.3 | <.00001 |
| T4 – BL | 55.07 | 6.22 | 42.77 | 67.4 | <.00001 |
| T5 – BL | 37.65 | 6.22 | 25.36 | 49.9 | <.00001 |
| T6 – BL | 14.25 | 6.22 | 1.96 | 26.5 | 0.02348 |
| F - M | 4.24 | 6.97 | -9.53 | 18 | 0.55353 |
| (T0 - BL) × (F - M) | -2.4 | 12.45 | -26.99 | 22.2 | 0.84724 |
| (T0.5 - BL) × (F - M) | -4.52 | 12.45 | -29.1 | 20.1 | 0.71713 |
| (T1 - BL) × (F - M) | -8.82 | 12.45 | -33.41 | 15.8 | 0.47959 |
| (T1.5 - BL) × (F - M) | -10.36 | 12.45 | -34.94 | 14.2 | 0.40668 |
| (T2 - BL) × (F - M) | -13.01 | 12.45 | -37.59 | 11.6 | 0.29765 |
| (T2.5 - BL) × (F - M) | -5.34 | 12.45 | -29.92 | 19.2 | 0.66855 |
| (T3 - BL) × (F - M) | 10.77 | 12.45 | -13.82 | 35.4 | 0.38837 |
| (T3.5 - BL) × (F - M) | 17.6 | 12.45 | -6.99 | 42.2 | 0.15952 |
| (T4 - BL) × (F - M) | 17.99 | 12.45 | -6.59 | 42.6 | 0.15047 |
| (T5 - BL) × (F - M) | 30.45 | 12.45 | 5.86 | 55 | 0.01564 |
| (T6 - BL) × (F - M) | 18.5 | 12.45 | -6.09 | 43.1 | 0.13935 |
| **DEQ 3: Do you dislike any of the effects you are feeling right now? (/100)** | | | | |  |
|  | | | **95% CI** | |  |
| **Effect** | **Estimate** | **SE** | **Lower** | **Upper** | **p** |
| (Intercept) | 10.265 | 3.08 | 4.18 | 16.3 | 0.00540 |
| T0 - BL | 0.5 | 5.15 | -9.681 | 10.7 | 0.92285 |
| T0.5 - BL | 2.634 | 5.15 | -7.547 | 12.8 | 0.61009 |
| T1 - BL | 5.375 | 5.15 | -4.806 | 15.6 | 0.29873 |
| T1.5 – BL | 11.366 | 5.15 | 1.185 | 21.5 | 0.02902 |
| T2 – BL | 17.085 | 5.15 | 6.904 | 27.3 | 0.00116 |
| T2.5 – BL | 17.571 | 5.15 | 7.39 | 27.8 | 0.00085 |
| T3 – BL | 19.362 | 5.15 | 9.181 | 29.5 | 0.00025 |
| T3.5 – BL | 17.371 | 5.15 | 7.189 | 27.6 | 0.00096 |
| T4 – BL | 17.272 | 5.15 | 7.091 | 27.5 | 0.00103 |
| T5 - BL | 9.817 | 5.15 | -0.364 | 20 | 0.05881 |
| T6 - BL | 4.821 | 5.15 | -5.36 | 15 | 0.35109 |
| F - M | 6.815 | 6.16 | -5.354 | 19 | 0.28864 |
| (T0 - BL) × (F - M) | -1 | 10.31 | -21.362 | 19.4 | 0.92285 |
| (T0.5 - BL) × (F - M) | -2.018 | 10.31 | -22.38 | 18.3 | 0.84507 |
| (T1 - BL) × (F - M) | -4.25 | 10.31 | -24.612 | 16.1 | 0.68072 |
| (T1.5 - BL) × (F - M) | -3.982 | 10.31 | -24.344 | 16.4 | 0.69982 |
| (T2 - BL) × (F - M) | 3.455 | 10.31 | -16.907 | 23.8 | 0.73794 |
| (T2.5 - BL) × (F - M) | 7.857 | 10.31 | -12.505 | 28.2 | 0.44715 |
| (T3 - BL) × (F - M) | 13.152 | 10.31 | -7.21 | 33.5 | 0.20404 |
| (T3.5 - BL) × (F - M) | 22.884 | 10.31 | 2.522 | 43.2 | 0.02798 |
| (T4 - BL) × (F - M) | 21.83 | 10.31 | 1.468 | 42.2 | 0.03591 |
| (T5 - BL) × (F - M) | 15.491 | 10.31 | -4.871 | 35.9 | 0.13507 |
| (T6 - BL) × (F - M) | 8.357 | 10.31 | -12.005 | 28.7 | 0.41883 |
| **DEQ 4: Do you like any of the effects you are feeling right now? (/100)** | | | | |  |
|  | | | **95% CI** | |  |
| **Effect** | **Estimate** | **SE** | **Lower** | **Upper** | **p** |
| (Intercept) | 20.512 | 4.89 | 10.85 | 30.2 | 0.00105 |
| T0 - BL | 4.42 | 7.03 | -9.47 | 18.3 | 0.53052 |
| T0.5 - BL | 8.045 | 7.03 | -5.84 | 21.9 | 0.25435 |
| T1 – BL | 20.571 | 7.03 | 6.69 | 34.5 | 0.00399 |
| T1.5 – BL | 26.545 | 7.03 | 12.66 | 40.4 | 0.00023 |
| T2 – BL | 24.134 | 7.03 | 10.25 | 38 | 0.00078 |
| T2.5 – BL | 27.763 | 7.03 | 13.88 | 41.6 | 0.00012 |
| T3 – BL | 30.286 | 7.03 | 16.4 | 44.2 | 0.00003 |
| T3.5 – BL | 31.665 | 7.03 | 17.78 | 45.6 | 0.00001 |
| T4 – BL | 31.576 | 7.03 | 17.69 | 45.5 | 0.00001 |
| T5 – BL | 25.848 | 7.03 | 11.96 | 39.7 | 0.00033 |
| T6 – BL | 15.295 | 7.03 | 1.41 | 29.2 | 0.03121 |
| F - M | 0.465 | 9.78 | -18.86 | 19.8 | 0.96280 |
| (T0 - BL) × (F - M) | 0.411 | 14.06 | -27.36 | 28.2 | 0.97673 |
| (T0.5 - BL) × (F - M) | 3.661 | 14.06 | -24.11 | 31.4 | 0.79494 |
| (T1 - BL) × (F - M) | 3.857 | 14.06 | -23.92 | 31.6 | 0.78420 |
| (T1.5 - BL) × (F - M) | -4.339 | 14.06 | -32.11 | 23.4 | 0.75803 |
| (T2 - BL) × (F - M) | -13.018 | 14.06 | -40.79 | 14.8 | 0.35602 |
| (T2.5 - BL) × (F - M) | -6.902 | 14.06 | -34.67 | 20.9 | 0.62423 |
| (T3 - BL) × (F - M) | -0.571 | 14.06 | -28.34 | 27.2 | 0.96763 |
| (T3.5 - BL) × (F - M) | 2.045 | 14.06 | -25.73 | 29.8 | 0.88457 |
| (T4 - BL) × (F - M) | 0.723 | 14.06 | -27.05 | 28.5 | 0.95904 |
| (T5 - BL) × (F - M) | 14.554 | 14.06 | -13.22 | 42.3 | 0.30232 |
| (T6 - BL) × (F - M) | 5.161 | 14.06 | -22.61 | 32.9 | 0.71410 |
| **DEQ 5: Do you want more of the drug you took, right now? (/100)** | | | | |  |
|  | | | **95% CI** | |  |
| **Effect** | **Estimate** | **SE** | **Lower** | **Upper** | **p** |
| (Intercept) | 5.754 | 3.14 | -0.451 | 11.96 | 0.08997 |
| T0 - BL | 1.179 | 4.15 | -7.013 | 9.37 | 0.77665 |
| T0.5 - BL | 2.567 | 4.15 | -5.625 | 10.76 | 0.53687 |
| T1 - BL | 3.536 | 4.15 | -4.656 | 11.73 | 0.39527 |
| T1.5 - BL | 3.866 | 4.15 | -4.326 | 12.06 | 0.35273 |
| T2 - BL | 7.585 | 4.15 | -0.607 | 15.78 | 0.06946 |
| T2.5 – BL | 9.603 | 4.15 | 1.411 | 17.79 | 0.02199 |
| T3 – BL | 10.518 | 4.15 | 2.326 | 18.71 | 0.01227 |
| T3.5 – BL | 10.679 | 4.15 | 2.487 | 18.87 | 0.01103 |
| T4 – BL | 10.496 | 4.15 | 2.304 | 18.69 | 0.01245 |
| T5 - BL | 6.272 | 4.15 | -1.919 | 14.46 | 0.13258 |
| T6 - BL | 2.746 | 4.15 | -5.446 | 10.94 | 0.50897 |
| F - M | 0.388 | 6.28 | -12.021 | 12.8 | 0.95164 |
| (T0 - BL) × (F - M) | -2.357 | 8.29 | -18.74 | 14.03 | 0.77665 |
| (T0.5 - BL) × (F - M) | -4.009 | 8.29 | -20.392 | 12.37 | 0.62955 |
| (T1 - BL) × (F - M) | -4.071 | 8.29 | -20.455 | 12.31 | 0.62423 |
| (T1.5 - BL) × (F - M) | -3.982 | 8.29 | -20.365 | 12.4 | 0.63184 |
| (T2 - BL) × (F - M) | -3.545 | 8.29 | -19.928 | 12.84 | 0.66972 |
| (T2.5 - BL) × (F - M) | 0.92 | 8.29 | -15.464 | 17.3 | 0.91186 |
| (T3 - BL) × (F - M) | 2.464 | 8.29 | -13.919 | 18.85 | 0.76679 |
| (T3.5 - BL) × (F - M) | 5.643 | 8.29 | -10.74 | 22.03 | 0.49734 |
| (T4 - BL) × (F - M) | 4.134 | 8.29 | -12.249 | 20.52 | 0.61892 |
| (T5 - BL) × (F - M) | 4.83 | 8.29 | -11.553 | 21.21 | 0.56119 |
| (T6 - BL) × (F - M) | 4.634 | 8.29 | -11.749 | 21.02 | 0.57720 |

*Note.* Dark grey shaded cells represent the 95% confidence intervals (CI) associated with timepoints significantly different from BL. Light grey shaded cells represent the 95% CI associated with timepoints during which a sex × timepoint interaction was observed. SE = standard error. For clarity, the associated p values represent the outcome of a paired, uncorrected t-test for each comparison, as per the model output.
