# Supplementary material for: A six-hour time series of the acute effects of ingested cannabis intoxication on a battery of cognitive-motor tasks
Source: PLoS One. 2026 Aug 13;21(8):e0353389. doi: 10.1371/journal.pone.0353389 (PMC13472365; doi:10.1371/journal.pone.0353389)
Supplement: Supplementary file 3 [file pone.0353389.s002.docx]

**Supplemental Table 2**

*Linear mixed effect model results for cardiovascular measures*

| **Heart Rate (beats/min)** | | | | |  |
| --- | --- | --- | --- | --- | --- |
|  |  | | **95% CI** | |  |
| **Effect** | **Estimate** | **SE** | **Lower** | **Upper** | **p** |
| (Intercept) | 72.572 | 4.487 | 63.528 | 81.615 | 0.00036 |
| T0 - BL | -0.904 | 4.336 | -9.6429 | 7.835 | 0.83588 |
| T0.5 - BL | 2.102 | 4.338 | -6.6395 | 10.844 | 0.63045 |
| T1 - BL | 5.243 | 4.439 | -3.7018 | 14.189 | 0.24401 |
| T1.5 - BL | 5.313 | 4.411 | -3.5774 | 14.204 | 0.23511 |
| T2 - BL | 5.62 | 4.506 | -3.4607 | 14.7 | 0.21902 |
| T2.5 - BL | 8.252 | 4.383 | -0.5819 | 17.086 | 0.06667 |
| T3 – BL | 11.277 | 4.347 | 2.5163 | 20.037 | 0.01302 |
| T3.5 – BL | 10.767 | 4.332 | 2.0358 | 19.498 | 0.01705 |
| T4 – BL | 11.668 | 4.324 | 2.9533 | 20.384 | 0.01003 |
| T5 – BL | 11.678 | 4.415 | 2.7802 | 20.576 | 0.01140 |
| T6 – BL | 9.454 | 4.657 | 0.0691 | 18.838 | 0.04868 |
| F - M | 12.739 | 9.792 | -6.9957 | 32.474 | 0.26072 |
| Con | 0.38 | 0.219 | -0.0606 | 0.821 | 0.09113 |
| (T0 - BL) × (F - M) | 1.475 | 8.672 | -16.0034 | 18.952 | 0.86581 |
| (T0.5 - BL) × (F - M) | 0.552 | 8.741 | -17.0643 | 18.169 | 0.94992 |
| (T1 - BL) × (F - M) | 4.224 | 8.957 | -13.8273 | 22.276 | 0.63959 |
| (T1.5 - BL) × (F - M) | 1.476 | 8.789 | -16.2359 | 19.188 | 0.86741 |
| (T2 - BL) × (F - M) | 5.542 | 8.907 | -12.4082 | 23.493 | 0.53709 |
| (T2.5 - BL) × (F - M) | -0.766 | 8.773 | -18.4459 | 16.915 | 0.93086 |
| (T3 - BL) × (F - M) | 8.899 | 8.934 | -9.1055 | 26.904 | 0.32478 |
| (T3.5 - BL) × (F - M) | 5.206 | 8.795 | -12.5191 | 22.932 | 0.55704 |
| (T4 - BL) × (F - M) | 10.636 | 8.741 | -6.981 | 28.252 | 0.23050 |
| (T5 - BL) × (F - M) | 11.96 | 8.823 | -5.8215 | 29.741 | 0.18242 |
| (T6 - BL) × (F - M) | 7.645 | 9.366 | -11.2311 | 26.52 | 0.41894 |
| **Systolic Blood Pressure (mmHg)** | | | | |  |
|  | | | **95% CI** | |  |
| **Effect** | **Estimate** | **SE** | **Lower** | **Upper** | **p** |
| (Intercept) | 111.648 | 3.354 | 104.890 | 118.407 | <.00001 |
| T0 - BL | 0.124 | 2.257 | -4.425 | 4.673 | 0.94555 |
| T0.5 - BL | -1.499 | 2.260 | -6.053 | 3.055 | 0.50031 |
| T1 - BL | -0.750 | 2.252 | -5.289 | 3.789 | 0.74207 |
| T1.5 - BL | -1.624 | 2.266 | -6.191 | 2.944 | 0.46361 |
| T2 – BL | 1.043 | 2.261 | -3.515 | 5.600 | 0.66082 |
| T2.5 - BL | 0.377 | 2.276 | -4.211 | 4.965 | 0.89330 |
| T3 - BL | 4.127 | 2.283 | -0.474 | 8.728 | 0.08302 |
| T3.5 - BL | 4.042 | 2.253 | -0.498 | 8.582 | 0.07899 |
| T4 – BL | 5.877 | 2.285 | 1.272 | 10.482 | 0.01486 |
| T5 - BL | 0.753 | 2.329 | -3.941 | 5.448 | 0.78756 |
| T6 - BL | 0.826 | 2.470 | -4.151 | 5.803 | 0.77648 |
| F - M | -8.650 | 6.724 | -22.202 | 4.901 | 0.26627 |
| Con | -8.61e-4 | 0.147 | -0.297 | 0.295 | 0.81419 |
| (T0 - BL) × (F - M) | 3.748 | 4.518 | -5.357 | 12.853 | 0.40299 |
| (T0.5 - BL) × (F - M) | 2.497 | 4.529 | -6.629 | 11.624 | 0.56973 |
| (T1 - BL) × (F - M) | -6.165 | 4.513 | -15.260 | 2.930 | 0.17582 |
| (T1.5 - BL) × (F - M) | -3.418 | 4.509 | -12.506 | 5.670 | 0.45919 |
| (T2 - BL) × (F - M) | -3.918 | 4.512 | -13.011 | 5.175 | 0.39730 |
| (T2.5 - BL) × (F - M) | -2.585 | 4.511 | -11.677 | 6.507 | 0.57798 |
| (T3 - BL) × (F - M) | -2.923 | 4.652 | -12.299 | 6.452 | 0.56574 |
| (T3.5 - BL) × (F - M) | -4.259 | 4.745 | -13.823 | 5.305 | 0.35891 |
| (T4 - BL) × (F - M) | -0.258 | 4.686 | -9.698 | 9.183 | 0.99750 |
| (T5 - BL) × (F - M) | -8.004 | 4.566 | -17.207 | 1.198 | 0.09262 |
| (T6 - BL) × (F - M) | -1.529 | 4.965 | -11.536 | 8.477 | 0.79917 |
| **Diastolic Blood Pressure (mmHg)** | | | | |  |
|  | | | **95% CI** | |  |
| **Effect** | **Estimate** | **SE** | **Lower** | **Upper** | **p** |
| (Intercept) | 74.876 | 2.47 | 69.8967 | 79.8545 | 0.00005 |
| T0 - BL | 1.798 | 1.302 | -0.8267 | 4.4218 | 0.17487 |
| T0.5 - BL | 0.917 | 1.344 | -1.7911 | 3.6244 | 0.49882 |
| T1 – BL | 2.681 | 1.325 | 0.0106 | 5.3521 | 0.04949 |
| T1.5 - BL | 1.535 | 1.317 | -1.118 | 4.1885 | 0.25022 |
| T2 - BL | 1.975 | 1.342 | -0.7301 | 4.6808 | 0.14863 |
| T2.5 – BL | 3.671 | 1.303 | 1.0449 | 6.2967 | 0.00740 |
| T3 – BL | 5.084 | 1.306 | 2.4523 | 7.7156 | 0.00035 |
| T3.5 – BL | 5.248 | 1.304 | 2.6193 | 7.876 | 0.00024 |
| T4 – BL | 4.139 | 1.31 | 1.4994 | 6.7788 | 0.00295 |
| T5 - BL | 1.04 | 1.308 | -1.5953 | 3.6763 | 0.43082 |
| T6 - BL | 0.56 | 1.441 | -2.3435 | 3.4641 | 0.69934 |
| F - M | -5 | 4.959 | -14.9954 | 4.9944 | 0.38238 |
| Con | 0.408 | 0.112 | 0.1818 | 0.6351 | 0.00071 |
| (T0 - BL) × (F - M) | -0.428 | 2.604 | -5.6769 | 4.8201 | 0.87014 |
| (T0.5 - BL) × (F - M) | -0.46 | 2.622 | -5.7439 | 4.8236 | 0.86154 |
| (T1 - BL) × (F - M) | -3.224 | 2.606 | -8.4768 | 2.0284 | 0.22304 |
| (T1.5 - BL) × (F - M) | -3.472 | 2.63 | -8.7728 | 1.8291 | 0.19409 |
| (T2 - BL) × (F - M) | 0.573 | 2.656 | -4.7796 | 5.9251 | 0.83031 |
| (T2.5 - BL) × (F - M) | -3.689 | 2.605 | -8.938 | 1.5601 | 0.16417 |
| (T3 - BL) × (F - M) | -2.292 | 2.714 | -7.7629 | 3.1784 | 0.40318 |
| (T3.5 - BL) × (F - M) | -3.803 | 2.678 | -9.2001 | 1.5944 | 0.16302 |
| (T4 - BL) × (F - M) | 0.665 | 2.745 | -4.8676 | 6.1985 | 0.80965 |
| (T5 - BL) × (F - M) | -5.921 | 2.608 | -11.178 | -0.6647 | 0.02848 |
| (T6 - BL) × (F - M) | -5.645 | 2.834 | -11.3555 | 0.0661 | 0.05293 |

*Note.* Dark grey shaded cells represent the 95% confidence intervals (CI) associated with timepoints significantly different from BL. SE = standard error. For clarity, the associated p values represent the outcome of a paired, uncorrected t-test for each comparison, as per the model output.
