# Supplementary material for: A six-hour time series of the acute effects of ingested cannabis intoxication on a battery of cognitive-motor tasks
Source: PLoS One. 2026 Aug 13;21(8):e0353389. doi: 10.1371/journal.pone.0353389 (PMC13472365; doi:10.1371/journal.pone.0353389)
Supplement: Supplementary file 4 [file pone.0353389.s003.docx]

| **Supplemental Table 3** *Linear mixed effect model results for cognitive-motor measures* **Simple Reaction Time (ms)** | | | | | |
| --- | --- | --- | --- | --- | --- |
|  | | | **95% CI** | |  |
| **Effect** | **Estimate** | **SE** | **Lower** | **Upper** | **p** |
| (Intercept) | 268.13 | 11.776 | 244.539 | 291.721 | <.00001 |
| T0 - BL | 14.14 | 8.828 | -3.546 | 31.825 | 0.11513 |
| T0.5 - BL | 14.841 | 8.807 | -2.801 | 32.483 | 0.09779 |
| T1 – BL | 23.589 | 8.716 | 6.128 | 41.049 | 0.00912 |
| T1.5 – BL | 21.83 | 8.716 | 4.37 | 39.29 | 0.01535 |
| T2 – BL | 25.856 | 8.603 | 8.623 | 43.089 | 0.00404 |
| T2.5 – BL | 29.554 | 8.955 | 11.615 | 47.492 | 0.00172 |
| T3 – BL | 28.793 | 8.54 | 11.685 | 45.902 | 0.00140 |
| T3.5 – BL | 25.091 | 9.376 | 6.309 | 43.873 | 0.00983 |
| T4 – BL | 33.353 | 8.994 | 15.335 | 51.372 | 0.00050 |
| T5 – BL | 28.267 | 8.697 | 10.846 | 45.689 | 0.00200 |
| T6 – BL | 27.062 | 9.481 | 8.07 | 46.054 | 0.00610 |
| F - M | 13.779 | 23.599 | -33.496 | 61.054 | 0.58622 |
| Con | -0.129 | 0.158 | -0.445 | 0.186 | 0.41583 |
| (T0 - BL) × (F - M) | 19.741 | 16.833 | -13.978 | 53.461 | 0.24614 |
| (T0.5 - BL) × (F - M) | 16.829 | 16.854 | -16.934 | 50.591 | 0.32259 |
| (T1 - BL) × (F - M) | 8.317 | 16.841 | -25.419 | 42.053 | 0.62345 |
| (T1.5 - BL) × (F - M) | 5.451 | 17.031 | -28.665 | 39.568 | 0.75016 |
| (T2 - BL) × (F - M) | 13.368 | 16.942 | -20.571 | 47.307 | 0.43362 |
| (T2.5 - BL) × (F - M) | 9.647 | 16.927 | -24.262 | 43.555 | 0.57116 |
| (T3 - BL) × (F - M) | 23.055 | 16.833 | -10.665 | 56.776 | 0.17659 |
| (T3.5 - BL) × (F - M) | 26.275 | 17.502 | -8.787 | 61.336 | 0.13919 |
| (T4 - BL) × (F - M) | 34.176 | 17.164 | -0.207 | 68.559 | 0.05161 |
| (T5 - BL) × (F - M) | 32.358 | 16.915 | -1.528 | 66.244 | 0.06118 |
| (T6 - BL) × (F - M) | 19.926 | 17.529 | -15.189 | 55.041 | 0.26078 |
| **Simple Reaction Time Variability (ms)** | | | | |  |
|  | | | **95% CI** | |  |
| **Effect** | **Estimate** | **SE** | **Lower** | **Upper** | **p** |
| (Intercept) | 51.01 | 1.744 | 47.515 | 54.505 | 0.00584 |
| T0 - BL | -1.345 | 5.36 | -12.081 | 9.392 | 0.80290 |
| T0.5 - BL | 2.832 | 5.27 | -7.725 | 13.39 | 0.59349 |
| T1 - BL | 7.637 | 5.226 | -2.833 | 18.106 | 0.15086 |
| T1.5 - BL | 2.439 | 5.414 | -8.407 | 13.285 | 0.65420 |
| T2 - BL | 4.518 | 5.298 | -6.095 | 15.131 | 0.39797 |
| T2.5 - BL | 6.028 | 5.318 | -4.625 | 16.681 | 0.26254 |
| T3 - BL | 9.912 | 5.319 | -0.744 | 20.567 | 0.06841 |
| T3.5 - BL | 8.126 | 5.453 | -2.797 | 19.05 | 0.14203 |
| T4 - BL | 8.887 | 5.361 | -1.852 | 19.625 | 0.10357 |
| T5 - BL | 6.155 | 5.354 | -4.57 | 16.88 | 0.25577 |
| T6 - BL | 3.982 | 5.67 | -7.376 | 15.341 | 0.48547 |
| F - M | 3.333 | 3.949 | -4.578 | 11.243 | 0.49115 |
| CON | 0.339 | 0.11 | 0.119 | 0.559 | 0.01294 |
| (T0 - BL) × (F - M) | 4.084 | 10.462 | -16.873 | 25.042 | 0.69807 |
| (T0.5 - BL) × (F - M) | 18.921 | 10.444 | -2 | 39.843 | 0.07669 |
| (T1 - BL) × (F - M) | 1.867 | 10.474 | -19.114 | 22.849 | 0.85928 |
| (T1.5 - BL) × (F - M) | 1.066 | 10.481 | -19.929 | 22.061 | 0.91945 |
| (T2 - BL) × (F - M) | -3.143 | 10.467 | -24.112 | 17.826 | 0.76534 |
| (T2.5 - BL) × (F - M) | 6.752 | 10.603 | -14.489 | 27.993 | 0.52727 |
| (T3 - BL) × (F - M) | 14.308 | 10.683 | -7.094 | 35.709 | 0.18659 |
| (T3.5 - BL) × (F - M) | 12.862 | 10.434 | -8.039 | 33.763 | 0.22408 |
| (T4 - BL) × (F - M) | 3.968 | 10.434 | -16.935 | 24.871 | 0.70553 |
| (T5 - BL) × (F - M) | 4.223 | 10.472 | -16.755 | 25.201 | 0.68861 |
| (T6 - BL) × (F - M) | 8.204 | 10.927 | -13.685 | 30.093 | 0.45646 |
|  |  |  |  |  |  |
| **Go/No-Go “Go” Reaction Time (ms)** | | | | |  |
|  | | | **95% CI** | |  |
| **Effect** | **Estimate** | **SE** | **Lower** | **Upper** | **p** |
| (Intercept) | 316.279 | 7.533 | 301.183 | 331.375 | <.00001 |
| T0 - BL | 0.947 | 12.865 | -24.8348 | 26.728 | 0.94162 |
| T0.5 - BL | 0.142 | 12.775 | -25.4595 | 25.744 | 0.99115 |
| T1 - BL | -13.859 | 12.715 | -39.3411 | 11.623 | 0.28082 |
| T1.5 - BL | -15.873 | 12.763 | -41.4517 | 9.705 | 0.21925 |
| T2 - BL | -20.395 | 12.715 | -45.8773 | 5.087 | 0.11485 |
| T2.5 - BL | -7.118 | 12.715 | -32.5993 | 18.363 | 0.57803 |
| T3 - BL | -2.359 | 12.715 | -27.8406 | 23.122 | 0.85352 |
| T3.5 - BL | -14.154 | 13.66 | -41.5286 | 13.221 | 0.30498 |
| T4 - BL | -8.266 | 12.724 | -33.7646 | 17.233 | 0.51883 |
| T5 - BL | -10.182 | 12.767 | -35.767 | 15.404 | 0.42882 |
| T6 - BL | -28.43 | 13.264 | -55.0119 | -1.847 | 0.03681 |
| F - M | -21.67 | 15.246 | -52.2227 | 8.883 | 0.22767 |
| Con | 0.284 | 0.136 | 0.0119 | 0.557 | 0.04394 |
| (T0 - BL) × (F - M) | 10.908 | 25.517 | -40.2303 | 62.046 | 0.67082 |
| (T0.5 - BL) × (F - M) | -6.815 | 25.667 | -58.2535 | 44.623 | 0.79165 |
| (T1 - BL) × (F - M) | -27.37 | 25.432 | -78.3369 | 23.597 | 0.28687 |
| (T1.5 - BL) × (F - M) | -3.555 | 25.597 | -54.8519 | 47.741 | 0.89007 |
| (T2 - BL) × (F - M) | -3.214 | 25.662 | -54.6423 | 48.215 | 0.90083 |
| (T2.5 - BL) × (F - M) | 15.026 | 25.465 | -36.0073 | 66.06 | 0.55773 |
| (T3 - BL) × (F - M) | 1.483 | 25.431 | -49.4825 | 52.449 | 0.95372 |
| (T3.5 - BL) × (F - M) | -15.807 | 27.361 | -70.64 | 39.026 | 0.56598 |
| (T4 - BL) × (F - M) | 12.241 | 25.581 | -39.0241 | 63.505 | 0.63430 |
| (T5 - BL) × (F - M) | 34.641 | 25.43 | -16.3225 | 85.604 | 0.17909 |
| (T6 - BL) × (F - M) | -10.663 | 26.534 | -63.8377 | 42.511 | 0.68942 |
| **Go/No-Go “Go” Reaction Time Variability (ms)** | | | | |  |
|  | | | **95% CI** | |  |
| **Effect** | **Estimate** | **SE** | **Lower** | **Upper** | **p** |
| (Intercept) | 51.4306 | 3.1666 | 45.087 | 57.774 | 0.00003 |
| T0 - BL | 2.5621 | 5.1481 | -7.751 | 12.875 | 0.62075 |
| T0.5 - BL | 5.2633 | 5.152 | -5.057 | 15.584 | 0.31156 |
| T1 - BL | 8.6774 | 5.1283 | -1.596 | 18.951 | 0.09648 |
| T1.5 - BL | 7.0833 | 5.1855 | -3.305 | 17.471 | 0.17762 |
| T2 - BL | 7.4793 | 5.1457 | -2.829 | 17.787 | 0.15193 |
| T2.5 - BL | 9.3265 | 5.1464 | -0.983 | 19.636 | 0.07557 |
| T3 – BL | 13.1706 | 5.126 | 2.902 | 23.439 | 0.01303 |
| T3.5 – BL | 13.0804 | 5.1266 | 2.811 | 23.35 | 0.01363 |
| T4 – BL | 12.6579 | 5.121 | 2.399 | 22.917 | 0.01668 |
| T5 - BL | 9.827 | 5.1223 | -0.434 | 20.088 | 0.06042 |
| T6 - BL | 9.3207 | 5.3226 | -1.342 | 19.983 | 0.08567 |
| F - M | 8.957 | 6.3655 | -3.795 | 21.709 | 0.22194 |
| Con | 0.013 | 0.061 | -0.109 | 0.135 | 0.83276 |
| (T0 - BL) × (F - M) | 1.8641 | 10.265 | -18.699 | 22.427 | 0.85658 |
| (T0.5 - BL) × (F - M) | 17.7424 | 10.3149 | -2.921 | 38.406 | 0.09119 |
| (T1 - BL) × (F - M) | -1.1305 | 10.2661 | -21.696 | 19.435 | 0.91273 |
| (T1.5 - BL) × (F - M) | 4.2859 | 10.2633 | -16.274 | 24.846 | 0.67792 |
| (T2 - BL) × (F - M) | -0.3332 | 10.2851 | -20.937 | 20.27 | 0.97428 |
| (T2.5 - BL) × (F - M) | 1.0927 | 10.2678 | -19.476 | 21.662 | 0.91565 |
| (T3 - BL) × (F - M) | 7.2461 | 10.238 | -13.263 | 27.755 | 0.48218 |
| (T3.5 - BL) × (F - M) | 12.6764 | 10.2374 | -7.832 | 33.184 | 0.22107 |
| (T4 - BL) × (F - M) | 4.8142 | 10.3927 | -16.005 | 25.633 | 0.64506 |
| (T5 - BL) × (F - M) | 1.7941 | 10.364 | -18.967 | 22.556 | 0.86321 |
| (T6 - BL) × (F - M) | 3.4157 | 10.6251 | -17.869 | 24.7 | 0.74911 |
| **Go/No-Go “No-Go” Accuracy (/1)** | | | | |  |
|  | | | **95% CI** | |  |
| **Effect** | **Estimate** | **SE** | **Lower** | **Upper** | **p** |
| (Intercept) | 0.76258 | 0.0246 | 0.713 | 0.8118 | <.00001 |
| T0 - BL | -0.0287 | 0.0532 | -0.135 | 0.0779 | 0.59192 |
| T0.5 - BL | -0.0703 | 0.0534 | -0.177 | 0.0368 | 0.19436 |
| T1 - BL | -0.0754 | 0.0534 | -0.182 | 0.0316 | 0.16360 |
| T1.5 – BL | -0.1568 | 0.0532 | -0.263 | -0.0501 | 0.00480 |
| T2 – BL | -0.1515 | 0.0532 | -0.258 | -0.0448 | 0.00631 |
| T2.5 – BL | -0.0755 | 0.0532 | -0.182 | 0.0312 | 0.16211 |
| T3 – BL | -0.1275 | 0.0534 | -0.235 | -0.0204 | 0.02064 |
| T3.5 – BL | -0.0665 | 0.0576 | -0.182 | 0.0488 | 0.25281 |
| T4 – BL | -0.1387 | 0.0532 | -0.245 | -0.032 | 0.01192 |
| T5 – BL | -0.2234 | 0.0532 | -0.33 | -0.1167 | 0.00011 |
| T6 – BL | -0.1409 | 0.0552 | -0.252 | -0.0302 | 0.01370 |
| F – M | -0.1306 | 0.0549 | -0.241 | -0.0205 | 0.05825 |
| Con | 0.64029 | 0.2199 | 0.2 | 1.081 | 0.00843 |
| (T0 - BL) × (F - M) | -0.0505 | 0.1065 | -0.264 | 0.163 | 0.63768 |
| (T0.5 - BL) × (F - M) | -0.1355 | 0.1067 | -0.349 | 0.0784 | 0.20986 |
| (T1 - BL) × (F - M) | -0.0794 | 0.1064 | -0.293 | 0.1339 | 0.45884 |
| (T1.5 - BL) × (F - M) | -0.0788 | 0.1067 | -0.293 | 0.1351 | 0.46371 |
| (T2 - BL) × (F - M) | -0.0298 | 0.1068 | -0.244 | 0.1842 | 0.78148 |
| (T2.5 - BL) × (F - M) | 0.08748 | 0.1065 | -0.126 | 0.3009 | 0.41519 |
| (T3 - BL) × (F - M) | 0.0601 | 0.1065 | -0.153 | 0.2735 | 0.57493 |
| (T3.5 - BL) × (F - M) | 0.04373 | 0.1164 | -0.19 | 0.277 | 0.70856 |
| (T4 - BL) × (F - M) | 0.04926 | 0.1073 | -0.166 | 0.2644 | 0.64819 |
| (T5 - BL) × (F - M) | -0.0072 | 0.1067 | -0.221 | 0.2066 | 0.94645 |
| (T6 - BL) × (F - M) | 0.0435 | 0.1105 | -0.178 | 0.2649 | 0.69538 |
| **Corsi Block Tapping Test Maximum Sequence Length** | | | | |  |
|  | | | **95% CI** | |  |
| **Effect** | **Estimate** | **SE** | **Lower** | **Upper** | **p** |
| (Intercept) | 6.7732 | 0.246 | 6.281 | 7.2658 | 0.00011 |
| T0 - BL | -0.3406 | 0.576 | -1.494 | 0.8127 | 0.55674 |
| T0.5 - BL | -0.0851 | 0.576 | -1.24 | 1.0694 | 0.88316 |
| T1 – BL | -1.1938 | 0.58 | -2.355 | -0.0328 | 0.04446 |
| T1.5 – BL | -0.1268 | 0.576 | -1.281 | 1.0277 | 0.82675 |
| T2 – BL | 0.0453 | 0.577 | -1.111 | 1.2016 | 0.93773 |
| T2.5 – BL | -0.9384 | 0.581 | -2.102 | 0.2257 | 0.11238 |
| T3 – BL | -1.1214 | 0.577 | -2.278 | 0.0349 | 0.05754 |
| T3.5 – BL | -1.1956 | 0.586 | -2.369 | -0.0222 | 0.04624 |
| T4 – BL | -1.3913 | 0.583 | -2.559 | -0.2238 | 0.02062 |
| T5 – BL | -1.4474 | 0.594 | -2.638 | -0.2566 | 0.01821 |
| T6 - BL | -1.1499 | 0.597 | -2.345 | 0.0452 | 0.05948 |
| F - M | -0.7665 | 0.509 | -1.787 | 0.2541 | 0.22235 |
| Con | 0.4347 | 0.134 | 0.167 | 0.7029 | 0.00388 |
| (T0 - BL) × (F - M) | -1.8985 | 1.157 | -4.215 | 0.4184 | 0.10678 |
| (T0.5 - BL) × (F - M) | -1.4529 | 1.15 | -3.757 | 0.851 | 0.21223 |
| (T1 - BL) × (F - M) | -1.2355 | 1.151 | -3.542 | 1.0709 | 0.28827 |
| (T1.5 - BL) × (F - M) | -2.0362 | 1.15 | -4.34 | 0.2676 | 0.08263 |
| (T2 - BL) × (F - M) | -1.6267 | 1.162 | -3.955 | 0.7013 | 0.16751 |
| (T2.5 - BL) × (F - M) | -1.6594 | 1.154 | -3.972 | 0.6532 | 0.15666 |
| (T3 - BL) × (F - M) | -1.0906 | 1.154 | -3.403 | 1.2219 | 0.34920 |
| (T3.5 - BL) × (F - M) | -1.5218 | 1.151 | -3.827 | 0.7837 | 0.19198 |
| (T4 - BL) × (F - M) | -1.1304 | 1.15 | -3.434 | 1.1734 | 0.33029 |
| (T5 - BL) × (F - M) | -2.1774 | 1.174 | -4.528 | 0.1737 | 0.06912 |
| (T6 - BL) × (F - M) | -1.3234 | 1.197 | -3.72 | 1.0737 | 0.27389 |

*Note.* Dark grey shaded cells represent the 95% confidence intervals (CI) associated with timepoints significantly different from BL. Light grey shaded cells represent the 95% CI associated with a main effect of sex. SE = standard error. For clarity, the associated p values represent the outcome of a paired, uncorrected t-test for each comparison, as per the model output.
