# Supplementary material for: A six-hour time series of the acute effects of ingested cannabis intoxication on a battery of cognitive-motor tasks
Source: PLoS One. 2026 Aug 13;21(8):e0353389. doi: 10.1371/journal.pone.0353389 (PMC13472365; doi:10.1371/journal.pone.0353389)
Supplement: Supplementary file 5 [file pone.0353389.s004.docx]

**Supplemental Table 4**

*Linear mixed effect model results for simple reaction time using subjective drug effects as a covariate*

| **Simple Reaction Time (ms)** | | | | |
| --- | --- | --- | --- | --- |
|  |  | | **95% CI** | |
| **Effect** | **Estimate** | **SE** | **Lower** | **Upper** |
| (Intercept) | 263.937 | 7.2569 | 249.6000 | 278.275 |
| T0 - BL | 6.939 | 4.9977 | -2.9352 | 16.813 |
| T0.5 - BL | 9.129 | 5.0127 | -0.7740 | 19.033 |
| T1 - BL | 9.182 | 5.1461 | -0.9853 | 19.349 |
| T1.5 - BL | 4.416 | 5.4123 | -6.2768 | 15.109 |
| T2 - BL | 8.685 | 5.6587 | -2.4948 | 19.865 |
| T2.5 - BL | 12.392 | 5.9002 | 0.7349 | 24.049 |
| T3 - BL | 9.653 | 6.2436 | -2.6827 | 21.988 |
| T3.5 - BL | 8.564 | 6.2994 | -3.8817 | 21.010 |
| T4 - BL | 12.640 | 6.2141 | 0.3628 | 24.917 |
| T5 - BL | 9.794 | 5.6515 | -1.3718 | 20.960 |
| T6 - BL | 9.816 | 5.1204 | -0.3002 | 19.932 |
| DEQ1 | 0.207 | 0.0661 | 0.0766 | 0.338 |
| F - M | 11.839 | 14.5159 | -16.8401 | 40.518 |
| (T0 - BL) ✻ (F - M) | 4.372 | 9.9931 | -15.3709 | 24.116 |
| (T0.5 - BL) ✻ (F - M) | 4.690 | 9.9938 | -15.0548 | 24.435 |
| (T1 - BL) ✻ (F - M) | 2.547 | 10.0027 | -17.2156 | 22.309 |
| (T1.5 - BL) ✻ (F - M) | 7.058 | 10.0233 | -12.7454 | 26.861 |
| (T2 - BL) ✻ (F - M) | 3.162 | 10.0249 | -16.6444 | 22.968 |
| (T2.5 - BL) ✻ (F - M) | 1.541 | 10.0024 | -18.2203 | 21.303 |
| (T3 - BL) ✻ (F - M) | 3.099 | 10.0113 | -16.6799 | 22.879 |
| (T3.5 - BL) ✻ (F - M) | 9.640 | 10.0660 | -10.2477 | 29.527 |
| (T4 - BL) ✻ (F - M) | 16.863 | 10.0529 | -2.9985 | 36.724 |
| (T5 - BL) ✻ (F - M) | 3.746 | 10.1845 | -16.3752 | 23.868 |
| (T6 - BL) ✻ (F - M) | 2.839 | 10.0788 | -17.0731 | 22.752 |

*Note.* Dark grey shaded cells represent the 95% confidence intervals (CI) associated with timepoints significantly different from BL. Light grey shaded cells represent the 95% CI associated with timepoints during which a sex × timepoint interaction was observed. SE = standard error.
