# Supplementary material for: A six-hour time series of the acute effects of ingested cannabis intoxication on a battery of cognitive-motor tasks
Source: PLoS One. 2026 Aug 13;21(8):e0353389. doi: 10.1371/journal.pone.0353389 (PMC13472365; doi:10.1371/journal.pone.0353389)
Supplement: Supplementary file 6 [file pone.0353389.s005.docx]

**Supplemental Table 5**

*Linear mixed effect model results for body mass correlations with simple reaction time and Drug Effect Questionnaire prompt 1 (DEQ 1)*

| **DEQ 1: Do you feel a drug effect right now? (/100)** | | | | |
| --- | --- | --- | --- | --- |
|  |  | | **95% CI** | |
| **Effect** | **Estimate** | **SE** | **Lower** | **Upper** |
| (Intercept) | 258.0781 | 17.0652 | 223.8786 | 292.2776 |
| T0 - BL | 14.1538 | 8.8304 | -3.5427 | 31.8503 |
| T0.5 - BL | 14.8545 | 8.8087 | -2.7985 | 32.5075 |
| T1 – BL | 23.6006 | 8.7177 | 6.1299 | 41.0714 |
| T1.5 – BL | 21.8420 | 8.7173 | 4.3722 | 39.3118 |
| T2 – BL | 25.8655 | 8.6036 | 8.6236 | 43.1074 |
| T2.5 – BL | 29.5700 | 8.9575 | 11.6188 | 47.5211 |
| T3 – BL | 28.8009 | 8.5410 | 11.6843 | 45.9175 |
| T3.5 – BL | 25.1126 | 9.3804 | 6.3138 | 43.9114 |
| T4 – BL | 33.3703 | 8.9973 | 15.3394 | 51.4012 |
| T5 – BL | 28.2789 | 8.6981 | 10.8475 | 45.7103 |
| T6 – BL | 27.1375 | 9.4836 | 8.1319 | 46.1432 |
| F - M | -5.5080 | 33.5118 | -72.6671 | 61.6511 |
| Con | -0.1299 | 0.1579 | -0.4465 | 0.1866 |
| Body Weight | -1.3075 | 1.5588 | -4.4314 | 1.8163 |
| (T0 - BL) ✻ (F - M) | 19.7426 | 16.8326 | -13.9906 | 53.4759 |
| (T0.5 - BL) ✻ (F - M) | 16.8333 | 16.8540 | -16.9429 | 50.6095 |
| (T1 - BL) ✻ (F - M) | 8.3140 | 16.8409 | -25.4359 | 42.0639 |
| (T1.5 - BL) ✻ (F - M) | 5.4652 | 17.0317 | -28.6672 | 39.5975 |
| (T2 - BL) ✻ (F - M) | 13.3781 | 16.9428 | -20.5759 | 47.3321 |
| (T2.5 - BL) ✻ (F - M) | 9.6561 | 16.9275 | -24.2673 | 43.5795 |
| (T3 - BL) ✻ (F - M) | 23.0539 | 16.8329 | -10.6800 | 56.7877 |
| (T3.5 - BL) ✻ (F - M) | 26.3004 | 17.5056 | -8.7817 | 61.3825 |
| (T4 - BL) ✻ (F - M) | 34.1943 | 17.1654 | -0.2058 | 68.5944 |
| (T5 - BL) ✻ (F - M) | 32.3669 | 16.9158 | -1.5332 | 66.2670 |
| (T6 - BL) ✻ (F - M) | 19.8219 | 17.5303 | -15.3096 | 54.9534 |

*Note.* Dark grey shaded cells represent the 95% confidence intervals (CI) associated with timepoints significantly different from BL. Light grey shaded cells represent the 95% CI associated with timepoints during which a sex × timepoint interaction was observed. SE = standard error.
