# Supplementary material for: A six-hour time series of the acute effects of ingested cannabis intoxication on a battery of cognitive-motor tasks
Source: PLoS One. 2026 Aug 13;21(8):e0353389. doi: 10.1371/journal.pone.0353389 (PMC13472365; doi:10.1371/journal.pone.0353389)
Supplement: Supplementary file 7 [file pone.0353389.s007.docx]

**Reviewer #1:**

*Thank you for taking the time to review our manuscript. We greatly appreciate your feedback, which has led to an improved manuscript. Below, we address each of your comments and suggestions. To help identify the location of new text, we have added page numbers to the revised manuscript and refer to those in our responses (rather than the page numbers of the PDF generated by the Editorial Manager website, which is likely to include several pages before the actual manuscript).*

**1. Sample Size and Power**

a) The total number of participants (n=15) is small, and only 7 completed the control session. This limits statistical power and generalizability.

*Response: We agree with the reviewer, which is why we addressed the issue of the sample size for the control group in the “Considerations” subsection within the Discussion. Specifically, we pointed out that it reduced the power of our comparisons, which incorporated the control data into the statistical models (page 20, lines 1-5). It was not anticipated that our measures would vary meaningfully across time, which is why the control group was only a subset of participants. Nevertheless, by including these data in the statistical analyses, it allowed for us to account somewhat for potential fluctuations in cognitive function due to circadian rhythms, hunger, mental fatigue, etc.*

b) Suggest including a post-hoc power analysis or at least discussing potential Type II error risk, especially for the non-significant findings and sex comparisons.

*Response: In the “Considerations” subsection, we have edited the statement about sex-based differences to highlight the potential for type II error (page 19, lines 9-10).*

**2.Sex-Based Differences**
a) The manuscript notes sex × time interactions, but these are not deeply explored. Since sex differences are increasingly emphasized in psychopharmacology, a more detailed discussion about biological mechanisms (e.g., hormonal fluctuations, body composition differences, cannabinoid receptor expression) would strengthen this section.

*Response: In recognition of the historical underrepresentation of females in studies of human physiology, we intentionally balanced the sexes as closely as possible for an odd number of participants. However, the study was not designed specifically to evaluate the possibility of sex-based differences, which means we do not feel it is appropriate to deeply explore the interactions for the present sample size. In response to the reviewer’s comment, we expanded the text of the “Future Directions” subsection to emphasize the importance of investigating if the acute effects of cannabis are influenced by biological sex (page 20, lines 14-18).*

**3. Control Session and Learning Effects**

a) Only half the participants completed the control session, and no randomization is mentioned for order.

*Response: It is stated in the first paragraph of the “Familiarization, experimental, and control sessions” subsection of the Methods (page 6, lines 2-3) that, of the seven participants to take part in both sessions, four completed the control one first, which was the best counterbalancing possible for an odd number of participants.*

b) Clarify whether the partial control dataset could have introduced order or learning biases and justify why full counterbalancing was not feasible.
*Response: As stated above, the subset of the full group that participated in both sessions were pseudorandomized to participate in the control (n=4) or experimental session (n=3) first, effectively counterbalancing the sessions. This counterbalancing, alongside the familiarization session that each participant completed, helped control for order and learning effects within this group.*

**4. Ecological Validity vs. Experimental Control**

a) The study emphasizes ecological validity (e.g., allowing eating, non-standardized activities), but this might have introduced confounders.

*Response: Gastric emptying typically occurs within 2h (Liu et al., 2021, Crit Rev Food Sci Nutr), therefore eating after the completion of the 2-h timepoint (realistically no earlier than 2h 20min following THC administration), should not have interfered with the initial digestion of the capsule. However, differences between participants’ eating throughout the rest of the study could indeed lead to variation in the subsequent phases of THC metabolism (Peng and Shahidi, 2021, J Agric Food Chem), potentially contributing to some of the variability we observed. That being said, as* *increased hunger is a common effect of acute cannabis intoxication, we wanted to maintain participant comfort by allowing them to eat. Activities between testing trials were limited to primarily sedentary, low-cognitive arousal behaviours, so as to limit the potential of inducing physical or mental fatigue, anxiety or other high-emotional states.*

b) Recommend acknowledging this limitation more explicitly and perhaps quantifying its possible impact on HR/BP or cognitive results.

*Response: We have added text to the “Considerations” subsection to highlight that allowing participants to eat could have influenced the cardiovascular data (last four lines of page 19 and first line of page 20).*

**5. Dose Justification**

a) The use of 10 mg THC is justified as “ecologically valid,” but the rationale could be strengthened with pharmacokinetic data showing typical plasma THC levels for this dose range.

*Response: We have included details from the study by Vandrey and colleagues (reference 6) about plasma THC levels following ingestion of an edible with 10mg of THC (page 15, lines 10-14).*

b) Including THC blood concentration references would add context to interpret the 1–6 h effect window.

*Response: As stated in the preceding response, we expanded on our reference to the work by Vandrey and colleagues to include values for THC blood concentrations. Furthermore, we make specific reference to the timing of our testing window.*

**6. Statistical Reporting**

a) While the use of linear mixed models is commendable, confidence intervals alone are presented without effect sizes.

*Response: A linear mixed-effects model does not generate effect sizes for the model itself; however, we were able to obtain effect sizes (ηp²) from a corrected one-way, repeated measures analysis of variance (ANOVA) associated with each linear mixed-effects model. These values were added throughout the Results section.*

b) Reporting standardized effect sizes (e.g., Cohen’s d or partial η²) would better convey the magnitude of changes in reaction time and accuracy.

*Response: Please see the response to the preceding comment.*

**7. Interpretation of Correlation Between Subjective and Objective Measures**
a) The correlation between perceived intoxication and reaction time is intriguing.

*Response: We agree. It is exciting to find a significant correlation between subjective levels of cannabis intoxication and an objective measure of cognitive function.*

b) Suggest expanding on the neurophysiological mechanisms linking subjective intoxication and motor response latency.

*Response: Due to the nature of our experiment, we are not able to identify the direct neurophysiological mechanisms causing our results. We now specify this at the end of the “Subjective measures” subsection of the Discussion, but also speculate that different actions of THC may be responsible for the subjective and objective effects (page 16, lines 8-14).*

**Reviewer #2:** I have read and included the edits for the article "A six-hour time series of the acute effects of ingested cannabis intoxication on a battery of cognitive-motor tasks" on the attached file. Please review and make the necessary comments/changes.

*Response: Thank you for the thoughtful comments to improve our manuscript. Please see below for our response to each comment. To help identify the location of new text, we have added page numbers to the revised manuscript and refer to those in our responses (rather than the page numbers of the PDF generated by the Editorial Manager website, which is likely to include several pages before the actual manuscript).*

**Methods:**

Limitations of this study include-

1) Very small sample size, that decreases the power and generalizability of the study

*Response: Certainly, more participants could have added to the generalizability of the study; however, our sample size is similar to other recent studies examining the effects of acute cannabis use (e.g., references 5 and 36). Additionally, we are powered for most of our comparisons, as many significant differences were observed. Future work will hopefully build off this initial study to increase generalizability and investigate these effects in different populations.*

2) Lack of blinding of the participants

*Response: While a lack of blinding could have led to bias from the participants in their subjective and objective results, only a subset of the experimental group participated in the control session, and therefore blinding was not possible for approximately half of the participants.*

3) Only 7 of the 15 participants appear to have completed the control or no-intervention session,

*Response: It was not anticipated that our measures would vary meaningfully across time, which is why the control group was only a subset of participants. Nevertheless, by including these data in the statistical analyses, it allowed for us to account somewhat for potential fluctuations in cognitive function due to circadian rhythms, hunger, mental fatigue, etc. We addressed the issue of the sample size for the control group in the “Considerations” subsection of the Discussion. Specifically, we pointed out that it reduced the power of our comparisons, which incorporated the control data into the statistical models (page 20, lines 1-5).*

4) Were potential confounding variables addressed- including Caffeine use, Alcohol and other comorbid drug use.,

*Response: We recorded each participant’s average daily caffeine intake, weekly alcohol use, and other recreational drug use within the year prior to their familiarization session. To maintain ecological validity and prevent any potential caffeine withdrawal-induced changes in cognitive function (e.g., Rogers et al., 2005, 2013, Psychopharmacology; Yeomans et al., 2002, Psychopharmacology), we had participants adhere to their regular caffeine intake on the day(s) of testing.*

5) It was a small demographic spread which includes participants that were in their late 20s and early 30s, comparatively healthy. This reduces real world applicability to older patients, patients with medical conditions, diverse socioeconomic and cognitive backgrounds.

*Response: While the homogeneity of our sample does reduce generalizability to specific populations, our purpose was to understand the time series of intoxication in the absence of potential confounders such as older age, specific clinical diagnoses, altered cognitive function, etc. It is our hope that the current study can inform future work to investigate the effects of acute cannabis use among particular populations of interest, as many different demographic groups consume cannabis. Additionally, because we were not aiming to characterize specific physiological mechanisms behind cannabis intoxication, how particular populations may be differently impacted by this intoxication, or the sociocultural factors contributing to cannabis use, we view our sample as appropriate for the research question.*

Restricting enrollment to individuals who use cannabis infrequently would help in reducing tolerance effects and increasing the sensitivity to acute impairment. This improves the internal validity regarding the effects of a single intoxicating dose (Lines 81-83)

*Response: Indeed, thank you. We are aware of the well-documented neuroanatomical and behavioural adaptations that occur with chronic cannabis use, and we wanted to avoid that as a confounding factor for this initial study. Future work can hopefully aim to understand this time series in individuals who use cannabis more regularly.*

**Cannabis Product:**

1) The 10mg THC dosing used here would be too low for real world generalizability- Its much lower than commercial edibles available in the USA (usually 20-50 mg units) and other global markets.

*Response: Although doses of THC >10mg are certainly consumed, especially in those who use cannabis regularly and have likely developed significant tolerance to the drug, 10mg is still a relevant dose for the general population in Canada and elsewhere. For example, legislation in Washington state requires infused cannabis products (edibles and other ingestibles) to not exceed a single dose of more than 10mg Δ9-THC (*[*https://app.leg.wa.gov/wac/default.aspx?cite=314-55-095*](https://app.leg.wa.gov/wac/default.aspx?cite=314-55-095)*). Additionally, previous literature investigating acute edible cannabis intoxication in a population similar to that of the current study (i.e., infrequent, but not totally naïve, cannabis users), 10mg of THC was sufficient to induce signs and symptoms of intoxication while avoiding common adverse effects associated with higher doses of THC, such as vomiting or hallucinations (25mg used in reference 5), or overall negative experiences, including feelings of severe anxiety and heart racing (20-25mg used in references 6 and 21).*

2) Also no CBD or other Cannabinoids were included in the study - most commercial products contain a mix of THC + CBD, and CBD moderates some of the psychoactive effects of THC.

*Response: It is true that most commercial products contain a mix of both primary cannabinoids, and the majority of cannabis used in Canada includes both as well. In the current study, we were focused on characterizing the timeline of intoxication from solely THC in order to accurately plan future studies during this “intoxication window”. We will have several forthcoming papers from a set of recently-completed experiments that investigated the effects of THC and CBD, both alone and in combination, on various subjective measures as well as a variety of cognitive-motor and sensorimotor tasks.*

*With respect to your last point, based on a preliminary analysis of data from our recent set of experiments, CBD may not temper the effects of THC on sensorimotor function as much as much expected based on their interaction and modulation of CB1r activity.*

3) Softgel edibles have a delayed and highly variable absorption.

*Response: We aimed to limit variability in the digestion/absorption by preventing participants from eating for the first 2h following drug administration. However, interindividual differences would persist, causing some variability in the overall absorption of THC, which likely explains some of the variability in our data. Unfortunately, there is potential for significant differences in total drug uptake regardless of route of administration (smoking, vaping, edible, etc.), which highlights the need for more work in this area to get a more global understanding of how cannabis affects human behaviour.*

4) Single dose design limits understanding of dose response effects

*Response: As our aim was to characterize the time course of intoxication following ingestion of an edible cannabis product to try to identify an ideal testing window for future studies, we chose a single dose design. Some previous work (reference 6; Spindle et al., 2020, J Anal Toxicol) has compared dose responses, and the time to reach peak serum THC concentration and subjective drug effects are very similar across a range of doses from 10mg-50mg. Further, as 10mg THC is a commonly used edible dose in Canada and other areas, while simultaneously being low enough so as to limit adverse events, we chose this dose for the current study. Future work will certainly consider dose-response effects.*

5) No comparison here to inhaled cannabis

*Response: The timeline of intoxication has been explored previously in the context of inhaled cannabis (e.g., reference 5; Kurzthaler et al., 1999, J Clin Psychiatry; Ramaekers et al. 2006, Drug Alcohol Depend; reviewed by Crean et al., 2011, J Addict Med); however, with the rise in popularity of ingested cannabis, we chose to focus on advancing our understanding of the effects of an edible cannabis product. Further, considering the adverse health implications of smoking and vaping, we chose not to include an inhaled cannabis condition in our protocol.*

**Study Design:**

1) Timing of the sessions can confound with the diurnal cognitive fluctuation where cognitive performance naturally changes across the day due to mental fatigue, circadian rhythm, Hunger etc.

*Response: The subset control group allowed us a within-subject comparison to control for potential daily fluctuations in cognitive function due to circadian rhythms, hunger, mental fatigue, etc. Individuals who completed both sessions began each at the same time of day, partook in the same activities between trials, and ate similar types and amount of food throughout the sessions.*

2) The food control may introduce variability- by subjects being forced to fast until 2 hours post ingestion and then being allowed to eat freely.

*Response: Gastric emptying typically occurs within 2h (Liu et al., 2021, Crit Rev Food Sci Nutr), therefore eating after the completion of the 2-h timepoint (realistically no earlier than 2h 20min following THC administration), should not have interfered with the initial digestion of the capsule. However, differences between participants’ eating throughout the rest of the study could indeed lead to variation in the subsequent phases of THC metabolism (Peng and Shahidi, 2021, J Agric Food Chem), potentially contributing to some of the variability we observed. That being said, as increased hunger is a common effect of acute cannabis intoxication, we wanted to maintain participant comfort by allowing them to eat.*

3) testing blocks began immediately after ingestion- THC can take 30-120 mins to have onset of action.

*Response: It was the aim of this study to characterize the time course of effects caused by THC ingestion; hence, it was essential to capture the onset. By starting testing blocks immediately following THC administration, we are able to collect a timepoint after ingestion but before any subjective or objective drug effects could possibly occur. Moreover, by testing at 30-min intervals, we increased our ability to detect the onset and peak of intoxication. Previous studies investigating acute effects of cannabis edibles typically took measurements every hour following ingestion (e.g., reference 8) or used a single pre-post design (e.g., reference 21).*
