# Supplementary material for: A six-hour time series of the acute effects of ingested cannabis intoxication on a battery of cognitive-motor tasks
Source: PLoS One. 2026 Aug 13;21(8):e0353389. doi: 10.1371/journal.pone.0353389 (PMC13472365; doi:10.1371/journal.pone.0353389)
Supplement: Supplementary file 9 [file pone.0353389.s009.docx]

The authors would like to thank each of the reviewers for their helpful and insightful comments. We believe that your contributions have improved the quality of the manuscript. Please find our responses to your feedback below. Page and line numbers refer to the marked-up version of the manuscript.

Reviewer #2:

Thank you for all the edits you made in response to comments made on "A six-hour time series of the acute effects of ingested cannabis intoxication on a battery of cognitive-motor tasks"

*Response: We thank reviewer # 2 for their help in making the present article better.*

Reviewer #3:

This manuscript describes an acute dosing experiment of ingested THC with the aim of establishing a timeline of behavioural, subjective, and neurocognitive effects over 6 hours. Participants demonstrated increased subjective drug effects, heart rate and blood pressure, and impaired performance on three measures of cognitive-motor function, with small to medium effect sizes. The timeline of effects is consistent with results from previous acute dosing studies as well as previous pharmacokinetic data on ingested THC.

Some specific strengths of the study include the standardised fasting period to reduce variability in drug absorption, the numerous testing blocks, the well-controlled study conditions used to reduce bias on cognitive and subjective tests, and the statistical comparison of results for female and male participants, an important consideration for cannabinoid research. Major limitations include the limited number of participants taking part in the control session and the lack of blinding.

The following is a list of the considerations for the authors. I have begun with a single, most critical issue that I wish the authors to consider, before listing the more minor issues and questions in the order in which they appear in the text:

1) While the lack of blinding was unavoidable due to the study design, it is still a major limitation and must be discussed in the Considerations section as such. The anticipatory effect for cannabinoids is known to be very strong, as demonstrated by Cami et al. 1991.

Camí J, Guerra D, Ugena B, et al. (1991) Effect of subject expectancy on the THC intoxication and disposition from smoked hashish cigarettes. Pharmacology Biochemistry and Behavior 40(1): 115–119.

*Response: We have added a section to the Considerations section (bottom of page 20) to discuss the lack of blinding as a potential limitation.*

Minor Issues and comments-

Methods:

2) Please present in the manuscript or supplementary materials the recorded data on participants’ caffeine, alcohol, smoking, and other drug use. Were participants requested to refrain from cannabis, alcohol, and/or other drug use either before each session or over the entire testing period, and were biological tests (e.g. urine) used to confirm this? Please also state in the manuscript whether or not participants were allowed to smoke during the testing sessions.

*Response: We have added a passage describing the history of other psychoactive substance use among our sample (bottom of page 5 and top of page 6), and data about caffeine consumption has been added to Table 1. Neither alcohol usage habits nor smoking habits (cannabis or otherwise) were recorded for our sample. Participants were asked to abstain from consuming cannabis or other psychoactive substances (>1 week) as well as alcohol (>12 hours) prior to a testing session. No tests were done to confirm adherence to these abstinence protocols, participants were taken at their word. This information has been added to the Methods section (page 7, lines 5-9). Furthermore, we now state in the manuscript that participants were not allowed to smoke during testing, and that no one expressed a desire to do so (page 7, lines 9-10).*

3) Please provide the following information in the manuscript RE the cannabis product:

a. Was the THC synthetic or a plant extract?

b. What were the main excipients in the product?

c. Did the product contain any CBD at all, and if so how much? Also, if this information is available, what were the amounts of other active cannabinoids (e.g., CBG) in the product?

*Response: a) The THC was a plant extract. b) The excipients are* *medium chain triglycerides oil, gelatin, water, glycerine, Tartrazine Lake (a dye), and Brilliant Blue FCF (a dye). c) The product is sold as having no active cannabinoids apart from THC. However, despite the intention of 0mg CBD, the company specifications state that each capsule may contain up to 1mg CBD (compared to 4-6mg THC). Considering that the product is a plant extract and that the company cannot rule out the presence of CBD, it seems likely to us that the capsules may contain traces of other cannabinoids present in the plant. However, we can neither confirm nor deny the presence of other cannabinoids. This information has been added to the Methods section (page 8, lines 2-7).*

Discussion - Considerations:

4) The lack of standardisation of the food after the 2-hour timepoint is a minor limitation and should be at least briefly mentioned in the manuscript.

*Response: This minor limitation is now noted in the Considerations section (bottom of page 21).*

5) Female participants used cannabis 7-8x more frequently than males on average (8.1 days/year vs 1.1). Though 8.1 days per year still constitutes infrequent use, the increased familiarity with cannabis in the female group, as well as the small sample sizes in both groups, could feasibly have affected the results for this particular analysis. This should be acknowledged in the limitations.

*Response: This is a valid observation, and we have added this point to the Considerations section (page 21, lines 3-7).*

Tables and figures:

6) A very small comment – In supplementary table 3, diastolic blood pressure, there appears to be a highlight missing for the (T5 – BL) x (F – M) row.

*Response: Thank you for catching this mistake. We have highlighted the noted row in Supplementary Table 2 to denote a significant difference.*

Reviewer #4:

I commend the authors for their meticulous work and the intention to expand the literature on the effects of orally ingested cannabis/THC. The manuscript is generally well written and structured. However, the main limitations of the study is a small sample size and lack of proper placebo control and randomisation, which is common in the literature. It is therefore difficult to generalize the results of this study to other studies of oral cannabis/THC. My specific comments are below.

1. For clarity and consistency, it would be preferable to report the THC dose as a single value (e.g., “10 mg”) rather than “2 × 5 mg.”

*Response: We have removed the mention of the two 5-mg capsules from the Abstract (page 2, line 8), but, for the sake of accuracy, retained that information in the Methods, while clarifying that the total intervention dosage was 10mg (bottom of page 7).*

2. The abstract does not clearly indicate whether a placebo condition was included.

*Response: We have added a line to the Abstract detailing the control session (page 2, lines 8-10)*

3. The statement, “Of the few studies to systematically investigate the acute effects of cannabis, most had participants smoke the drug,” is misleading. There is an extensive literature examining the acute effects of THC and cannabis administered via multiple routes, including oral, inhaled, and intravenous administration. The introduction would benefit from a more comprehensive and balanced overview of this literature.

*Response: Thank you for highlighting this overreaching statement; it has been removed from the manuscript as we agree that there is a body of work related to each of the noted routes of consumption. In the previous version of the manuscript, the second paragraph of the Introduction focused primarily on studies that examined the influence of inhaled cannabis on cognitive-motor performance; however, in response to this comment and the next, we have expanded this section of the Introduction to include more studies with oral THC (bottom of page 3 and top of page 4).*

4. Relatedly, the introduction could be strengthened by referencing prior studies examining the effects of oral THC on cognitive-motor performance such as studies using driving-related outcomes, as this provides context to the present work.

*Response: As noted in our preceding response, we have expanded the discussion of cognitive-motor results with oral THC, including in the context of driving (page 3, lines 17-19).*

5. The manuscript should state that Δ9-THC is the “main” psychoactive compound in cannabis, as cannabis contain other cannabinoids which are agonists at the CB1 receptor and capable of exerting psychoactive effects.

*Response: As requested, we have clarified this by adding the word “main” (page 3, line 7).*

6. The claim that “to the best of our knowledge, no study has evaluated how cognitive-motor performance is affected by ingestion of a commercially available edible THC product, with measurements taken more than once per hour, nor considered possible effects of biological sex” appears to be overstated. Several prior studies have examined repeated post-dose assessments following oral THC and/or have considered sex effects.

*Response: You are correct that this statement overreached. We have removed this sentence.*

7. The sentence beginning “Compared to inhalation, edibles are an appealing method of cannabis consumption to use in research because…” is confusing and potentially inaccurate. While edibles may offer a prolonged intoxication period, THC and cannabinoid dosing is generally more precisely controlled with inhaled or intravenous administration due to more predictable bioavailability.

*Response: We appreciate your concerns about this claim. However, based on the results of several bioavailability studies concerning both smoked and ingested THC, the review by Huestis et al. (2007) reported that the bioavailability from smoking was ~2-56%, whereas the oral route offered a bioavailability of ~4-20%. Thus, oral ingestion offers less variable bioavailability, though the maximal bioavailability is lower for ingestion compared to smoking. Concerning accuracy in dosing, we are confident that all of the THC purported to be contained within the product enters the body when a gel capsule is ingested; however, we do not have the same confidence with smoked products, as some of the THC may undergo combustion or other chemical alteration before entering the body, and not all smoke may be inhaled even under ideal circumstances (e.g., when a participant is asked to inhale all the contents of a balloon, some smoke/residue may be left in the balloon or escape the participant’s mouth). Additionally, while intravenous administration would avoid these issues, due to its invasiveness, this is an extremely rare method of recreational/medical consumption (making it not ecologically valid). We have clarified the cited statement with the details we have provided in this response (page 4, lines 5-8). We have also edited the beginning of the sentence to clarify that maximal bioavailability for ingested THC is lower than for smoked THC.*

8. It is unclear whether participants underwent drug screening (e.g., urine toxicology) and pregnancy testing at each visit. Most acute THC studies include such procedures, and their absence or inclusion should be stated.

*Response: No tests were done to confirm adherence to abstinence protocols related to drug and alcohol use prior to testing sessions as participants were taken at their word. This information has been added to the Methods section (page 7, lines 5-9). Each participant who identified themselves as having child-bearing potential was given a take-home pregnancy test and reported the result prior to the intervention session. Thank you for catching this accidental omission; text has been to the Methods section (page 5, lines 19-21).*

9. The Methods section would benefit from a more comprehensive description of inclusion and exclusion criteria to allow readers to better assess participant selection and generalizability.

*Response: We have expanded upon the inclusion/exclusion criteria section (page 5, lines 10-19).*

10. While the manuscript states that participants were asked to fast between baseline and 2h post-ingestion, there is no information as to what instructions the participants were given prior to baseline. This is important as fasting ingestion of oral THC has a much sooner onset of effects than that of fed states.

*Response: Participants were instructed to consume their standard breakfast before participation to: a) not burden the participants with a potentially onerous fasting regimen prior to participation, and b) to increase the ecological validity of the study. We have added this information to the Methods section (page 9, lines 8-9). We have also added text related to this point in the Considerations section (bottom of page 21).*

11. While the discussion references plasma THC concentrations from other studies using similar doses, such comparisons should be interpreted cautiously. Differences in formulation, fed versus fasted state, participant characteristics, and other factors can substantially influence absorption and pharmacokinetics, limiting the direct translatability of these comparisons.

*Response: You make an accurate observation. We have clarified and softened our statements concerning comparisons between different reports on ingested cannabis products (page 17, lines 2-4).*
