# Supplementary material for: A six-hour time series of the acute effects of ingested cannabis intoxication on a battery of cognitive-motor tasks
Source: PLoS One. 2026 Aug 13;21(8):e0353389. doi: 10.1371/journal.pone.0353389 (PMC13472365; doi:10.1371/journal.pone.0353389)
Supplement: Peer review history

# Peer review: Decision Letter 0

**DOI:** 10.1371/journal.pone.0353389.r001

**Authors:** Souparno Mitra

26 Nov 2025

Dear Dr. McNeil,

Thank you for submitting your manuscript to PLOS ONE. After careful consideration, we feel that it has merit but does not fully meet PLOS ONE’s publication criteria as it currently stands. Therefore, we invite you to submit a revised version of the manuscript that addresses the points raised during the review process.

==============================

**ACADEMIC EDITOR:** **Please address the reviewer comments and resubmit for reconsideration for publication.**

==============================

Please submit your revised manuscript by Jan 10 2026 11:59PM. If you will need more time than this to complete your revisions, please reply to this message or contact the journal office at plosone@plos.org. When you're ready to submit your revision, log on to https://www.editorialmanager.com/pone/ and select the 'Submissions Needing Revision' folder to locate your manuscript file.

- A rebuttal letter that responds to each point raised by the academic editor and reviewer(s). You should upload this letter as a separate file labeled 'Response to Reviewers'.
- A marked-up copy of your manuscript that highlights changes made to the original version. You should upload this as a separate file labeled 'Revised Manuscript with Track Changes'.
- An unmarked version of your revised paper without tracked changes. You should upload this as a separate file labeled 'Manuscript'.

If you would like to make changes to your financial disclosure, please include your updated statement in your cover letter. Guidelines for resubmitting your figure files are available below the reviewer comments at the end of this letter.

If applicable, we recommend that you deposit your laboratory protocols in protocols.io to enhance the reproducibility of your results. Protocols.io assigns your protocol its own identifier (DOI) so that it can be cited independently in the future. For instructions see: https://journals.plos.org/plosone/s/submission-guidelines#loc-laboratory-protocols. Additionally, PLOS ONE offers an option for publishing peer-reviewed Lab Protocol articles, which describe protocols hosted on protocols.io. Read more information on sharing protocols at https://plos.org/protocols?utm_medium=editorial-email&utm_source=authorletters&utm_campaign=protocols.

We look forward to receiving your revised manuscript.

Kind regards,

Souparno Mitra, M.D.

Academic Editor

PLOS ONE

Journal Requirements:

When submitting your revision, we need you to address these additional requirements.

1. Please ensure that your manuscript meets PLOS ONE's style requirements, including those for file naming. The PLOS ONE style templates can be found at

https://journals.plos.org/plosone/s/file?id=wjVg/PLOSOne_formatting_sample_main_body.pdf and

https://journals.plos.org/plosone/s/file?id=ba62/PLOSOne_formatting_sample_title_authors_affiliations.pdf

2. We note that the grant information you provided in the ‘Funding Information’ and ‘Financial Disclosure’ sections do not match.

When you resubmit, please ensure that you provide the correct grant numbers for the awards you received for your study in the ‘Funding Information’ section.

3. Thank you for stating the following financial disclosure:

This work was supported by the Natural Sciences and Engineering Research Council of Canada under DG grant 5018423-2020 (C.J.M) and the Stober Foundation Health Fund (C.J.M. & B.H.D.). The funding bodies had no decision-making role in the study design, data collection or analysis, decision to publish, or preparation of the manuscript.

Please state what role the funders took in the study. If the funders had no role, please state: "The funders had no role in study design, data collection and analysis, decision to publish, or preparation of the manuscript."

If this statement is not correct you must amend it as needed.

Please include this amended Role of Funder statement in your cover letter; we will change the online submission form on your behalf.

4. Thank you for stating the following in the Competing Interests section:

The authors declare there are no competing interests, financial or otherwise.

Please confirm that this does not alter your adherence to all PLOS ONE policies on sharing data and materials, by including the following statement: "This does not alter our adherence to PLOS ONE policies on sharing data and materials.” (as detailed online in our guide for authors http://journals.plos.org/plosone/s/competing-interests). If there are restrictions on sharing of data and/or materials, please state these. Please note that we cannot proceed with consideration of your article until this information has been declared.

Please include your updated Competing Interests statement in your cover letter; we will change the online submission form on your behalf.

5. When completing the data availability statement of the submission form, you indicated that you will make your data available on acceptance. We strongly recommend all authors decide on a data sharing plan before acceptance, as the process can be lengthy and hold up publication timelines. Please note that, though access restrictions are acceptable now, your entire data will need to be made freely accessible if your manuscript is accepted for publication. This policy applies to all data except where public deposition would breach compliance with the protocol approved by your research ethics board. If you are unable to adhere to our open data policy, please kindly revise your statement to explain your reasoning and we will seek the editor's input on an exemption. Please be assured that, once you have provided your new statement, the assessment of your exemption will not hold up the peer review process.

6. Please upload a copy of Figure 6, to which you refer in your text on page 13. If the figure is no longer to be included as part of the submission please remove all reference to it within the text.

7. Please include captions for your Supporting Information files at the end of your manuscript, and update any in-text citations to match accordingly. Please see our Supporting Information guidelines for more information: http://journals.plos.org/plosone/s/supporting-information.

8. If the reviewer comments include a recommendation to cite specific previously published works, please review and evaluate these publications to determine whether they are relevant and should be cited. There is no requirement to cite these works unless the editor has indicated otherwise.

[Note: HTML markup is below. Please do not edit.]

Reviewers' comments:

Reviewer's Responses to Questions

**Comments to the Author**

1. Is the manuscript technically sound, and do the data support the conclusions?

Reviewer #1: Yes

Reviewer #2: Yes

**********

2. Has the statistical analysis been performed appropriately and rigorously?

Reviewer #1: Yes

Reviewer #2: I Don't Know

**********

3. Have the authors made all data underlying the findings in their manuscript fully available?

The PLOS Data policy

Reviewer #1: Yes

Reviewer #2: Yes

**********

4. Is the manuscript presented in an intelligible fashion and written in standard English?

Reviewer #1: Yes

Reviewer #2: Yes

**********

Reviewer #1: 1.Sample Size and Power

a) The total number of participants (n=15) is small, and only 7 completed the control session. This limits statistical power and generalizability.

b) Suggest including a post-hoc power analysis or at least discussing potential Type II error risk, especially for the non-significant findings and sex comparisons.

2.Sex-Based Differences

a) The manuscript notes sex × time interactions, but these are not deeply explored.

b) Since sex differences are increasingly emphasized in psychopharmacology, a more detailed discussion about biological mechanisms (e.g., hormonal fluctuations, body composition differences, cannabinoid receptor expression) would strengthen this section.

3.Control Session and Learning Effects

a) Only half the participants completed the control session, and no randomization is mentioned for order.

b) Clarify whether the partial control dataset could have introduced order or learning biases and justify why full counterbalancing was not feasible.

4.Ecological Validity vs. Experimental Control

a) The study emphasizes ecological validity (e.g., allowing eating, non-standardized activities), but this might have introduced confounders.

b) Recommend acknowledging this limitation more explicitly and perhaps quantifying its possible impact on HR/BP or cognitive results.

5.Dose Justification

a) The use of 10 mg THC is justified as “ecologically valid,” but the rationale could be strengthened with pharmacokinetic data showing typical plasma THC levels for this dose range.

b) Including THC blood concentration references would add context to interpret the 1–6 h effect window.

6.Statistical Reporting

a) While the use of linear mixed models is commendable, confidence intervals alone are presented without effect sizes.

b) Reporting standardized effect sizes (e.g., Cohen’s d or partial η²) would better convey the magnitude of changes in reaction time and accuracy.

7. Interpretation of Correlation Between Subjective and Objective Measures

a) The correlation between perceived intoxication and reaction time is intriguing.

b) Suggest expanding on the neurophysiological mechanisms linking subjective intoxication and motor response latency.

Reviewer #2: I have read and included the edits for the article "A six-hour time series of the acute effects of ingested cannabis intoxication on a battery of cognitive-motor tasks" on the attached file. Please review and make the necessary comments/changes.

**********

what does this mean?). If published, this will include your full peer review and any attached files.

If you choose “no”, your identity will remain anonymous but your review may still be made public.

**Do you want your identity to be public for this peer review?**  For information about this choice, including consent withdrawal, please see our Privacy Policy

Reviewer #1: No

Reviewer #2: No

**********

[NOTE: If reviewer comments were submitted as an attachment file, they will be attached to this email and accessible via the submission site. Please log into your account, locate the manuscript record, and check for the action link "View Attachments". If this link does not appear, there are no attachment files.]

To ensure your figures meet our technical requirements, please review our figure guidelines: https://journals.plos.org/plosone/s/figures

You may also use PLOS’s free figure tool, NAAS, to help you prepare publication quality figures: https://journals.plos.org/plosone/s/figures#loc-tools-for-figure-preparation.

NAAS will assess whether your figures meet our technical requirements by comparing each figure against our figure specifications.

Attachment Submitted filename: Completed- PONE-D-25-56417_reviewer.pdf

# Author comment: Author response to Decision Letter 1

**DOI:** 10.1371/journal.pone.0353389.r002

3 Jan 2026

Please see the uploaded document entitled, "Response to reviewers".

Attachment Submitted filename: Response to reviewers.docx

# Peer review: Decision Letter 1

**DOI:** 10.1371/journal.pone.0353389.r003

**Authors:** Souparno Mitra

28 Jan 2026

Dear Dr. McNeil,

Thank you for submitting your manuscript to PLOS ONE. After careful consideration, we feel that it has merit but does not fully meet PLOS ONE’s publication criteria as it currently stands. Therefore, we invite you to submit a revised version of the manuscript that addresses the points raised during the review process.

Please submit your revised manuscript by Mar 14 2026 11:59PM. If you will need more time than this to complete your revisions, please reply to this message or contact the journal office at plosone@plos.org. When you're ready to submit your revision, log on to https://www.editorialmanager.com/pone/ and select the 'Submissions Needing Revision' folder to locate your manuscript file.

- A letter that responds to each point raised by the academic editor and reviewer(s). You should upload this letter as a separate file labeled 'Response to Reviewers'.
- A marked-up copy of your manuscript that highlights changes made to the original version. You should upload this as a separate file labeled 'Revised Manuscript with Track Changes'.
- An unmarked version of your revised paper without tracked changes. You should upload this as a separate file labeled 'Manuscript'.

If applicable, we recommend that you deposit your laboratory protocols in protocols.io to enhance the reproducibility of your results. Protocols.io assigns your protocol its own identifier (DOI) so that it can be cited independently in the future. For instructions see: https://journals.plos.org/plosone/s/submission-guidelines#loc-laboratory-protocols. Additionally, PLOS ONE offers an option for publishing peer-reviewed Lab Protocol articles, which describe protocols hosted on protocols.io. Read more information on sharing protocols at https://plos.org/protocols?utm_medium=editorial-email&utm_source=authorletters&utm_campaign=protocols.

We look forward to receiving your revised manuscript.

Kind regards,

Souparno Mitra, M.D.

Academic Editor

PLOS One

Journal Requirements:

If the reviewer comments include a recommendation to cite specific previously published works, please review and evaluate these publications to determine whether they are relevant and should be cited. There is no requirement to cite these works unless the editor has indicated otherwise.

Please review your reference list to ensure that it is complete and correct. If you have cited papers that have been retracted, please include the rationale for doing so in the manuscript text, or remove these references and replace them with relevant current references. Any changes to the reference list should be mentioned in the rebuttal letter that accompanies your revised manuscript. If you need to cite a retracted article, indicate the article’s retracted status in the References list and also include a citation and full reference for the retraction notice.

Additional Editor Comments

Please address the reviewer comments and resubmit for further consideration for acceptance.

[Note: HTML markup is below. Please do not edit.]

Reviewers' comments:

Reviewer's Responses to Questions

**Comments to the Author**

Reviewer #2: All comments have been addressed

Reviewer #3: (No Response)

Reviewer #4: (No Response)

**********

2. Is the manuscript technically sound, and do the data support the conclusions?

Reviewer #2: Yes

Reviewer #3: Yes

Reviewer #4: Partly

**********

3. Has the statistical analysis been performed appropriately and rigorously?

Reviewer #2: I Don't Know

Reviewer #3: Yes

Reviewer #4: I Don't Know

**********

4. Have the authors made all data underlying the findings in their manuscript fully available?

The PLOS Data policy

Reviewer #2: Yes

Reviewer #3: Yes

Reviewer #4: Yes

**********

5. Is the manuscript presented in an intelligible fashion and written in standard English?

Reviewer #2: Yes

Reviewer #3: Yes

Reviewer #4: Yes

**********

Reviewer #2: Thank you for all the edits you made in response to comments made on "A six-hour time series of the acute effects of ingested cannabis intoxication on a battery of cognitive-motor tasks"

Reviewer #3: This manuscript describes an acute dosing experiment of ingested THC with the aim of establishing a timeline of behavioural, subjective, and neurocognitive effects over 6 hours. Participants demonstrated increased subjective drug effects, heart rate and blood pressure, and impaired performance on three measures of cognitive-motor function, with small to medium effect sizes. The timeline of effects is consistent with results from previous acute dosing studies as well as previous pharmacokinetic data on ingested THC.

Some specific strengths of the study include the standardised fasting period to reduce variability in drug absorption, the numerous testing blocks, the well-controlled study conditions used to reduce bias on cognitive and subjective tests, and the statistical comparison of results for female and male participants, an important consideration for cannabinoid research. Major limitations include the limited number of participants taking part in the control session and the lack of blinding.

The following is a list of the considerations for the authors. I have begun with a single, most critical issue that I wish the authors to consider, before listing the more minor issues and questions in the order in which they appear in the text:

1) While the lack of blinding was unavoidable due to the study design, it is still a major limitation and must be discussed in the Considerations section as such. The anticipatory effect for cannabinoids is known to be very strong, as demonstrated by Cami et al. 1991.

Camí J, Guerra D, Ugena B, et al. (1991) Effect of subject expectancy on the THC intoxication and disposition from smoked hashish cigarettes. Pharmacology Biochemistry and Behavior 40(1): 115–119.

Minor Issues and comments-

Methods:

2) Please present in the manuscript or supplementary materials the recorded data on participants’ caffeine, alcohol, smoking, and other drug use. Were participants requested to refrain from cannabis, alcohol, and/or other drug use either before each session or over the entire testing period, and were biological tests (e.g. urine) used to confirm this? Please also state in the manuscript whether or not participants were allowed to smoke during the testing sessions.

3) Please provide the following information in the manuscript RE the cannabis product:

a. Was the THC synthetic or a plant extract?

b. What were the main excipients in the product?

c. Did the product contain any CBD at all, and if so how much? Also, if this information is available, what were the amounts of other active cannabinoids (e.g., CBG) in the product?

Discussion - Considerations:

4) The lack of standardisation of the food after the 2-hour timepoint is a minor limitation and should be at least briefly mentioned in the manuscript.

5) Female participants used cannabis 7-8x more frequently than males on average (8.1 days/year vs 1.1). Though 8.1 days per year still constitutes infrequent use, the increased familiarity with cannabis in the female group, as well as the small sample sizes in both groups, could feasibly have affected the results for this particular analysis. This should be acknowledged in the limitations.

Tables and figures:

6) A very small comment – In supplementary table 3, diastolic blood pressure, there appears to be a highlight missing for the (T5 – BL) x (F – M) row.

Reviewer #4: I commend the authors for their meticulous work and the intention to expand the literature on the effects of orally ingested cannabis/THC. The manuscript is generally well written and structured. However, the main limitations of the study is a small sample size and lack of proper placebo control and randomisation, which is common in the literature. It is therefore difficult to generalize the results of this study to other studies of oral cannabis/THC. My specific comments are below.

1. For clarity and consistency, it would be preferable to report the THC dose as a single value (e.g., “10 mg”) rather than “2 × 5 mg.”

2. The abstract does not clearly indicate whether a placebo condition was included.

3. The statement, “Of the few studies to systematically investigate the acute effects of cannabis, most had participants smoke the drug,” is misleading. There is an extensive literature examining the acute effects of THC and cannabis administered via multiple routes, including oral, inhaled, and intravenous administration. The introduction would benefit from a more comprehensive and balanced overview of this literature.

4. Relatedly, the introduction could be strengthened by referencing prior studies examining the effects of oral THC on cognitive-motor performance such as studies using driving-related outcomes, as this provides context to the present work.

5. The manuscript should state that Δ9-THC is the “main” psychoactive compound in cannabis, as cannabis contain other cannabinoids which are agonists at the CB1 receptor and capable of exerting psychoactive effects.

6. The claim that “to the best of our knowledge, no study has evaluated how cognitive-motor performance is affected by ingestion of a commercially available edible THC product, with measurements taken more than once per hour, nor considered possible effects of biological sex” appears to be overstated. Several prior studies have examined repeated post-dose assessments following oral THC and/or have considered sex effects.

7. The sentence beginning “Compared to inhalation, edibles are an appealing method of cannabis consumption to use in research because…” is confusing and potentially inaccurate. While edibles may offer a prolonged intoxication period, THC and cannabinoid dosing is generally more precisely controlled with inhaled or intravenous administration due to more predictable bioavailability.

8. It is unclear whether participants underwent drug screening (e.g., urine toxicology) and pregnancy testing at each visit. Most acute THC studies include such procedures, and their absence or inclusion should be stated.

9. The Methods section would benefit from a more comprehensive description of inclusion and exclusion criteria to allow readers to better assess participant selection and generalizability.

10. While the manuscript states that participants were asked to fast between baseline and 2h post-ingestion, there is no information as to what instructions the participants were given prior to baseline. This is important as fasting ingestion of oral THC has a much sooner onset of effects than that of fed states.

11. While the discussion references plasma THC concentrations from other studies using similar doses, such comparisons should be interpreted cautiously. Differences in formulation, fed versus fasted state, participant characteristics, and other factors can substantially influence absorption and pharmacokinetics, limiting the direct translatability of these comparisons.

**********

what does this mean?). If published, this will include your full peer review and any attached files.

If you choose “no”, your identity will remain anonymous but your review may still be made public.

**Do you want your identity to be public for this peer review?**  For information about this choice, including consent withdrawal, please see our Privacy Policy

Reviewer #2: **Yes:** Arun Prasad

Reviewer #3: No

Reviewer #4: No

**********

[NOTE: If reviewer comments were submitted as an attachment file, they will be attached to this email and accessible via the submission site. Please log into your account, locate the manuscript record, and check for the action link "View Attachments". If this link does not appear, there are no attachment files.]

To ensure your figures meet our technical requirements, please review our figure guidelines: https://journals.plos.org/plosone/s/figures

You may also use PLOS’s free figure tool, NAAS, to help you prepare publication quality figures: https://journals.plos.org/plosone/s/figures#loc-tools-for-figure-preparation.

NAAS will assess whether your figures meet our technical requirements by comparing each figure against our figure specifications.

# Author comment: Author response to Decision Letter 2

**DOI:** 10.1371/journal.pone.0353389.r004

21 Feb 2026

Please see the attached document for our point-by-point response to reviewer comments.

Attachment Submitted filename: Response_to_reviewers_auresp_2.docx

# Peer review: Decision Letter 2

**DOI:** 10.1371/journal.pone.0353389.r005

**Authors:** Souparno Mitra

27 Mar 2026

Dear Dr. McNeil,

Thank you for submitting your manuscript to PLOS ONE. After careful consideration, we feel that it has merit but does not fully meet PLOS ONE’s publication criteria as it currently stands. Therefore, we invite you to submit a revised version of the manuscript that addresses the points raised during the review process.

==============================

**ACADEMIC EDITOR:** **Please address reviewers comments and resubmit for further consideration**

==============================

Please submit your revised manuscript by May 11 2026 11:59PM. If you will need more time than this to complete your revisions, please reply to this message or contact the journal office at plosone@plos.org. When you're ready to submit your revision, log on to https://www.editorialmanager.com/pone/ and select the 'Submissions Needing Revision' folder to locate your manuscript file.

- A letter that responds to each point raised by the academic editor and reviewer(s). You should upload this letter as a separate file labeled 'Response to Reviewers'.
- A marked-up copy of your manuscript that highlights changes made to the original version. You should upload this as a separate file labeled 'Revised Manuscript with Track Changes'.
- An unmarked version of your revised paper without tracked changes. You should upload this as a separate file labeled 'Manuscript'.

If you would like to make changes to your financial disclosure, please include your updated statement in your cover letter. Guidelines for resubmitting your figure files are available below the reviewer comments at the end of this letter.

If applicable, we recommend that you deposit your laboratory protocols in protocols.io to enhance the reproducibility of your results. Protocols.io assigns your protocol its own identifier (DOI) so that it can be cited independently in the future. For instructions see: https://journals.plos.org/plosone/s/submission-guidelines#loc-laboratory-protocols. Additionally, PLOS ONE offers an option for publishing peer-reviewed Lab Protocol articles, which describe protocols hosted on protocols.io. Read more information on sharing protocols at https://plos.org/protocols?utm_medium=editorial-email&utm_source=authorletters&utm_campaign=protocols.

As the corresponding author, your ORCID iD is verified in the submission system and will appear in the published article. PLOS supports the use of ORCID, and we encourage all coauthors to register for an ORCID iD and use it as well. Please encourage your coauthors to verify their ORCID iD within the submission system before final acceptance, as unverified ORCID iDs will not appear in the published article. *Only*  the individual author can complete the verification step; PLOS staff *cannot*  verify ORCID iDs on behalf of authors.

We look forward to receiving your revised manuscript.

Kind regards,

Souparno Mitra, M.D.

Academic Editor

PLOS One

Journal Requirements:

1. If the reviewer comments include a recommendation to cite specific previously published works, please review and evaluate these publications to determine whether they are relevant and should be cited. There is no requirement to cite these works unless the editor has indicated otherwise.

2. Please review your reference list to ensure that it is complete and correct. If you have cited papers that have been retracted, please include the rationale for doing so in the manuscript text, or remove these references and replace them with relevant current references. Any changes to the reference list should be mentioned in the rebuttal letter that accompanies your revised manuscript. If you need to cite a retracted article, indicate the article’s retracted status in the References list and also include a citation and full reference for the retraction notice.

[Note: HTML markup is below. Please do not edit.]

Reviewers' comments:

Reviewer's Responses to Questions

**Comments to the Author**

Reviewer #2: All comments have been addressed

Reviewer #5: (No Response)

**********

2. Is the manuscript technically sound, and do the data support the conclusions?

Reviewer #2: Partly

Reviewer #5: Yes

**********

3. Has the statistical analysis been performed appropriately and rigorously?

Reviewer #2: I Don't Know

Reviewer #5: I Don't Know

**********

4. Have the authors made all data underlying the findings in their manuscript fully available?

The PLOS Data policy

Reviewer #2: Yes

Reviewer #5: Yes

**********

5. Is the manuscript presented in an intelligible fashion and written in standard English?

Reviewer #2: Yes

Reviewer #5: Yes

**********

Reviewer #2: Thanks for making sugessted edits for article- "A six-hour time series of the acute effects of ingested cannabis intoxication on a battery of cognitive-motor tasks"

Reviewer #5: The authors addressed many limitations, however, study could have benefited from a larger, more diverse sample, placebo control, and long-term follow-up. The study did not measure plasma THC concentrations or its metabolites which could provide more precise insights into the relationship between THC levels and intoxication. The three cognitive-motor tasks may not fully capture the breadth of cognitive and motor impairments caused by THC. Using a control group does not necessarily mean that a placebo condition is present. Administering placebo to 7 of 15 participants could have blinded them eliminating expectancy bias(psychological factors vs pharmacological effects of THC)

**********

what does this mean?). If published, this will include your full peer review and any attached files.

If you choose “no”, your identity will remain anonymous but your review may still be made public.

**Do you want your identity to be public for this peer review?**  For information about this choice, including consent withdrawal, please see our Privacy Policy

Reviewer #2: **Yes:** Arun Prasad

Reviewer #5: **Yes:** Anoop Narahari

**********

[NOTE: If reviewer comments were submitted as an attachment file, they will be attached to this email and accessible via the submission site. Please log into your account, locate the manuscript record, and check for the action link "View Attachments". If this link does not appear, there are no attachment files.]

To ensure your figures meet our technical requirements, please review our figure guidelines: https://journals.plos.org/plosone/s/figures

You may also use PLOS’s free figure tool, NAAS, to help you prepare publication quality figures: https://journals.plos.org/plosone/s/figures#loc-tools-for-figure-preparation.

NAAS will assess whether your figures meet our technical requirements by comparing each figure against our figure specifications.

# Author comment: Author response to Decision Letter 3

**DOI:** 10.1371/journal.pone.0353389.r006

22 Apr 2026

Please see the cover letter, which explains that no edits were requested by the reviewers of the last version of the manuscript.

Attachment Submitted filename: Response_to_reviewers_auresp_3.docx

# Peer review: Decision Letter 3

**DOI:** 10.1371/journal.pone.0353389.r007

**Authors:** Souparno Mitra

23 Jun 2026

A six-hour time series of the acute effects of ingested cannabis intoxication on a battery of cognitive-motor tasks

PONE-D-25-56417R3

Dear Dr. McNeil,

We’re pleased to inform you that your manuscript has been judged scientifically suitable for publication and will be formally accepted for publication once it meets all outstanding technical requirements.

Within one week, you’ll receive an e-mail detailing the required amendments. When these have been addressed, you’ll receive a formal acceptance letter and your manuscript will be scheduled for publication.

An invoice will be generated when your article is formally accepted. Please note, if your institution has a publishing partnership with PLOS and your article meets the relevant criteria, all or part of your publication costs will be covered. Please make sure your user information is up-to-date by logging into Editorial Manager at Editorial Manager® and clicking the ‘Update My Information' link at the top of the page. For questions related to billing, please contact billing support.

If your institution or institutions have a press office, please notify them about your upcoming paper to help maximize its impact. If they’ll be preparing press materials, please inform our press team as soon as possible -- no later than 48 hours after receiving the formal acceptance. Your manuscript will remain under strict press embargo until 2 pm Eastern Time on the date of publication. For more information, please contact onepress@plos.org.

Kind regards,

Souparno Mitra, M.D.

Academic Editor

PLOS One

Additional Editor Comments (optional):

Reviewers' comments:

Reviewer's Responses to Questions

**Comments to the Author**

Reviewer #2: All comments have been addressed

Reviewer #5: All comments have been addressed

**********

2. Is the manuscript technically sound, and do the data support the conclusions?

Reviewer #2: Partly

Reviewer #5: Yes

**********

3. Has the statistical analysis been performed appropriately and rigorously?

Reviewer #2: I Don't Know

Reviewer #5: Yes

**********

4. Have the authors made all data underlying the findings in their manuscript fully available?

The PLOS Data policy

Reviewer #2: Yes

Reviewer #5: Yes

**********

5. Is the manuscript presented in an intelligible fashion and written in standard English?

Reviewer #2: Yes

Reviewer #5: Yes

**********

Reviewer #2: Thank you for making suggested edits on article "A six-hour time series of the acute effects of ingested cannabis intoxication on a battery of cognitive-motor tasks"

Reviewer #5: All of my comments about the study design that were already addressed in response to earlier feedback

**********

what does this mean?). If published, this will include your full peer review and any attached files.

If you choose “no”, your identity will remain anonymous but your review may still be made public.

**Do you want your identity to be public for this peer review?**  For information about this choice, including consent withdrawal, please see our Privacy Policy

Reviewer #2: **Yes:** Aeun Prasad MD

Reviewer #5: **Yes:** Anoop Narahari

**********

# Editor report: Acceptance letter

**DOI:** 10.1371/journal.pone.0353389.r008

**Authors:** Souparno Mitra

PONE-D-25-56417R3

PLOS One

Dear Dr. McNeil,

I'm pleased to inform you that your manuscript has been deemed suitable for publication in PLOS One. Congratulations! Your manuscript is now being handed over to our production team.

At this stage, our production department will prepare your paper for publication. This includes ensuring the following:

* All references, tables, and figures are properly cited

* All relevant supporting information is included in the manuscript submission,

* There are no issues that prevent the paper from being properly typeset

You will receive further instructions from the production team, including instructions on how to review your proof when it is ready. Please keep in mind that we are working through a large volume of accepted articles, so please give us a few days to review your paper and let you know the next and final steps.

Lastly, if your institution or institutions have a press office, please let them know about your upcoming paper now to help maximize its impact. If they'll be preparing press materials, please inform our press team within the next 48 hours. Your manuscript will remain under strict press embargo until 2 pm Eastern Time on the date of publication. For more information, please contact onepress@plos.org.

You will receive an invoice from PLOS for your publication fee after your manuscript has reached the completed accept phase. If you receive an email requesting payment before acceptance or for any other service, this may be a phishing scheme. Learn how to identify phishing emails and protect your accounts at https://explore.plos.org/phishing.

If we can help with anything else, please email us at customercare@plos.org.

Thank you for submitting your work to PLOS ONE and supporting open access.

Kind regards,

PLOS ONE Editorial Office Staff

on behalf of

Dr. Souparno Mitra

Academic Editor

PLOS One
